# Profiling Immunological Phenotypes in Individuals During the First Year After Traumatic Spinal Cord Injury: A Longitudinal Analysis

**DOI:** 10.1089/neu.2022.0500

**Published:** 2023-11-30

**Authors:** Debra Morrison, Camille Pinpin, Annette Lee, Cristina Sison, Ashley Chory, Peter K. Gregersen, Gail Forrest, Steven Kirshblum, Susan J. Harkema, Maxwell Boakye, James S. Harrop, Thomas N. Bryce, Jan M. Schwab, Brian K. Kwon, Adam B. Stein, Matthew A. Bank, Ona Bloom

**Affiliations:** ^1^ The Feinstein Institutes for Medical Research, Northwell Health, Manhasset, New York, USA.; ^2^ Donald and Barbara Zucker School of Medicine at Hofstra Northwell, Hempstead, New York, USA.; ^3^ North Shore University Hospital, Manhasset, New York, USA.; ^4^ Tim and Caroline Reynolds Center for Spinal Stimulation, Center for Mobility and Human Engineering Research, West Orange, New Jersey, USA.; ^5^ Department of Physical Medicine and Rehabilitation, Rutgers New Jersey Medical School, Newark, New Jersey, USA.; ^6^ Kessler Institute for Rehabilitation. West Orange, New Jersey, USA.; ^7^ Kentucky Spinal Injury Research Center, School of Medicine, University of Louisville, Louisville, Kentucky, USA.; ^8^ Department of Neurosurgery, School of Medicine, University of Louisville, Louisville, Kentucky, USA.; ^9^ Department of Neurosurgery, Thomas Jefferson University Hospitals, Philadelphia, Pennsylvania, USA.; ^10^ Department of Rehabilitation and Human Performance, Icahn School of Medicine at Mount Sinai Hospital, New York, New York, USA.; ^11^ The Belford Center for Spinal Cord Injury, Spinal Cord Division, Wexner Medical Center, The Ohio State University, Columbus, Ohio, USA.; ^12^ Department of Neurology, Spinal Cord Division, Wexner Medical Center, The Ohio State University, Columbus, Ohio, USA.; ^13^ International Collaboration on Repair Discoveries (ICORD), Department of Orthopaedics, University of British Columbia, Vancouver, British Columbia, Canada.

**Keywords:** functional genomics, human studies, inflammation, RNA sequencing, spinal cord injury

## Abstract

Individuals with SCI are severely affected by immune system changes, resulting in increased risk of infections and persistent systemic inflammation. While recent data support that immunological changes after SCI differ in the acute and chronic phases of living with SCI, only limited immunological phenotyping in humans is available. To characterize dynamic molecular and cellular immune phenotypes over the first year, we assess RNA (bulk-RNA sequencing), protein, and flow cytometry (FACS) profiles of blood samples from 12 individuals with SCI at 0-3 days and at 3, 6, and 12 months post injury (MPI) compared to 23 uninjured individuals (controls). We identified 967 differentially expressed (DE) genes in individuals with SCI (FDR <0.001) compared to controls. Within the first 6 MPI we detected a reduced expression of NK cell genes, consistent with reduced frequencies of CD56^bright^, CD56^dim^ NK cells present at 12 MPI. Over 6MPI, we observed increased and prolonged expression of genes associated with inflammation (e.g. HMGB1, Toll-like receptor signaling) and expanded frequencies of monocytes acutely. Canonical T-cell related DE genes (e.g. FOXP3, TCF7, CD4) were upregulated during the first 6 MPI and increased frequencies of activated T cells at 3-12 MPI. Neurological injury severity was reflected in distinct whole blood gene expression profiles at any time after SCI, verifying a persistent ‘neurogenic’ imprint. Overall, 2876 DE genes emerge when comparing motor complete to motor incomplete SCI (ANOVA, FDR <0.05), including those related to neutrophils, inflammation, and infection. In summary, we identify a dynamic immunological phenotype in humans, including molecular and cellular changes which may provide potential targets to reduce inflammation, improve immunity, or serve as candidate biomarkers of injury severity.

## Introduction

An estimated 300,000 individuals live with traumatic spinal cord injury (SCI) in the United States. Since the 1980s, life expectancy has improved within the first two years after SCI. Unfortunately, longer-term life expectancy has not improved, where individuals with SCI have lower life expectancies than non-injured people(1–3). SCI causes motor and sensory dysfunction, as well as decreased descending supraspinal control of the autonomic nervous system (ANS), resulting in multi-organ system impairments that contribute to serious medical complications. These include blood pressure instability, elevated risk of cardiovascular disease and stroke, and neuropathic pain. Immune system dysfunction after SCI is clinically significant: diseases of the respiratory system (65% of which are characterized as pneumonia) are the leading cause of death, followed by infections (90% of which are characterized as septicemia), and infections are the leading cause of rehospitalization for individuals with SCI throughout their lifetime, with infection risks highest during the first year after injury(3,4). In addition, most individuals with SCI show signs of persistent systemic inflammation, which contributes to the elevated risk of cardiovascular disease and stroke in this population and may also reduce efficacy of neurorehabilitation(5–7). Characterizing the systemic immune phenotype of individuals with SCI, as well as the temporal evolution of cellular and molecular immunological changes during the first year after SCI is thus critical for promoting functional recovery and for reducing morbidity and mortality after SCI.

Previous studies have examined systemic immune system changes in individuals at either the acute phase spanning the first 28 days, with most studies reporting data within the first 7 days after SCI, where these changes have been primarily considered as candidate biomarkers of injury severity(8–15), or in the chronic phase (greater than one year from initial injury) of SCI, where they have been largely considered in the context of inflammation(6,16–24). Correlations have been proposed between immune system changes and injury level (along rostral-caudal axis of the spinal cord) based on changes in ANS function. In particular, persons with SCI rostral to thoracic level 6 (T6), where sympathetic nervous system (SNS) fibers exit the spinal cord and innervate immune organs, are predicted to have the worst immune dysfunction(20,21,25–28). However, several recent observational and interventional studies suggest that ANS regulation of physiological systems is more complex after SCI than previously predicted by injury level or severity. For example, a study of heart rate and blood pressure demonstrated that cardiovascular instability is present in individuals with SCI across injury levels and severities, except for those with low thoracic injuries(29). Notably, increasing data from clinical trials of neuromodulation in persons with chronic SCI demonstrate improvements in autonomic functions that were not predicted by the stimulation site(30–33).

Despite the clinical relevance, systemic immune system parameters have not been profiled longitudinally in individuals during the first year after SCI, when individuals are at greatest risk for sepsis and pneumonia and when they typically achieve the most functional recovery(3,34,35). Here, we conducted a study of the molecular and cellular immunological profiles in individuals throughout the first year after SCI. The primary objective was to delineate changes in circulating inflammatory gene expression compared to uninjured individuals within the first 3 days after SCI and then at three, six, and 12 months post injury (MPI). The second objective was to determine molecular changes that were shared across or distinct at specific times after injury, to identify potentially deleterious factors that may be modifiable at clinically feasible time points that coincide with typical clinical follow-up (3, 6 and 12MPI). The third objective was to characterize changes in the cellular composition of the systemic immune system over time in persons with SCI as compared to persons without SCI. The last objective was to explore the hypothesis that changes in the systemic immune system after SCI correlate with injury characteristics such as severity or level. We present an analysis of immunological outcomes from the first 12 participants who completed at least 6 months of the study. Bulk RNA-Seq of whole blood was used to identify changes in gene expression, multicolor flow cytometry was used to determine relative proportions of major immune cell subsets, and ELISAs were used to determine levels of systemic inflammatory proteins. Data from individuals with SCI was compared to data from a group of uninjured individuals of comparable age and sex distribution, and among individuals with SCI according to injury severity.

## Methods

### Participants

This study was reviewed and approved by the local Institutional Review Boards of Northwell Health and participating institutions. This study was listed on clincaltrials.gov #NCT02731027. Written informed consent was obtained from all individuals, or their proxies, before study enrollment. Participants were recruited from 2016-2020 at Level One Trauma Centers associated with: Northwell Health, The Kessler Institute for Rehabilitation, University of Louisville, Thomas Jefferson University, University of British Columbia, and the Ohio State University Medical Center. All samples included in this analysis were collected prior to the onset of the COVID-19 pandemic. For inclusion in the control group (CTL), individuals without SCI were: (1) >18 years of age, (2) without current or history of cancer, (3) without a history of SCI, autoimmune or neurodegenerative diseases, and (4) were similar in age range and sex distribution as the participants with SCI. Inclusion criteria for participants with SCI were: (1) > 18 years of age, (2) initial SCI 0-3 days prior, (3) classified with an American Spinal Injury Association Impairment Scale (AIS) grade of A to D, and (4) neurological injury level of cervical (C)4-thoracic (T)10. The presence of a SCI was determined by clinical and radiographic presentation. Neurological level of injury (NLI) and severity of injury were classified according to the International Standards for Neurologic Classification of Spinal Cord Injury (ISNCSCI), resulting in the AIS of A, B, C or D. AIS A indicates motor and sensory function below the NLI is absent (motor complete), AIS B indicates some sensory, but not motor, function below the NLI is present (motor complete), AIS C and D indicate motor function is partially preserved below the NLI (motor and sensory incomplete), with greater motor function preserved in AIS D as compared to AIS C(36). Patients with active infections at the time of a study visit were asked to reschedule it as soon as possible and/or to complete any remaining study visits when their infections had resolved.

For this report, samples from individuals with acute motor complete injuries (AIS A or B, N = 6) were analyzed as a group and compared to samples from individuals with acute motor incomplete injuries (AIS C or D, N = 6). While individuals with AIS B injuries have better physical and psychosocial health outcomes than individuals with AIS A injuries at 12 MPI(37), here we included the single individual with an acute AIS B injury in the motor complete group because their biochemical profile clustered with the other samples from individuals with motor complete (AIS A) injuries. Potential participants were excluded if there was documented medical history of cancer, autoimmune disease, other neurologic disease, or for any reason that a physician felt their participation was contraindicated. The data presented here are from the first 12 participants with SCI who had blood samples collected at a minimum of three study visits, including the acute sample.

Blood samples from participants with SCI were scheduled for collection at study visits 0-3 days post injury (DPI, “acute”), and again at 3 MPI, 6 MPI, and 12 MPI. For participants without SCI (CTL), blood samples were obtained once. Due to logistical challenges and medical events, not all blood samples were available for analysis by all three biological outcomes presented here. If an individual had an active infection or pressure injury at the time of a study visit, no blood sample was drawn at that time. The number of participants with SCI included in each analysis were: RNA-sequencing (N = 10), ELISA (N = 12), and flow cytometry (N = 7, only from Northwell Health participants). CTLs were recruited at Northwell Health and their samples were used for RNA-sequencing (N = 9) or flow cytometry (N = 12) analyses.

### RNA-sequencing

Whole blood was collected in PAXgene tubes (Qiagen, Venlo, Netherlands) and stored at -80C until analysis. Total RNA was isolated by QIAcube, using the manufacturer's protocol (Qiagen, Venlo, Netherlands). RNA quality was determined on the Agilent Bioanalyzer, mRNA-Seq libraries were prepared (Illumina TruSeq Stranded Total RNA with RiboZero Globin (Catalog #20020612) and 100 bp paired-end reads were collected on the Illumina HiSeq 2500 platform (Northwell Health). Using Partek Genomics Flow software (St. Louis, MO, USA), trimmed reads were aligned using STAR to the human genome (hg38 genome assembly), filtered for expression >50, normalized using the Trimmed Mean of *M*-values (TMM) method using the edgeR package embedded in Partek Genomics, and log2 transformed. Differential expression of transcripts was determined by one way ANOVA with a fold change greater than 1.5, using the Benjamini–Hochberg FDR <0.05 or <0.001, as indicated (Partek Genomics Flow). For functional analysis of differentially expressed (DE) genes, if multiple transcripts for the same gene symbol were DE, then the transcript with the lowest *p*-value for that gene symbol was included for further analyses. Venn diagrams were created in Venny to determine distinct or shared genes by study visit(38). Functional analysis of DE genes was performed using the bioinformatics platform Enrichr(39). Principal components analysis (PCA) was performed using default parameters (Partek Genomics Flow) for the determination of the component number.

### Flow cytometry

Peripheral blood mononuclear cells (PBMCs) were purified from fresh whole blood using Ficoll Paque Plus (GE Healthcare) and the manufacturer's standard protocol. PBMCs were labeled with fluorescently labeled multicolor antibody panels (Supplementary Table S1) and then fixed with 4% PFA. One-two million cells were stained in each antibody cocktail and approximately 150,000 total events were collected using a BD LSRII Flow Cytometer. Analysis was performed using FlowJo software (FlowJo, LLC). Cells were first gated as leukocytes using forward (FSC) versus side scatter (SSC), and then the singlet population was selected. Cell types and activation states were defined by standard gating strategies (Supplementary Fig. S1).

### Enzyme-linked immunosorbent assays (ELISAs) for protein profiling

Cytokines, chemokines, and growth factors were measured in plasma samples from participants with SCI using commercially available single analyte ELISAs and multiplex bead-based assays. Single analyte ELISAs were used to measure C Reactive Protein (CRP, IBL International #EU59131), and High Mobility Group Box 1 protein (HMGB1, IBL International # ST51011), according to manufacturers' recommended protocols. ELISA data were collected on a SpectraMax plate reader (Molecular Devices) and analyzed using GraphPad Prism 9. A multiplex assay (Bio-Plex Pro Human Cytokine Screening Panel, 48-plex, #12007283) was used to measure an additional 48 cytokines, chemokines, and growth factors. Assays were performed according to manufacturers' recommended protocols. Data was collected on a Bio-Plex 200 system (Bio-Rad) and analyzed using Bio-Plex manager software. Plasma samples were assayed in duplicate. Assay ranges were essentially as specified by the manufacturers. Kit controls were within expected concentration ranges, %CV across all samples and all analytes were 7.3%, with an average recovery of 100% and a range of 70-130%. For statistical analysis, measurements below the limit of detection were assigned a value of half the lowest detectable value (LLOQ). Analytes (N = 34/50) that were detectable in >50% of the samples were compared between samples from participants with SCI (motor complete vs. incomplete injuries).

## Results

### Participants' characteristics

Clinical and demographic variables are shown in Tables 1 and 2. Participants in both SCI and non-SCI groups were predominantly male and were of comparable age, which is consistent with the traumatic SCI population. Among the SCI patients the mechanisms of injury (MOI) were: falls (N = 9), motor vehicle crash (MVC, N = 2), or sports (N = 1). Acutely after SCI, the NLI of participants with SCI was either cervical (N = 9) or thoracic (N = 3), with most participants injured rostral to T6, the level of sympathetic outflow to immune organs(40). At study entry, participants with SCI had injury severities reflective of US national data, with most injuries classified as AIS grade A or D: A (N = 5), B (N = 1), C (N = 2), D (N = 4).

**Table 1.** Demographic Characteristics of Individuals With SCI and Without SCI (CTL)

ID	Group (SCI or CTL)	Age	Gender	MOI	AIS grade (acute)	NLI (acute)
1	SCI	68	F	Fall	D	C5
2	SCI	83	M	Fall	D	C8
3	SCI	54	M	Fall	D	C5
4	SCI	43	M	Fall	C	C4
5	SCI	24	M	Fall	C	T4
6	SCI	31	M	MVC	A	C5
7	SCI	28	M	Fall	A	C6
8	SCI	63	M	MVC	A	T3
9	SCI	63	M	Fall	B	C5
10	SCI	59	M	Fall	A	C6
11	SCI	28	M	MVC	A	T9
12	SCI	62	M	Sports	D	C5
13	CTL	49	M			
14	CTL	33	F			
15	CTL	33	M			
16	CTL	51	M			
17	CTL	49	M			
18	CTL	31	M			
19	CTL	28	M			
20	CTL	62	M			
21	CTL	61	M			
22	CTL	32	F			
23	CTL	48	F			
24	CTL	28	M			
25	CTL	36	M			
26	CTL	33	M			
27	CTL	35	F			
28	CTL	34	M			
29	CTL	28	F			
30	CTL	63	M			
31	CTL	65	M			
32	CTL	60	M			
33	CTL	53	M			
34	CTL	59	M			
35	CTL	56	M			

AIS, American Spinal Injury Association Impairment Scale; CTL, control; MOI, method of injury; MVC, motor vehicle crash; NLI, neurological level of injury; SCI, spinal cord injury.

**Table 2.** Clinical Characteristics of Individuals With SCI

ID		AIS grade	NLI
1	Acute	D	C5
	3 MPI	D	C5
	6 MPI	D	C3
	12 MPI	D	C6
2	Acute	D	C8
	3 MPI	D	C1
	6 MPI	D	C7
	12 MPI	D	T5
3	Acute	D	C5
	3 MPI	D	T1
	6 MPI	D	C5
	12 MPI	D	T1
4	Acute	C	C4
	3 MPI	D	C5
	6 MPI	D	C5
	12 MPI	D	C4
5	Acute	C	T4
	3 MPI	D	T4
	6 MPI	D	L2
6	Acute	A	C5
	3 MPI	A	C6
	6 MPI	A	C5
	12 MPI	B	C4
7	Acute	A	C6
	3 MPI	A	C6
	6 MPI	B	C5
8	Acute	A	T3
	3 MPI	A	T3
	6 MPI	A	T3
	12 MPI	A	T2
9	Acute	B	C5
	3 MPI	C	C4
	6 MPI	C	C5
10	Acute	A	C6
	3 MPI	A	C2
	6 MPI	A	C4
	12 MPI	A	C4
11	Acute	A	T9
	3 MPI	A	T6
	6 MPI	B	T6
	12 MPI	C	T6
12	Acute	D	C5
	6 MPI	D	C2

NLI was determined by ISNCSCI exam at each time-point.

AIS, American Spinal Injury Association Impairment Scale; ISNCSCI, International Standards for Neurological Classification of Spinal Cord Injury; MPI, months post-injury; NLI, neurological level of injury; SCI, spinal cord injury.

### Dynamic changes in gene expression over time after SCI

**FIG. 1.**
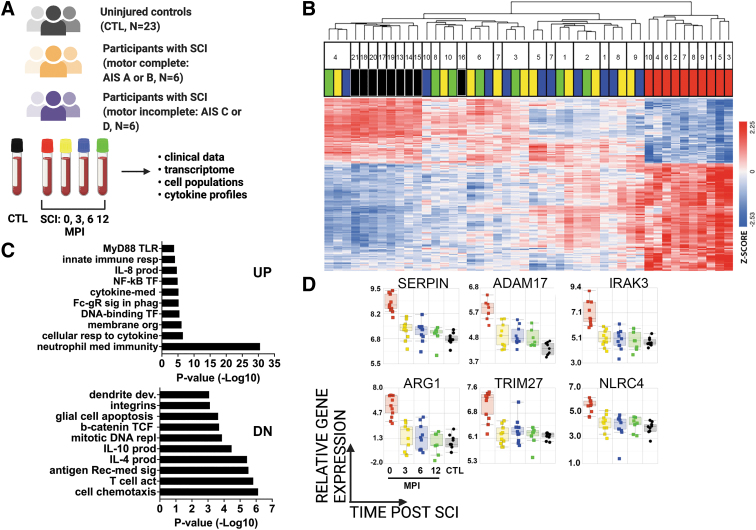
Longitudinal profiling of whole blood gene expression after SCI. **(A)** Overview of cohort, including participants without SCI (CTLs), and participants with moderate or severe SCI. Injury severity determined by AIS grade, where A or B indicates motor complete and C or D indicates motor and sensory incomplete injuries. **(B)** Dendrogram of two-way unsupervised hierarchical clustering shows the first major branch point differentiates between samples collected at the acute visit and all other study visits (*n* = 967 filtered gene transcripts, 1.5-fold difference, FDR <0.001, Benjamini-Hochberg adjusted). Participant ID numbers are shown above color-coded legend that indicates study visits (red = acute, yellow = 3 MPI, blue = 6 MPI, green = 12 MPI, black = uninjured CTLs). Heat map shows relative gene expression; color corresponds to Z-score (blue: downregulation, red: upregulation). Corresponding gene list with relative expression values is shown in Supplementary Table S2. **(C)** GOBP enrichment analysis of 756 genes with positive Z-scores (upregulated) and 456 genes with negative Z-scores (downregulated) at the acute time-point. **(D)** Temporal expression patterns of select genes from upregulated GOBP categories: neutrophil pathways (SerpinB1, ARG1), cytokine stimulation and signaling (ADAM17, IRAK3), and NFκB signaling (TRIM27, NLRC4). AIS, American Spinal Injury Association Impairment Scale; CTL, control; FDR, false discovery rate; GOBP, Gene Ontology biological process; IL, interleukin; MPI, months post-injury; SCI, spinal cord injury; TF, transcription factors; TLR, Toll-like receptor.

We performed RNA-Seq on whole blood samples available from participants with SCI and from a group of participants without SCI (CTL) of similar age range and sex distribution (Fig. 1A). Differential expression (DE) of genes from CTL and SCI groups was determined by ANOVA (Benjamini-Hochberg adjusted FDR of <0.001), which identified 1212 unique transcripts (967 genes) that changed in expression at any point over time after SCI (Fig. 1B, Supplementary Table S2). Hierarchical clustering of DE genes showed that acute SCI samples clustered away from all other samples (Fig. 1B). In the dendrogram branch containing all other samples, most CTL samples were clustered together (Fig. 1B). Among samples from post-acute SCI visits, a major organizing influence was the participant, with most samples from an individual clustering together.

We next performed Gene Ontology Biological Process (GOBP) analyses to determine the functional categories enriched among DE genes at the acute time point after SCI (Fig. 1C). GOBP analysis of the upregulated DE genes included several significantly enriched categories related to neutrophil function, cytokine signaling, NF-κB activation, and FcR signaling. GOBP analysis of downregulated DE genes included several significantly enriched categories related to the production of IL-4 and IL-10, cytokines with anti-inflammatory properties, as well as categories related to T cell signaling. Although there is considerable crosstalk between the pathways illustrated and several genes play important roles in more than one pathway, genes of interest related to neutrophil function, cytokine signaling, and NF-κB transcription are shown to illustrate relative changes in gene expression during the first year after SCI (Fig. 1D). For example, arginase 1 (ARG), the enzyme that degrades arginine, is anti-inflammatory when expressed by monocytes and promotes suppression of T-cell and NK-cell proliferation and cytokine secretion(41–43). Toll-like receptor (TLR) genes, which are pattern recognition receptors (PRR) critical for immune responses(44,45), are included in many GO categories, while IL-1R-associated kinase family member, IRAK3, belongs to both pattern recognition receptor and NF-κB related categories(46). These genes were most highly expressed acutely after SCI, and decreased at later time points, with the lowest expression in CTL samples.

Similarly, PCA of unique DE transcripts from participants with SCI compared to CTLs also revealed that acute SCI samples were most distinct from CTL samples (Supplementary Fig. S2 A1-4). In agreement with the ANOVA analysis, GOBP analysis of the top 175 genes in PC1 ranked by Eigenvalue which were downregulated in acute SCI samples compared to CTL samples were mostly related to IL-4, Wnt signaling and T cell signaling (Supplementary Fig. S2B). Moreover, analysis of the top 175 genes in PC1 using the Human Gene Atlas platform showed enrichment in genes related to CD4+ and CD8+ T cells (Supplementary Fig. S2C).

**FIG. 2.**
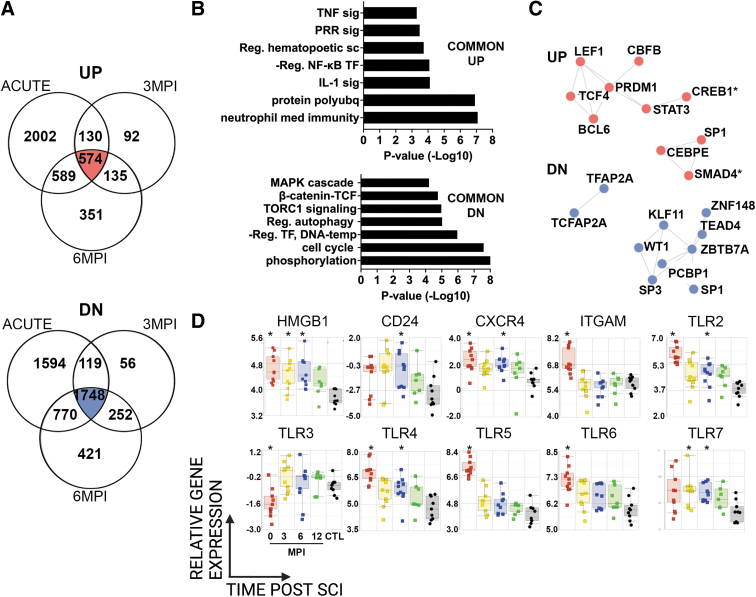
Pairwise comparison of gene expression profiles during the first year after SCI. Gene expression profiles were compared pairwise between SCI samples at acute, 3 MPI, or 6 MPI study visits and samples from participants without SCI. **(A)** Venn diagrams show number of DE genes unique to each study visit or shared among visits: top, upregulated (red); bottom, downregulated (blue). **(B)** GOBP categories that were enriched in DE genes common across acute, 3 MPI, and 6 MPI study visits are shown (top: upregulated, bottom: downregulated). **(C)** TF identified by TRANSFAC and JASPAR databases accessed in the Enrichr platform that were predicted to bind enriched DE genes common across acute, 3 MPI, and 6 MPI study visits are shown (top: up, bottom: down; *indicates mouse TF; all other TF are human). **(D)** Temporal expression patterns of genes of interest from enriched GOBP categories highlight the endogenous TLR ligand HMGB1, its receptors, and other TLRs. Boxes indicate range of Q1 to Q3, line indicates median, and whiskers indicate minimum to maximum. Color-coded legend indicates study visits (red = acute, yellow = 3 MPI, blue = 6 MPI, green = 12 MPI, black = uninjured CTLs); *indicates *p* < 0.02 in pairwise comparison with controls (ANOVA). ANOVA, analysis of variance; CTL, control; DE, differentially expressed; GOBP, Gene Ontology biological process; HMGB1, high mobility group box 1; MPI, months post-injury; SCI, spinal cord injury; TF, transcription factors; TLR, Toll-like receptor.

To identify changes at specific time points after SCI compared to CTLs, we performed a pairwise comparison of gene expression profiles at different study visits (ANOVA, Benjamini Hochberg corrected FDR <0.05). Compared to CTLs, there were 3295 up- and 4231 downregulated DE genes acutely after SCI; 931 up- and 2175 downregulated DE genes at 3 MPI; and 1649 up- and 3191 downregulated DE genes at 6 MPI (Fig. 2A). There were 574 up- and 1748 down-regulated DE genes in common among acute, 3 MPI and 6 MPI samples (Fig. 2A). Upregulated DE genes were enriched in categories related to NF-kB transcription, TLR signaling, neutrophil function, and others (Fig. 2B). Conversely, common downregulated DE genes were enriched in categories related to regulation of the cell cycle, negative regulation of transcription, phosphorylation, histone modification, TORC1 signaling, and TCF signaling (Fig. 2B). We next used TRANSFAC/JASPAR analysis in the Enrichr platform to identify transcription factors (TF) that control up- or down-regulated DE genes in common among acute, 3MPI and 6MPI samples (Fig. 2C). TF of upregulated DE genes included the potent T cell regulator LEF1, a master regulator of hematopoiesis CBFB, the basic-helix-loop-helix TF TCF4, (also known as immunoglobulin transcription factor 2, ITF-2), and STAT3, which is activated by cytokines and chemokines signaling, among others. TF of downregulated DE genes included the zinc finger TF SP1 that regulates immune responses and other cellular activities, the pro-apoptotic KLF11, and others.

Previously, we showed that the endogenous protein HMGB1, a pro-inflammatory alarmin, was upregulated in separate cohorts of individuals with acute or chronic SCI(24). Here, the expression of HMGB1 was significantly elevated at acute, 3 MPI and 6 MPI compared to CTLs (Fig. 2D). Cell surface receptors for HMGB1, including TLR2, TLR4, and CXCR4, were significantly elevated at acute and 6 MPI compared to CTLs. Expression of other cell surface receptors for HMGB1, TLR5 and TLR6, as well as the integrin ITGAM (CD11b), which promotes extracellular release of HMGB1(47), were significantly elevated acutely after SCI. Intracellular TLRs were also modulated by SCI. TLR3 was significantly decreased acutely after SCI, while TLR7 was elevated at 3 MPI and 6 MPI. CD24 was significantly elevated at 6 MPI compared to CTL (Fig. 2C).

We also examined functional enrichment of genes that were uniquely differentially expressed at acute, 3 MPI and 6 MPI study visits, compared to CTLs (Fig. 2A). As with genes shared across time points, GOBP analysis of upregulated DE genes at the acute study visit included categories related to TLR signaling, neutrophil function and cytokine signaling (Supplementary Fig. S3A). At 3MPI, GOBP analysis of upregulated DE genes included categories related to cytokinesis, the cell cycle, macromolecular biosynthesis, and DNA replication (Supplementary Fig. S3B). At 6MPI, GOBP analysis of upregulated DE genes included categories related to transcription, myeloid cell development and adaptive immune responses (Supplementary Fig. S3C). As with genes shared across time points, GOBP analysis of downregulated DE genes at the acute study visit included categories related to ribosome signaling, B and T cell signaling, DNA repair and others (Supplementary Fig. S3D). At 3MPI, GOBP analysis of downregulated DE genes included categories related to immune activation, proteasome signaling, NK cells and others (Supplementary Fig. S3E). At 6MPI, GOBP analysis of downregulated DE genes included categories related to neutrophil function, cytokine responses, myeloid differentiation, and others (Supplementary Fig. S3F).

### SCI has profound effects on natural killer (NK) cell populations

**FIG. 3.**
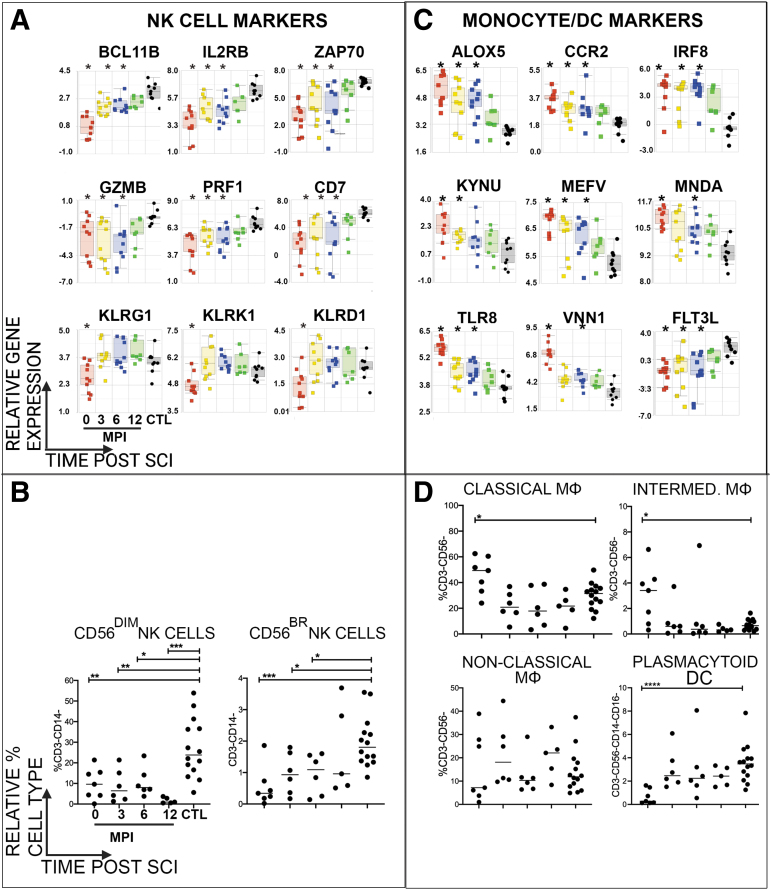

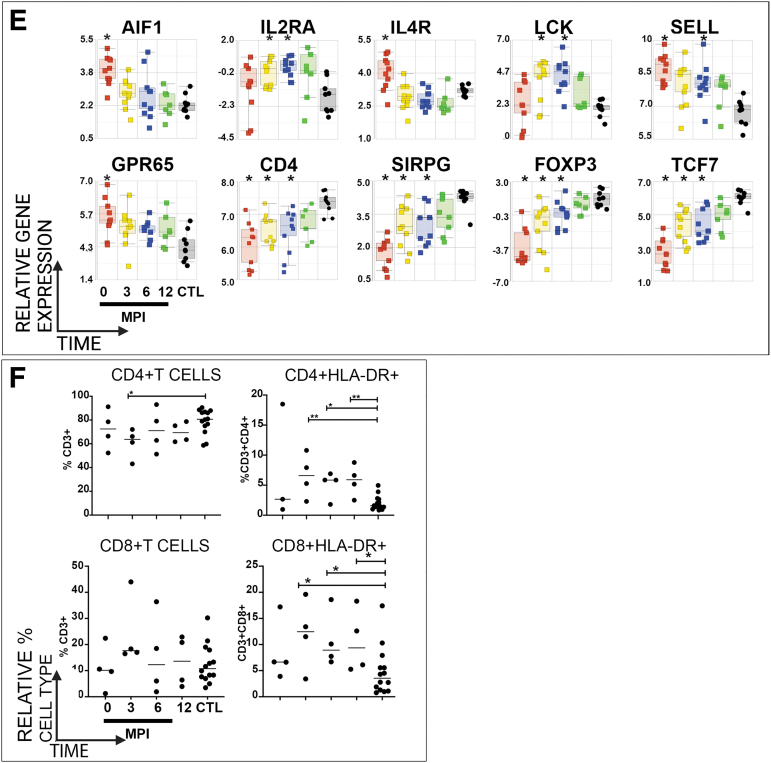
Longitudinal profiling of immune cell subsets of participants after SCI. Relative expression of genes of interest characteristic of **(A)** NK cells, **(C)** monocytes and DCs, and **(E)** T-cell subsets in participants with SCI compared with CTLs. Boxes indicate range of Q1 to Q3, line indicates median, and whiskers indicate minimum to maximum. Color-coded legend indicates study visits (red = acute, yellow = 3 MPI, blue = 6 MPI, green = 12 MPI, black = uninjured CTLs); *indicates *p* < 0.02 in pairwise comparison with controls (ANOVA). Flow cytometry was performed to determine relative distribution of immune cell subsets in participants with SCI compared with CTLs: (C) Percentage of CD56+^dim^ (left) and CD56+^bright^ (right) NK cells are significantly decreased after SCI. **(D)** Percentage of classical and intermediate monocytes are increased acutely, whereas plasmacytoid DCs are decreased acutely after SCI compared with uninjured CTLs. **(F)** Percentage of total CD4+ T cells is significantly decreased at 3 MPI (left upper); percentage of activated CD4+ T cells is increased at 3 MPI, 6 MPI, and 12 MPI after SCI (right upper); percentage of CD8+ T cells is unchanged after SCI (left lower); percentage of activated CD8+ T cells is increased at 3 MPI, 6 MPI, and 12 MPI after SCI (right lower). For flow cytometry, Mann-Whitney U test was performed to determine significance, defined as *p* < 0.05, with *p*-values shown: *p = 0.01–0.05, **p = 0.001–0.01, ***p = 0.0001–0.001, and ****p < 0.0001. ANOVA, analysis of variance; CTL, control; DCs, dendritic cells; MPI, months post-injury; NK, natural killer; SCI, spinal cord injury.

Prior studies have shown reduced numbers, impaired function and reduced gene expression of NK cells in individuals with chronic (more than one year from initial injury) SCI(17,19,20,22). Here, we identified several canonical NK cell genes that were DE within the first year after SCI compared to CTLs (Fig. 3A). These included granzyme B, perforin 1, and Killer Cell Lectin receptor genes. We also noted several examples of signal transduction DE genes expressed by NK cells and T cells, such as IL2RB, the costimulatory molecule CD7, and the tyrosine kinase ZAP70(48–51). Several of the NK cell genes were downregulated acutely and then increased in expression during the first year after SCI. By flow cytometry, we profiled the frequency of circulating NK cells in SCI and CTL participants, which has been shown to be decreased in individuals with SCI(19,20,52). CD56^dim^ NK cells, which are cytolytic and typically the major subset in the periphery, was the major subset in both SCI and CTL samples (Fig. 3B). A significant decrease in the cytolytic CD56^dim^ and cytokine-producing CD56^bright^ NK cell subsets was observed acutely after SCI compared to CTL (Fig. 3B). The reduction in systemic NK cell frequency was maintained during the first year after SCI, particularly in the CD56^dim^ subset (Fig. 3B).

### Monocytes and Dendritic cell populations were affected acutely after SCI

Whole blood cell counts (WBC) were obtained from clinical core laboratory values in participants with SCI during acute hospitalization (Table 3). As expected (11,12,27,53), compared to reference values, WBCs were abnormally high in half of the participants with SCI. Specifically, neutrophils were abnormally elevated in 67% (N = 8) of participants, of which a subset of participants (25%, N = 3) also had elevated monocytes.

**Table 3.** Whole Blood Cell Counts of Individuals With SCI at Acute Study Visit

	WBC	Eosi	Mono	Neu	Lymph
Reference range (K/uL)	4.8-10.8	0.0-0.5	0.0-0.8	1.8-8.0	1.0-4.8
Participant ID					
1	14.6^a^	0.1	0.7	12.3^a^	1.5
2	12.2^a^	0.1	0.3	10.7^a^	1.2
3	9.0	0.1	0.7	6.6	1.6
4	6.2	0.1	0.4	3.2	2.5
5	9.7	0	0.8	8.1^a^	0.7^b^
6	6.7	0.1	0.5	3.8	2.1
7	15.8^a^	0.0	1.4^a^	13.4^a^	0.7^b^
8	9.4	0.0	0.7	8.3^a^	0.3^b^
9	19.1^a^	0.0	1.2^a^	17.1^a^	0.6^b^
10	9.3	0.0	0.6	7.7	0.9^b^
11	22.4^a^	0.1	1.4^a^	13.9^a^	6.9^b^
12	13.4^a^	0.0	0.6	12.5^a^	0.3^b^

^a^ Above reference range; ^b^below reference range.

Eosi, eosinophils; Lymph, lymphocytes; Mono, monocytes; Neu, neutrophils; SCI, spinal cord injury; WBC, white blood cell counts.

A variety of changes in relative monocyte proportion or gene expression have been observed in individuals with acute SCI according to level of injury or injury severity (11,12). Here, compared to CTL, we identified increased expression of many genes associated with (but not exclusive to) monocyte activation (54) acutely after SCI, some of which remained elevated during the first 6MPI. These included ALOX5 that catalyzes leukotriene formation(55), the chemokine receptor CCR2(56), and the endosomal pattern recognition receptor TLR8, which is highly expressed in monocytes and recognizes ssRNA, including influenza viruses(57). We also noted a significant increase in expression of MEFV, otherwise known as “pyrin”, which is an intracellular pattern recognition receptor, within the first 6MPI (Fig. 3C).

We measured the proportions of classical (CD14+CD16-) and intermediate monocytes (CD14+CD16+) and found that classical monocytes were the major subset in both SCI and CTL samples (Fig. 3D). Both classical and intermediate monocyte populations were significantly elevated acutely after SCI, and then returned to frequencies similar to CTL over time (Fig. 3D). However, median fluorescence intensities (MFI) of HLA-DR on the surface of these elevated monocyte subsets were significantly decreased (637 ± 112 vs. 1575 ± 166, P < 0.001 classical monocytes and 4134 ± 583 vs. 8598 ± 810 P < 0.0003 intermediate monocytes, acute SCI vs. CTL respectively). No differences were observed with non-classical monocytes (CD14-/loCD16+).

Dendritic cells (DCs) are innate immune cells that are critical for maintaining immunity against pathogens: myeloid DCs (mDCs) are highly potent antigen presenting cells and plasmacytoid DCs (pDCs) as strong producers of IFN-alpha in response to viral infections. mDCs and pDCs are broadly distinguished by cell surface expression of lineage markers CD123 and CD11c, respectively. We identified DE genes associated with pDCs or mDCs after SCI: expression of FLT3L, which is critical for DC development(58,59)was reduced significantly compared to uninjured controls, while the transcription factor IRF8, which regulates myeloid cell differentiation and promotes DC development(60), was significantly increased at 0, 3 MPI and 6 MPI after SCI compared to CTL (Fig. 3C). Compared with CTL participants, there was no difference in the proportion of circulating mDCs after SCI, which was the majority subset in both groups (data not shown). However, the relative proportion of circulating pDCs was significantly reduced acutely after SCI (Fig. 3D). The relative proportions of B cells (CD19+) and major B cell subtypes (switched memory B (CD27+IgD-), plasmablasts (CD27+IgD-CD38++CD27++), non-switched (CD27+IgD+), naïve (CD27-IgD+ CD38-CD24-) transitional (CD27-IgD+ CD38+CD24+) and double negative B (CD27-IgD-) cells were profiled by flow cytometry, but no significant differences in their frequencies were observed between groups (data not shown).

### The proportion of activated CD4+ and CD8+ T cell subsets was increased after SCI

Lymphopenia has been observed in individuals with acute SCI(12,27). During acute hospitalization, clinical WBC lab values for lymphocytes were abnormally reduced in seven participants with SCI (Table 3). We observed DE genes characteristic of CD4+ and CD8+ T cells within the first 6MPI after SCI, compared to CTLs (Fig. 3E). Genes linked to the development, proliferation and survival of T cells and regulatory T cells, including the T cell receptor CD4, the transcription factors FOXP3 and TCF7, as well as the signaling factor SIRPG, were downregulated at 0, 3MPI and 6MPI. Also, several genes linked to T cell activation were upregulated acutely after SCI (Fig. 3E). These included examples such as the AIF, IL2RA, IL4R, LCK, SELL, and GPR65.

Flow cytometry of T cell populations reflected greater activation of both CD4+ and CD8+T cell subsets after SCI. In agreement with previous studies and the downregulation of T cell related genes, compared to CTL samples, the relative proportion of total T cells (CD3+) was modestly reduced within the first 3MPI after SCI (data not shown)(21,61). The frequency of CD4+ T cells was reduced at 3MPI compared to CTLs, while the frequency of CD8+T cells was unchanged (Fig. 3F). However, as suggested by elevation of genes related to T cell activation, elevated HLA-DR levels indicated an increase in the proportions of activated CD4+ and CD8+ T cells at 3 MPI, 6 MPI and 12 MPI, compared to CTL (Fig. 3F).

### Injury severity during the first year after SCI correlates with blood gene expression profiles

**FIG. 4.**
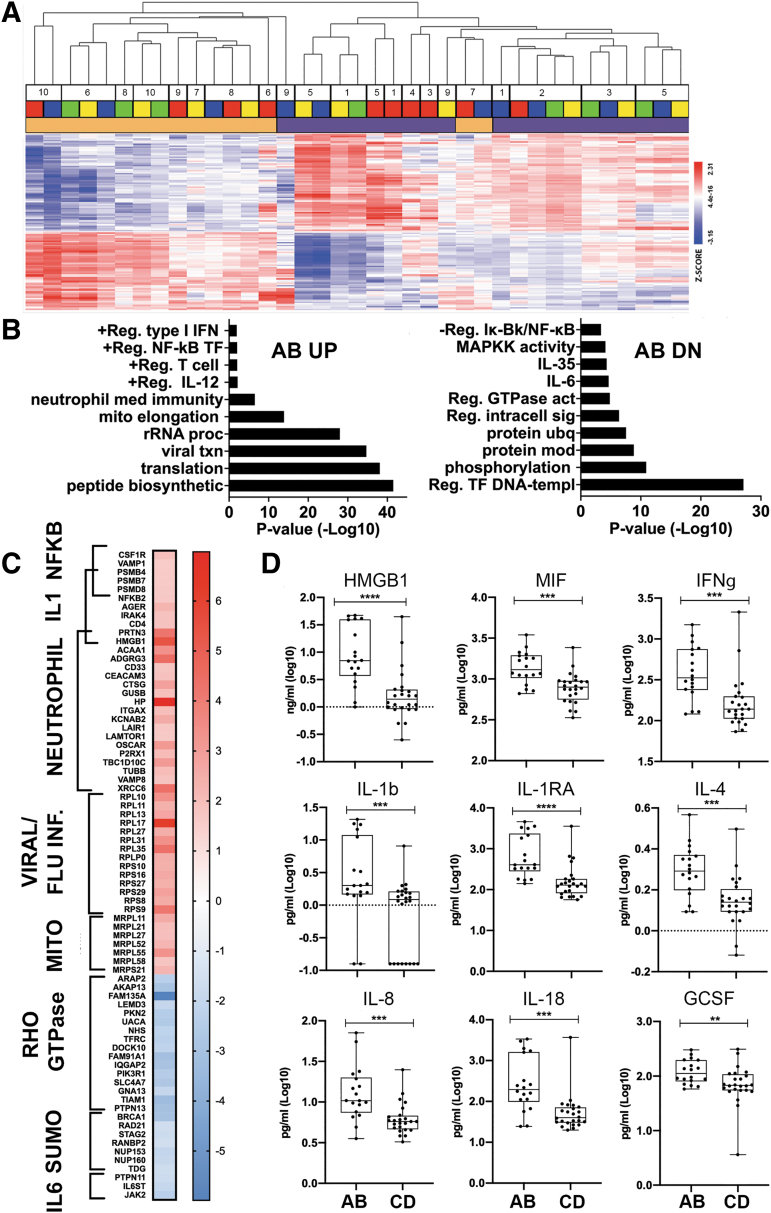
Injury severity of SCI influences whole blood gene expression profiles. **(A)** Dendrogram shows unsupervised two-way hierarchical clustering of DE genes according to AIS grade (*n* = 2876 unique genes, 1.5-fold change in expression, FDR <0.05, Benjamini-Hochberg adjusted). Participant ID numbers are shown above color-coded legend that indicate study visits (red = acute, yellow = 3 MPI, blue = 6 MPI, green = 12 MPI, black = uninjured CTLs) and AIS grade (orange: AIS A or B, purple: AIS C or D). Heat map shows relative gene expression by Z-score (blue: downregulation, red: upregulation). A corresponding gene list with relative expression values is shown in Supplementary Table S2. **(B)** GOBP categories enriched in genes that are upregulated or downregulated according to AIS grade. **(C)** Heat map (log_2_ fold change) of significantly changed genes related to NF-kB activation of B cells signaling (NFKB), IL-1 signaling (L-1), neutrophil degranulation (neutrophil), viral mRNA translation/influenza infection (viral/flu inf.), mitochondrial translation (Mito), Rho GTPase cycle (RHO GTPase), SUMOylation DNA damage and repair (SUMO), and IL-6 signaling (IL-6) in participants with AIS AB injuries compared with AIS CD injuries. **(D)** Elevated systemic inflammatory proteins in samples from participants with AIS AB or AIS CD injuries, measured by ELISA. Inflammatory markers, cytokines, and chemokines, plotted as log10 concentrations, are graphed according to injury severity. Boxes indicate a range of Q1 to Q3, line indicates median, and whiskers indicate minimum to maximum. Symbols represent individual participants at any time-point. Mann-Whitney U test was performed to determine significance, defined as *p* < 0.05, with *p*-values shown: *p = 0.01–0.05, ** *p* = 0.001–0.01, ***p = 0.0001–0.001, and ****p < 0.0001. AIS, American Spinal Injury Association Impairment Scale; CTL, control; FDR, false discovery rate; DE, differentially expressed; ELISA, enzyme-linked immunosorbent assay; GOBP, Gene Ontology biological process; IL, interleukin; MPI, months post-injury; Rho GTPase, Rho guanosine triphosphate; SCI, spinal cord injury.

We next investigated if and how injury severity correlated with changes in systemic immune gene expression. Within samples collected at any study visit from participants with SCI, we identified 2875 unique genes that were significantly differentially expressed between samples from motor complete or incomplete injuries, of which 1422 were up- and 1454 were down-regulated in the motor complete group (ANOVA, FDR <0.05, 1.5-fold change, Fig. 4A). Hierarchical clustering of AB vs CD samples illustrates the strong relationship of samples by motor completeness and then by individual. GOBP analysis of DE genes that were upregulated in motor complete (AIS A or B) vs. incomplete (AIS C or D) samples, included genes related to neutrophils, mitochondrial function, T cell function, and NF-κB activation (Fig. 4B). Categories of DE genes that were downregulated in samples obtained from motor complete injuries were related to regulation of transcription, signal transduction, IL-6 signaling, MAPK signaling, and inhibition of NF-κB (Fig. 4B). A heat map highlights the relative expression of DE genes enriched (up- or down-regulated) in acute motor complete samples, which were related to NF-κB activation, neutrophil function, RHO GTPase, IL-6 signaling, and mitochondrial genes (Fig. 4C).

To expand the analysis of inflammatory mediators that may correlate with injury severity during the first year after SCI, we analyzed levels of inflammatory proteins in plasma samples across all time points from participants with SCI. We identified 20 inflammatory proteins that were significantly different in samples from motor complete or incomplete injuries (Fig. 4D, Table 4), of which 17 were elevated in samples from participants with motor complete injuries. Among these, only the endogenous TLR2/4 ligand HMGB1, was also upregulated at the transcriptional level in samples from participants with motor complete injuries.

**Table 4.** Inflammatory Mediators That Are Significantly Different in Samples from AIS A or B vs. C or D Injuries

Factor	*P*-value	Median		Min		Max		IQR	
		A or B	C or D	A or B	C or D	A or B	C or D	A or B	C or D
HMGB1	<0.0001	7.0	1.4	1.0	0.3	47.2	44.5	35.9	1.2
FGFb	0.0011	32.9	24.3	22.0	1.7	91.1	100.0	22.0	6.04
GCSF	0.0062	113.1	67.5	116.5	3.6	302.7	310.9	116.5	53.3
HGF	0.0473	331.0	280.0	244.0	165.0	3547.0	2957.0	244.0	152.0
IFN-?	0.0002	335.5	138.4	513.8	73.2	1497.0	2139.0	513.8	91.7
IL-1b	0.0009	2.0	1.2	10.5	0.1	20.7	8.1	10.5	1.5
IL-1ra	<0.0001	405.0	129.0	141.0	57.0	4579.0	3546.0	2101.0	101.0
IL-2ra	0.0029	67.0	46.0	40.0	20.0	205.0	160.0	52.0	29.0
IL-4	0.0004	2.0	1.4	1.2	0.8	3.7	3.1	0.8	0.4
IL-6	0.0127	6.1	3.1	0.2	0.2	264.0	19.0	11.5	3.4
IL-8	0.0004	10.4	5.8	3.6	3.2	71.0	25.0	12.6	2.2
IL-16	0.0470	48.3	22.0	0.8	0.8	741.4	769.7	479.3	25.0
IL-18	0.0001	196.8	40.1	24.6	19.8	3359.0	3678.0	1527.2	41.3
*IP-10*	*0.0163*	*160.6*	*332.8*	*59.4*	*38.3*	*639.2*	*2879.0*	*258.3*	*893.8*
MCSF	0.0011	39.7	15.1	7.8	7.1	213.8	285.6	93.0	9.7
MIF	0.0003	1304	794.4	662.9	335.4	3457.0	2422.0	909.0	384.2
*MIG*	*0.0436*	*101.1*	*135.9*	*30.7*	*69.5*	*669.4*	*2565.0*	*159.6*	*353.6*
MIP-1a	0.0165	1.8	1.4	1.1	0.8	5.8	2.8	1.3	0.6
PDGFbb	0.0042	1062.0	555.0	385.0	273.0	2146.0	2315.0	745.0	238.0
*RANTES*	*0.0032*	*4637.0*	*7715.0*	*1432.0*	*2548.0*	*8087.0*	*14971.0*	*4161.0*	*3626.0*

Units = pg/mL except HMGB1 = ng/mL. Italics = downregulated; *p*-value from Mann-Whitney U test.

AIS, American Spinal Injury Association Impairment Scale; HMGB1, high mobility group box 1; IFN, interferon; IL, interleukin; IQR, interquartile range.

### Discussion

Our longitudinal study of individuals with SCI elucidated critical changes in whole blood gene expression and immune cell types during the first year after injury. Clinical and demographic characteristics of participants in this cohort reflected national statistics: most injuries occurred at the cervical level, the most common mechanisms of injury were MVC and falls, and the injury severity was classified mostly as AIS A or D(3).

We first examined the effect of time after injury on gene expression, which revealed that samples collected acutely after injury were distinct from all other samples (uninjured or SCI). In several studies pioneered by Kwon and colleagues, as well as others, changes were identified in inflammatory mediators in CSF or in whole blood gene expression during acute hospitalization after SCI(8–11,62–64). In these studies and here, genes related to neutrophil function were upregulated, while genes related to adaptive immunity were downregulated. In addition, genes related to NF-κB signaling, FcR signaling, cytokine signaling, and neutrophil responses were upregulated acutely, while genes related to the anti-inflammatory cytokines IL-4 and IL-10, and genes related to T cell function were downregulated. Many of these same pathways were differentially expressed acutely after general trauma(65).

Pairwise analysis identified more than 500 upregulated and more than 1700 downregulated differentially expressed genes that were shared across the first 6 months after SCI compared to uninjured controls. Shared DE genes that were increased after SCI included pathways related to NF-κB transcription, neutrophil activation, and TLR-signaling, among others. Shared DE genes that were decreased after SCI included pathways related to the T-cell regulating transcription factor TCF, mTORC1 that regulates protein synthesis, autophagy, the cell cycle, and post-translational modifications like phosphorylation. Many of the TF that regulate shared DE genes that were up- or down-regulated across the first 6 MPI have known roles in hematopoietic cell development or activation. Perhaps most notable among TF identified regulators of the shared upregulated DE genes was LEF1, a master regulator of T cell identity and maintenance. Conversely, it was remarkable that SP1, a TF which is critical for terminal differentiation in hematopoiesis, and its paralogue SP3, were identified as regulators of shared downregulated DE genes(66,67). This is consistent with a recent report from Popovich and colleagues of acquired bone marrow failure syndrome after SCI in a murine model of SCI(68).

Interestingly, we observed an enrichment of genes upregulated after SCI that are related to TLR signaling. As is well-known, TLR genes are highly conserved pattern recognition receptors that recognize intracellular and extracellular pathogens(69,70). The endogenous TLR4 ligand, HMGB1, is a potent alarmin that can be actively secreted from immune cells or passively released by dying cells in the setting of injury, infection, or inflammation(71). We previously demonstrated significantly elevated systemic levels of HMGB1 in independent cohorts of individuals with acute or chronic SCI, while others have shown that in preclinical models of SCI, administration of anti-HMGB1 therapies protect against histological damage and neuropathic pain(24,72,73). Based on the elevation of HMGB1 and many of its related receptors within the first six months after SCI, and the growing number of potential therapeutic interventions targeting TLR or HMGB1, TLR signaling may warrant further investigation in SCI(74,75).

Furthermore, we identified other significant changes in gene expression that are consistent with reduced NK cell and dendritic cell function in individual with SCI; compared to uninjured controls we observed decreased levels of perforin 1, IL2RB, killer cell lectin like receptors, and FLT3. We also discovered reduced frequencies of NK cells and plasmacytoid DCs, which are particularly critical for fighting viral infections, over a sustained period after SCI. These data extend previous observations showing decreased NK cell function and reduction of NK cell gene expression in individuals with chronic SCI(17,19–21,52). We also noted decreased expression of genes related to T cell function, including CD4, the transcription factors FOXP3 and TCF7, as well as reduced frequencies of major T cell subsets. In contrast, some genes related to T cell activation were upregulated, reflecting changes observed in T cell subpopulations after SCI.

Despite known differences in health outcomes in individuals with AIS A or B injuries at 12 MPI(37), here we identified a distinct gene expression profile that reflected injury severity (motor completeness) throughout the first year after SCI. Hierarchical clustering of DE genes in samples from individuals with injuries classified as motor complete or incomplete identified enrichment of genes related to activation of T cells, NF-κB signaling, Type I IFN production, and neutrophil function in individuals with motor complete injuries. In contrast, genes related to transcription, post-translational protein modifications, mitochondrial function, RHO-GTPases, and IL-6 signaling, among others, were downregulated in samples from participants with motor complete injuries. In addition to changes in gene expression, levels of circulating cytokines also varied by injury severity, with potent inflammatory cytokines such as HMGB1, MIF, and IFN-γ, higher in samples from motor complete injuries. Thus, highly complex changes in systemic gene expression appear to correlate grossly with the severity of motor and sensory impairment on the ISNCSCI exam, which lacks an assessment of the immune system or other physiological systems regulated by autonomic function. Data from this study supports the use of gene expression profiling in a precision medicine approach to inform clinical trials in SCI which test FDA-approved drugs or other types of therapies that target specific immune mediators which are over- or under-expressed relative to controls or are expressed according to injury severity.

This study faced limitations that are challenges in any natural history study after traumatic SCI: (1) challenges of conducting long-term follow up, particularly for geographical reasons since individuals may not live near where they received emergency care and follow-up treatment and traveling may be impractical/unfeasible, (2) inconsistency in sample acquisition at study visits for medical or practical reasons, (3) diversity in clinical and demographic characteristics such as level of injury, gender, ethnicity or age, (4) the blood samples were comprised of a mixed cell population, so future studies should utilize single-cell RNA-sequencing to assign gene expression changes to specific immune cell populations. In addition, a major limitation of this study is that it did not include a comparison to samples from individuals with non-neurological trauma, which could inform interpretation of the differences in immune profiles between individuals with and without SCI, but is less relevant to our comparison of immune profiles among individuals with SCI. For example, in one of the earliest trauma genome-wide molecular profiling studies, TLR signaling was upregulated, while TCR and IL-4 signaling pathways were downregulated, within the first 28 days of severe blunt trauma in humans, as we observe here after SCI (65). Lastly, while we would have liked to investigate blood-based biomarkers that are predictive of functional recovery during the first year after SCI (e.g., conversion from motor complete to incomplete on the ISNCSCI exam), we are limited in this small participant cohort by the lack of clinical conversion.

## Conclusions

Despite these limitations, to our knowledge, this study captures immunological changes after SCI in humans in unprecedented detail. This multidimensional analysis provides details of profound transcriptional and cellular immunological changes after SCI that demonstrate that the SCI-induced immune deficiency syndrome is dynamic and distinct in its acute and chronic states. It also demonstrates a neurogenic gene expression signature of SCI induced immunodeficiency. These include changes in frequencies of both innate and adaptive immune cell types, profound changes in gene expression, and elevated protein levels consistent with persistent inflammation. Some changes observed were specific to the first days after injury, while others were long-lasting, supporting the hypothesis that some immune modulating interventions may be effective at different times after SCI in reducing morbidity and mortality or serve as biomarkers of injury severity or time after injury. In the future, studies exploring functional changes in immune cells and their associations with infections and/or medical comorbidities would be extremely valuable.

## Supplementary Material

Supplemental data

Supplemental data

Supplemental data

Supplemental data

Supplemental data

## Transparency, Rigor, and Reproducibility Summary

This observational study was registered on Clinicialtrials.gov (NCT02731027). The analysis plan was not pre-registered. Key inclusion criteria and outcome measures are established standards in the field (time from injury, AIS grade, neurological level of injury). A sample size of 100 participants was assumed based on the average number of patients admitted to study sites and using a simplified power calculation for estimating Spearman correlations between AIS grade and a given biomarker level, a sample of 100 would have yielded 80% power to detect a correlation of 0.28 or greater (2-tailed alpha = 0.05, with no adjustment for multiple testing.) There were 1117 individuals assessed for eligibility. Of these, 33 declined to enroll, 47 were excluded due to medical reasons, and 998 did not meet inclusion/exclusion criteria. There were 42 participants who were enrolled, but one was withdrawn due to lesion level criteria ineligibility. Of the remaining 41, the following number completed these study visits with RNA-Seq samples that passed quality controls: V1 (N = 38, V2 (N = 14), V3 (N = 14), V4 (N = 8). In total, 12 participants were selected for this longitudinal analysis because they had sequential non-missing biological samples for study visits 1-3. Participants were not told and were unable to guess results of the fluid biomarkers measurements. Sample handling and quality control decisions were performed by personnel blinded to participant characteristics. De-identified participant IDs were used for all analyses. Equipment and analytical resources were from commercial suppliers described in methods. There were no analytic codes generated; all statistical analyses were performed in Prism GraphPad and Partek Genomics Flow, using algorithms and methods pre-specified by the commercial software. Biological samples used in this study were obtained by the investigators and are being used for additional analyses; limited quantities of some samples remain. We are not conducting replication studies. De-identified RNA-Seq data from this study are available in the GEO repository, accession GSE226238.

For single and multiplex ELISAs, samples were assayed in duplicate, and performed with standards according to manufactures' protocols. For singleplex ELISAs, avg. R^2^ values and %CVs were: CRP: R^2^ = 0.999, %CV = 6.5; HMGB1: (0-10 ng/ml), R^2^ = 0.998, %CV = 6.6. For multiplex ELISAs, the avg. lower limits of quantification (LLOQ, pg/ml) were: CTACK (0.948), Eotaxin (0.126), FGF basic (3.518), G-CSF (7.171), GROa (13.207), HGF (9.151), IFN-g (16.912), IL-13 (0.359), IL-16 (1.525), IL-18 (0.716), IL-1b (0.25), IL-1ra (4.211), IL-2Ra (1.358), IL-4 (0.145), IL-6 (0.326), IL-8 (0.535), IL-9 (2.311), IP-10 (2.516), M-CSF (0.751), MCP-1(0.473), MIF (3.477), MIG (4.794), MIP-1a (0.067), MIP-1b (0.668), PDGF-bb (6.515), RANTES (2.065), SCF (1.736), SCGF-b (76.926), SDF-1a (5.245), TNF-a (2.485), TNF-b (0.714), TRAIL (0.576).

The inter-assay %CVs for samples described here were: CTACK (5.5%), Eotaxin (5.7%), FGF basic (6.0%), G-CSF (5.7%), GROa (4.4%), HGF (4.5%), IFN-g (7.1%), IL-13 (6.1%), IL-16 (5.1%), IL-18 (5.1%), IL-1b (5.7%), IL-1ra (8.0%), IL-2Ra (5.5%), IL-4 (6.1%), IL-6 (4.8%), IL-8 (7.1%), IL-9 (6.1%), IP-10 (6.2%), M-CSF (5.3%), MCP-1 (5.8%), MIF (5.0%), MIG (6.1%), MIP-1a (5.8%), MIP-1b (6.4%), PDGF-bb (5.5%), RANTES (5.4%), SCF (5.1%), SCGF-b (5.0%), SDF-1a (5.0%), TNF-a (4.2%), TNF-b (8.1%), TRAIL (5.6%).
