# Supplementary material for: Profiling Immunological Phenotypes in Individuals During the First Year After Traumatic Spinal Cord Injury: A Longitudinal Analysis
Source: J Neurotrauma. 2023 Nov 30;40(23-24):2621–37. doi: 10.1089/neu.2022.0500 (PMC10722895; doi:10.1089/neu.2022.0500)
Supplement: Supplemental data [file Suppl_TableS1.docx]

| **Supplementary Table S1. Flow Cytometry Panels**  Panel: NK, Monocytes and Dendritic Cells | | | |
| --- | --- | --- | --- |
| Antigen | Fluorescent Tag | Manufacturer | Catalogue Number |
| CD56 | PE | BD Biosciences | 556647 |
| CD123 | PerCP-Cy5.5 | BD Biosciences | 558714 |
| CD11c | PE-Cy7 | BD Biosciences | 561356 |
| CD16 | APC | BD Biosciences | 561304 |
| CD3 | APC-Cy7 | BD Biosciences | 557757 |
| CD14 | Alexa700 | BD Biosciences | 557923 |
| HLA-DR | FITC | Miltenyi Biotec | 13011340 |
| Panel: T Cells | | | |
| Antigen | Fluorescent Tag | Manufacturer | Catalogue Number |
| CD25 | PE | BD Biosciences | 557138 |
| CD4 | PerCP-Cy5.5 | BD Biosciences | 560650 |
| CCR4 | PE-CY7 | BioLegend | 359410 |
| CD127 | APC | BioLegend | 351316 |
| CD3 | APC-H7 | BD Biosciences | 641397 |
| CD8 | Alexa700 | BD Biosciences | 557945 |
| HLA-DR | FITC | Miltenyi Biotec | 130113401 |
| Panel: B Cells | | | |
| Antigen | Fluorescent Tag | Manufacturer | Catalogue Number |
| CD24 | PE | BD Biosciences | 560991 |
| CD19 | PerCP-Cy5.5 | BD Biosciences | 561295 |
| CD27 | PE-Cy7 | BD Biosciences | 560609 |
| CD38 | APC | BD Biosciences | 555462 |
| CD20 | APC-Cy7 | BD Biosciences | 335794 |
| CD3 | Alexa700 | BD Biosciences | 557493 |
| IgD | FITC | BD Biosciences | 562023 |
