# Supplementary material for: Profiling Immunological Phenotypes in Individuals During the First Year After Traumatic Spinal Cord Injury: A Longitudinal Analysis
Source: J Neurotrauma. 2023 Nov 30;40(23-24):2621–37. doi: 10.1089/neu.2022.0500 (PMC10722895; doi:10.1089/neu.2022.0500)

**SUPPLEMENTARY FIG. S1.** Flow cytometry pseudocolor plots illustrate the gating strategies. **(A)** CD56^dim^ (CD56^+^CD16^++^) and CD56^bright^ (CD56^++^CD16^-/+^) natural killer (NK) cells (linCD3^-^CD14^-^). **(B)** Classical (CD14^++^CD16^-^), intermediate (CD14^++^CD16^+^), and non-classical (CD14^+/-^CD16^++)^ monocytes (linCD3^-^CD56^-^) and plasmacytoid dendritic cells (CD3^-^CD56^-^CD14^-^CD16^-^HLA-DR^+^CD11c^-^CD123^+^). **(C)** Activated (HLA-DR^+^) CD8^+^ and CD4^+^ T-cell subsets (linCD3^+^).


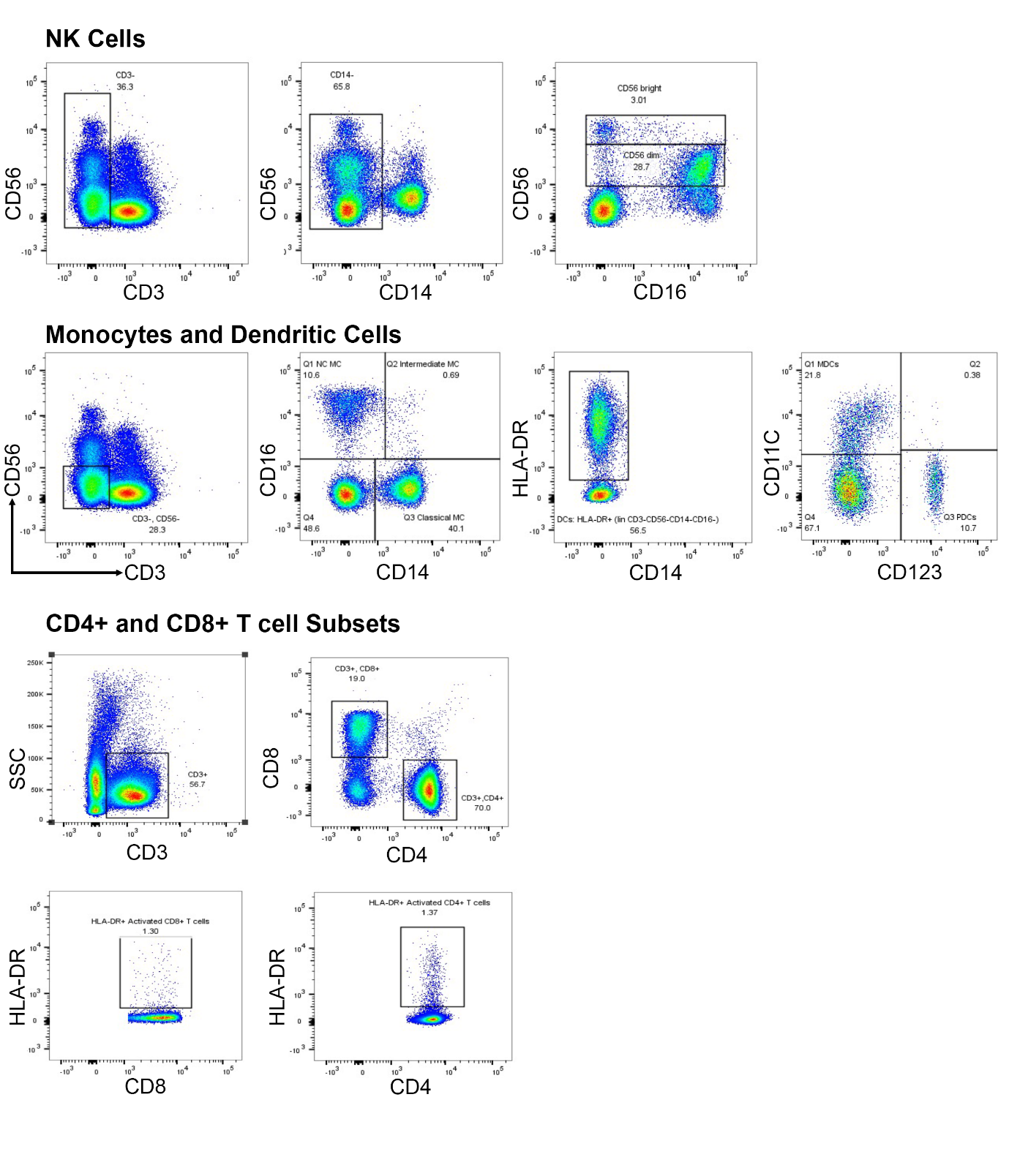

Supplement: Supplemental data [file Suppl_FigureS1.docx]
