# Supplementary material for: Profiling Immunological Phenotypes in Individuals During the First Year After Traumatic Spinal Cord Injury: A Longitudinal Analysis
Source: J Neurotrauma. 2023 Nov 30;40(23-24):2621–37. doi: 10.1089/neu.2022.0500 (PMC10722895; doi:10.1089/neu.2022.0500)
Supplement: Supplemental data [file Suppl_TableS2.pdf]

Supplementary Table S2: Figure 1B gene list				
**Gene symbol**	**Transcript ID**	**Total counts**	**P-value**	**FDR step up**
ABCC1	NM_004996	1.16E+03	5.33E-06	2.63E-04
ABCC2	NM_000392	1.32E+02	8.92E-06	3.80E-04
ABHD5	NM_001365649	8.14E+01	2.45E-05	7.47E-04
ABLIM1	NM_001322896	3.26E+02	5.60E-06	2.74E-04
ACACB	NM_001093	3.80E+02	5.58E-06	2.74E-04
ACBD6	NM_032360	1.98E+02	6.26E-06	2.97E-04
ACER3	NM_018367	4.80E+02	3.47E-05	9.70E-04
ACOT4	NM_152331	3.59E+01	3.21E-07	3.65E-05
ACOX1	NM_001185039	5.32E+02	1.48E-05	5.39E-04
ACSL1	NM_001995	2.83E+04	3.99E-07	4.27E-05
ACSL4	NM_004458	9.43E+02	8.88E-06	3.79E-04
ADAM17	NM_003183	1.72E+03	6.00E-10	6.30E-07
ADAM9	NM_003816	4.98E+02	5.04E-10	6.30E-07
ADAMTS17	NM_139057	3.10E+01	7.81E-08	1.42E-05
ADAMTS2	NM_014244	2.38E+02	9.13E-18	3.05E-13
ADAP2	NM_018404	7.37E+02	3.73E-07	4.05E-05
ADGRE2	NM_001271052	5.50E+03	1.35E-05	5.03E-04
ADGRE4P	NR_024075	3.73E+02	7.74E-07	6.81E-05
ADPGK	NM_031284	1.18E+03	1.01E-05	4.17E-04
ADPRH	NM_001291950	2.28E+02	1.24E-05	4.77E-04
AFAP1	NM_198595	7.36E+01	1.36E-05	5.06E-04
AGFG1	NM_004504	2.08E+03	3.42E-09	2.08E-06
AGK	NM_018238	3.58E+02	2.71E-07	3.28E-05
AGMAT	NM_024758	1.12E+02	3.19E-06	1.83E-04
AIM2	NM_004833	3.66E+02	3.46E-05	9.67E-04
AK5	NM_174858	1.23E+02	1.15E-09	9.66E-07
AKAP1	NM_001370426	5.75E+01	3.35E-05	9.44E-04
ALDH1B1	NM_000692	1.20E+02	1.77E-05	5.98E-04
ALG13	NM_018466	3.87E+01	1.93E-05	6.36E-04
ALOX15	NM_001140	3.62E+02	1.82E-08	5.98E-06
ALPK1	NM_001253884	1.41E+03	1.14E-05	4.51E-04
AMIGO1	NM_020703	5.24E+02	3.24E-09	2.08E-06
AMPH	NM_139316	5.90E+01	5.50E-09	2.78E-06
ANAPC1	NM_022662	7.57E+02	1.04E-05	4.25E-04
ANKH	NM_054027	1.00E+03	1.36E-05	5.05E-04
ANKS6	NM_173551	2.00E+02	1.82E-07	2.51E-05

ANXA1	NM_000700	7.42E+03	3.12E-05	8.97E-04
ANXA3	NM_005139	2.63E+03	5.77E-07	5.36E-05
AOAH	NM_001177506	1.09E+03	1.64E-05	5.68E-04
AOAH-IT1	NR_046764	2.21E+01	1.28E-06	9.61E-05
AP3S1	NM_001284	6.82E+02	1.39E-07	2.14E-05
APH1B	NM_031301	9.22E+02	7.67E-07	6.76E-05
APOBEC3D	NM_152426	5.13E+02	2.45E-05	7.46E-04
AQP3	NM_004925	7.62E+02	4.40E-07	4.51E-05
AQP9	NM_001320636	7.93E+03	1.98E-05	6.45E-04
ARF4	NM_001660	2.17E+03	4.38E-06	2.33E-04
ARG1	NM_000045	7.56E+02	5.84E-08	1.20E-05
ARHGAP10	NM_024605	1.27E+02	1.04E-07	1.76E-05
ARHGAP26	NM_001349547	3.51E+03	3.19E-05	9.11E-04
ARHGDIB	NM_001321421	7.20E+02	1.04E-08	4.34E-06
ARHGEF40	NM_001278530	5.88E+02	1.17E-06	9.02E-05
ARL11	NM_138450	1.46E+03	4.41E-06	2.33E-04
ARL4C	NM_005737	3.21E+03	5.31E-08	1.15E-05
ARMH2	NM_001282492	4.77E+01	1.15E-05	4.55E-04
ARPC2	NM_005731	6.00E+03	1.96E-05	6.40E-04
ARPC3	NM_001278556	7.93E+03	9.75E-07	7.93E-05
ARPC5	NM_001270439	6.43E+03	9.34E-06	3.94E-04
ARRB2	NM_199004	1.27E+03	1.04E-05	4.24E-04
ASAH1	NM_177924	4.86E+03	3.05E-06	1.78E-04
ASB13	NR_024581	2.12E+02	2.48E-05	7.54E-04
ASB2	NM_016150	9.86E+01	2.17E-07	2.83E-05
ASB7	NM_198243	7.13E+02	2.93E-05	8.54E-04
ASPH	NM_004318	2.27E+02	4.52E-07	4.56E-05
ATF6	NM_007348	3.44E+03	2.27E-05	7.13E-04
ATG9B	NR_073169	1.24E+02	2.93E-07	3.44E-05
ATP10A	NM_024490	3.95E+02	5.86E-08	1.20E-05
ATP11B	NM_014616	3.42E+03	3.42E-09	2.08E-06
ATP6V0E1	NM_003945	8.76E+03	1.22E-05	4.72E-04
ATP6V1C1	NM_001695	1.96E+03	1.10E-08	4.45E-06
ATP6V1D	NM_015994	9.93E+02	1.63E-06	1.14E-04
ATP8B2	NM_020452	9.51E+02	6.45E-09	3.09E-06
ATXN1	NM_001128164	9.66E+02	1.48E-06	1.06E-04
ATXN2	NM_002973	4.34E+01	1.24E-05	4.77E-04
AXIN2	NM_004655	6.26E+01	1.55E-09	1.21E-06

B4GALT5	NM_004776	6.03E+03	6.29E-07	5.71E-05
B4GAT1	NM_006876	8.48E+01	3.34E-11	1.40E-07
BACE2	NM_012105	1.56E+02	6.16E-07	5.65E-05
BAG3	NM_004281	1.93E+02	1.37E-05	5.08E-04
BASP1	NM_001271606	4.59E+03	3.10E-05	8.94E-04
BASP1-AS1	NR_027253	3.44E+01	5.35E-06	2.64E-04
BCAM	NM_005581	1.46E+01	2.08E-05	6.65E-04
BCAT1	NM_001178093	1.38E+02	7.22E-08	1.36E-05
BCKDHB	NM_000056	2.52E+01	2.54E-05	7.63E-04
BCL10	NM_003921	9.00E+02	2.35E-06	1.49E-04
BCL11B	NM_001282237	2.12E+02	7.19E-11	1.72E-07
BCL2	NM_000633	1.33E+03	4.54E-07	4.56E-05
BCL2A1	NM_004049	1.06E+03	3.57E-05	9.89E-04
BCL6	NM_001130845	1.34E+04	1.40E-07	2.14E-05
BCL9L	NM_182557	2.20E+03	1.42E-05	5.20E-04
BDH1	NM_004051	1.18E+02	1.18E-08	4.60E-06
BEST1	NM_001300786	1.05E+02	4.35E-06	2.31E-04
BICDL1	NR_147894	5.54E+01	1.08E-06	8.49E-05
BLOC1S2	NM_001001342	8.98E+01	2.85E-05	8.38E-04
BLOC1S6	NR_132359	3.37E+02	1.08E-05	4.37E-04
BORCS5	NM_001300742	1.07E+02	3.13E-06	1.81E-04
BROX	NM_001288580	5.73E+02	1.69E-05	5.78E-04
BST1	NM_004334	3.24E+03	2.72E-06	1.64E-04
BTBD10	NM_001297741	6.95E+02	3.16E-06	1.82E-04
BTBD11	NM_001018072	4.17E+01	2.34E-05	7.28E-04
C12orf65	NM_152269	2.87E+02	4.94E-07	4.83E-05
C12orf75	NM_001145199	3.03E+02	5.13E-07	4.95E-05
C16orf70	NM_001320540	2.70E+02	2.40E-05	7.39E-04
C16orf72	NM_014117	3.09E+03	1.09E-06	8.54E-05
C1GALT1C1	NM_001011551	5.04E+02	8.27E-06	3.62E-04
C1orf162	NM_001300834	3.35E+03	1.09E-06	8.54E-05
C1RL	NM_001297640	1.19E+03	2.77E-05	8.22E-04
C22orf46	NR_160905	3.77E+02	1.93E-06	1.29E-04
C3AR1	NM_004054	1.55E+03	7.50E-09	3.48E-06
C3orf62	NM_198562	2.14E+03	5.80E-08	1.20E-05
C4orf3	NM_001001701	3.41E+03	5.26E-06	2.62E-04
C5orf67	NR_161255	3.64E+01	1.88E-05	6.23E-04
C9orf152	NM_001012993	9.77E+00	9.61E-06	4.03E-04

C9orf72	NM_018325	1.63E+03	2.63E-05	7.88E-04
CA12	NM_001293642	1.12E+01	2.81E-05	8.29E-04
CA4	NR_137422	1.08E+02	7.99E-06	3.56E-04
CAB39	NM_001130849	1.77E+02	6.19E-07	5.67E-05
CABP4	NM_145200	2.50E+01	2.76E-05	8.18E-04
CACNG8	NM_031895	1.08E+02	2.29E-07	2.92E-05
CALM2	NM_001743	9.65E+03	9.44E-06	3.97E-04
CAMKK2	NM_001270486	4.57E+03	3.04E-08	8.06E-06
CAP1	NM_001105530	3.27E+03	1.44E-05	5.28E-04
CAPN2	NM_001146068	2.59E+03	3.37E-05	9.48E-04
CAPZA1	NM_006135	1.02E+04	4.68E-06	2.44E-04
CAPZA2	NM_006136	2.90E+03	1.82E-05	6.10E-04
CARD11	NM_001324281	5.24E+02	1.64E-05	5.68E-04
CARD6	NM_032587	1.63E+03	1.22E-06	9.23E-05
CASP4	NM_001225	6.25E+03	1.17E-05	4.61E-04
CBX5	NM_012117	7.02E+02	8.11E-06	3.59E-04
CCDC71L	NM_175884	7.29E+02	1.72E-05	5.86E-04
CCDC85C	NM_001144995	1.36E+02	1.61E-05	5.67E-04
CCDC88C	NM_001080414	2.63E+03	3.47E-06	1.96E-04
CCL28	NM_001301873	4.19E+01	7.28E-08	1.37E-05
CCND2	NM_001759	2.08E+03	2.98E-08	8.03E-06
CCNDBP1	NM_012142	5.13E+03	1.49E-05	5.39E-04
CCPG1	NM_020739	2.24E+03	2.23E-06	1.43E-04
CCR2	NM_001123396	4.96E+03	1.32E-07	2.06E-05
CCR3	NM_178329	1.13E+03	1.44E-07	2.18E-05
CCR4	NM_005508	6.52E+02	4.29E-07	4.43E-05
CCR7	NM_001838	2.97E+03	3.47E-06	1.96E-04
CCZ1	NM_015622	6.90E+02	8.19E-06	3.60E-04
CD163	NM_004244	1.39E+03	4.65E-08	1.05E-05
CD177	NM_020406	4.75E+03	3.10E-07	3.55E-05
CD1D	NM_001319145	7.87E+02	1.72E-06	1.19E-04
CD2	NM_001767	3.83E+03	7.55E-08	1.40E-05
CD247	NM_000734	2.17E+03	1.94E-07	2.64E-05
CD28	NM_006139	1.41E+03	2.00E-07	2.71E-05
CD3E	NM_000733	5.39E+03	5.16E-09	2.77E-06
CD3G	NM_000073	5.66E+02	1.50E-07	2.22E-05
CD40LG	NM_000074	6.61E+02	1.83E-08	5.98E-06
CD44	NM_001202555	1.52E+03	1.24E-06	9.33E-05

CD5	NM_014207	1.78E+03	3.75E-08	9.56E-06
CD53	NM_001040033	3.40E+03	5.39E-10	6.30E-07
CD55	NM_001114752	5.17E+02	6.47E-09	3.09E-06
CD83	NM_004233	1.20E+02	5.76E-06	2.80E-04
CD96	NM_005816	1.38E+03	6.99E-08	1.34E-05
CDC42	NM_001791	9.01E+03	2.53E-06	1.55E-04
CDC42BPG	NM_017525	1.03E+02	1.91E-06	1.28E-04
CDC42EP3	NM_001270436	1.24E+03	6.59E-10	6.30E-07
CDC7	NM_001134419	5.35E+01	5.30E-06	2.63E-04
CDKN1C	NM_001122630	6.30E+01	5.60E-06	2.74E-04
CDKN2B	NM_004936	8.92E+01	3.78E-08	9.56E-06
CDR2	NM_001802	6.89E+02	3.91E-09	2.34E-06
CEACAM21	NM_001288773	1.91E+02	4.19E-06	2.24E-04
CEP170B	NM_015005	2.68E+01	1.36E-06	9.99E-05
CEP19	NM_032898	1.21E+03	8.02E-06	3.56E-04
CEPT1	NM_006090	4.76E+02	1.81E-05	6.08E-04
CERS2	NM_022075	4.52E+03	3.77E-06	2.09E-04
CERT1	NM_031361	5.31E+02	2.93E-05	8.54E-04
CFLAR	NR_147242	3.98E+02	2.09E-06	1.37E-04
CGAS	NM_138441	3.06E+02	2.51E-05	7.55E-04
CHI3L2	NM_001025199	3.10E+02	8.88E-06	3.79E-04
CHIC2	NM_012110	7.47E+02	1.03E-05	4.24E-04
CHMP3	NM_016079	2.65E+03	3.04E-06	1.78E-04
CHMP7	NM_152272	9.17E+02	1.97E-08	6.24E-06
CHST12	NM_018641	2.34E+02	4.95E-06	2.51E-04
CHURC1-FNTB	NM_001202559	7.22E+01	7.93E-06	3.54E-04
CKAP4	NM_006825	3.49E+03	1.18E-10	2.46E-07
CLEC4D	NM_080387	5.46E+02	9.52E-08	1.65E-05
CLRN1-AS1	NR_024066	2.18E+01	2.16E-07	2.83E-05
CLTC	NM_001288653	9.31E+02	1.59E-05	5.62E-04
CLUHP3	NR_024034	1.39E+02	2.50E-05	7.55E-04
CMIP	NM_030629	8.18E+02	1.63E-05	5.68E-04
CMTM6	NM_017801	1.19E+04	1.94E-05	6.37E-04
CNBP	NM_001127196	2.22E+03	2.28E-05	7.15E-04
CNIH4	NM_014184	6.75E+02	4.16E-06	2.24E-04
CNR2	NM_001841	5.27E+02	8.47E-07	7.28E-05
CNTNAP3	NM_033655	8.71E+02	1.34E-06	9.89E-05
CNTNAP3P2	NR_111893	4.80E+02	1.50E-06	1.07E-04

CORO1C	NM_001105237	5.37E+02	6.53E-06	3.06E-04
CPD	NM_001199775	5.37E+03	8.68E-06	3.75E-04
CPM	NM_198320	1.35E+02	2.34E-06	1.49E-04
CPQ	NM_016134	2.59E+03	5.00E-06	2.52E-04
CPSF7	NM_001136040	1.87E+03	2.16E-07	2.83E-05
CR1	NM_000573	5.15E+03	1.93E-09	1.43E-06
CRADD	NM_003805	1.55E+02	8.35E-06	3.64E-04
CREG1	NM_003851	2.53E+03	1.75E-06	1.20E-04
CRIP2	NM_001312	1.04E+02	5.94E-06	2.86E-04
CRISPLD2	NM_031476	3.27E+03	2.36E-05	7.33E-04
CRK	NM_005206	8.33E+02	1.39E-05	5.12E-04
CRTC3	NM_001042574	5.61E+02	1.08E-05	4.35E-04
CRY2	NM_021117	5.51E+02	2.03E-05	6.56E-04
CSGALNACT2	NM_018590	1.58E+03	1.08E-06	8.51E-05
CTSF	NM_003793	1.55E+02	2.10E-05	6.71E-04
CUX1	NM_181552	6.06E+02	2.37E-05	7.34E-04
CWC25	NM_017748	1.36E+03	3.26E-05	9.27E-04
CYB561	NM_001915	3.92E+02	9.25E-07	7.69E-05
CYB5R4	NM_016230	2.55E+03	8.28E-06	3.62E-04
CYP19A1	NM_001347249	3.22E+00	5.93E-10	6.30E-07
CYP1B1	NM_000104	1.81E+03	4.19E-09	2.37E-06
CYP1B1-AS1	NR_027252	1.24E+02	2.37E-08	6.76E-06
CZ1P-ASNS	NR_147989	1.40E+02	3.21E-06	1.84E-04
DBH-AS1	NR_102735	7.87E+01	1.95E-05	6.38E-04
DCAF4L1	NM_001029955	6.29E+01	2.38E-06	1.49E-04
DDHD2	NM_015214	5.39E+01	2.08E-05	6.67E-04
DDX59	NM_001031725	2.24E+02	2.43E-05	7.45E-04
DENND2D	NM_024901	3.30E+03	1.58E-07	2.29E-05
DERA	NM_015954	3.86E+02	9.57E-07	7.82E-05
DGCR8	NM_022720	6.14E+02	1.22E-05	4.72E-04
DGKA	NM_001345	2.64E+03	5.99E-06	2.87E-04
DGKE	NM_003647	4.23E+02	1.12E-05	4.44E-04
DHRS3	NM_004753	1.72E+02	1.21E-08	4.64E-06
DHRS7	NM_016029	3.40E+03	2.84E-05	8.37E-04
DIP2C	NM_014974	6.16E+01	2.68E-06	1.62E-04
DLG3	NM_001166278	2.37E+02	2.71E-08	7.49E-06
DLL1	NM_005618	2.11E+01	3.61E-05	9.97E-04
DNAJA2	NM_005880	1.47E+03	1.40E-06	1.02E-04

DNAJC3	NM_006260	2.51E+03	2.36E-08	6.76E-06
DNAJC3-DT	NR_132117	1.27E+02	5.34E-07	5.10E-05
DNMT1	NM_001130823	2.48E+02	4.93E-06	2.51E-04
DOCK9-DT	NR_047482	3.31E+01	4.84E-06	2.48E-04
DPP4	NM_001935	5.70E+02	1.36E-07	2.10E-05
DPY19L3	NM_001172774	4.38E+02	5.02E-06	2.53E-04
DPYSL4	NM_006426	2.33E+01	1.32E-06	9.79E-05
DRAM1	NM_018370	9.05E+02	4.54E-11	1.52E-07
DSE	NM_001080976	1.51E+02	1.63E-05	5.68E-04
DTX1	NM_004416	1.69E+02	1.35E-05	5.04E-04
DUSP1	NM_004417	8.17E+03	1.73E-07	2.45E-05
DUSP14	NM_007026	8.01E+01	2.42E-06	1.50E-04
DUSP22	NM_001286555	9.04E+02	2.45E-05	7.46E-04
DYRK1A	NM_001347722	1.59E+02	6.10E-06	2.92E-04
DYSF	NM_001130986	8.59E+02	1.08E-05	4.35E-04
EARS2	NR_003501	1.52E+02	2.90E-05	8.47E-04
ECHDC2	NM_001319958	2.84E+02	1.82E-05	6.10E-04
ECRP	NR_033909	7.55E+01	7.98E-07	6.95E-05
EEF2K	NM_013302	5.23E+02	4.45E-11	1.52E-07
EIF1B-AS1	NR_033965	3.42E+01	2.01E-07	2.72E-05
EIF4E3	NM_001134649	9.19E+02	3.58E-08	9.22E-06
ELMO2	NM_001318253	4.55E+02	2.42E-05	7.42E-04
EMC7	NM_020154	8.82E+02	3.80E-06	2.10E-04
ENTPD1	NM_001320916	9.04E+02	1.05E-06	8.33E-05
EP400	NM_015409	1.25E+03	1.49E-05	5.39E-04
EPHA1	NM_005232	2.03E+02	3.17E-05	9.10E-04
ERG28	NM_007176	3.61E+02	1.52E-05	5.47E-04
ERLIN1	NM_001347856	1.37E+02	3.80E-06	2.10E-04
ERLIN2	NM_007175	7.08E+02	9.57E-07	7.82E-05
ESYT1	NM_015292	2.66E+03	2.96E-07	3.44E-05
ETS2	NM_005239	2.05E+03	1.88E-08	6.09E-06
EVI2B	NM_006495	4.00E+04	8.86E-06	3.79E-04
EVL	NM_016337	4.21E+03	2.67E-07	3.26E-05
EXT1	NM_000127	4.18E+02	3.06E-07	3.51E-05
F5	NM_000130	2.11E+03	6.53E-06	3.06E-04
FAM102A	NM_001035254	1.43E+03	1.19E-07	1.92E-05
FAM107B	NM_001320740	1.00E+03	1.08E-05	4.35E-04
FAM171A1	NM_001010924	1.12E+02	2.28E-08	6.62E-06

FAM200B	NM_001145191	6.15E+02	2.07E-05	6.64E-04
FAM20A	NR_027751	7.80E+01	2.39E-06	1.49E-04
FAM241B	NM_145306	3.68E+01	2.01E-05	6.51E-04
FAM49B	NM_001353270	4.44E+01	1.03E-06	8.24E-05
FAM53C	NM_001350194	3.58E+03	2.12E-05	6.72E-04
FAN1	NM_014967	5.41E+02	3.44E-06	1.95E-04
FAR1	NM_032228	2.56E+03	1.18E-05	4.61E-04
FAR2	NM_001271783	1.13E+03	4.61E-08	1.05E-05
FBP1	NM_001127628	1.21E+02	1.98E-05	6.44E-04
FBXL16	NM_153350	2.28E+02	4.50E-07	4.56E-05
FBXL5	NR_036464	4.42E+02	1.08E-05	4.35E-04
FBXW2	NM_012164	2.94E+03	2.12E-05	6.73E-04
FCAR	NM_002000	2.07E+03	1.68E-06	1.16E-04
FCGR2A	NM_001136219	2.04E+04	2.40E-06	1.49E-04
FCMR	NM_001142473	3.02E+02	4.47E-08	1.04E-05
FCRL3	NM_052939	1.11E+03	3.47E-07	3.85E-05
FEN1	NM_004111	2.32E+02	4.82E-07	4.75E-05
FGD4	NM_001330374	2.48E+02	2.87E-05	8.42E-04
FGF13	NM_004114	1.25E+01	9.21E-06	3.90E-04
FGFRL1	NM_001004358	8.10E+01	2.89E-05	8.45E-04
FGR	NM_001042747	1.62E+04	1.56E-05	5.55E-04
FKBP15	NM_015258	4.22E+03	1.82E-07	2.51E-05
FKBP5	NM_001145775	4.30E+02	4.11E-07	4.33E-05
FLNB	NM_001164319	2.82E+02	2.06E-05	6.63E-04
FLT3	NM_004119	3.69E+02	9.83E-07	7.98E-05
FLYWCH1	NM_020912	3.67E+01	8.77E-06	3.76E-04
FMNL3	NM_198900	1.03E+03	7.95E-10	7.16E-07
FNIP1	NM_001346114	4.52E+02	7.99E-07	6.95E-05
FOXP3	NM_014009	5.82E+01	2.17E-07	2.83E-05
FPR1	NM_001193306	3.94E+03	3.30E-05	9.34E-04
FUCA2	NM_032020	7.66E+02	9.42E-06	3.97E-04
FZD5	NM_003468	3.84E+01	1.63E-05	5.68E-04
GABARAPL2	NM_007285	3.07E+03	1.63E-05	5.68E-04
GABRR2	NM_002043	2.04E+02	4.99E-06	2.52E-04
GALNT12	NM_024642	1.11E+02	7.12E-06	3.25E-04
GALNT3	NM_004482	7.29E+02	5.83E-07	5.40E-05
GAREM2	NM_001168241	5.79E+01	4.18E-08	1.01E-05
GAS7	NM_001130831	1.54E+03	5.78E-08	1.20E-05

GASK1B	NM_001128424	5.63E+02	2.96E-07	3.44E-05
GATA3	NM_001002295	3.22E+02	4.05E-08	1.00E-05
GCA	NM_012198	1.29E+04	5.26E-06	2.62E-04
GCLM	NM_002061	3.04E+02	1.06E-07	1.78E-05
GCNA	NM_052957	1.51E+02	1.46E-05	5.33E-04
GDE1	NM_016641	1.74E+03	2.40E-07	3.02E-05
GDF11	NM_005811	3.54E+02	1.84E-07	2.51E-05
GFI1	NM_001127216	3.08E+02	5.09E-06	2.56E-04
GGT5	NM_004121	7.84E+00	4.29E-06	2.29E-04
GHITM	NM_014394	5.02E+03	4.46E-06	2.34E-04
GIPR	NM_000164	4.98E+01	6.25E-06	2.97E-04
GIT2	NM_139201	1.47E+03	5.09E-06	2.56E-04
GLIPR1	NM_006851	8.50E+03	8.73E-07	7.41E-05
GLT1D1	NM_001366887	1.45E+03	1.28E-05	4.86E-04
GM2A	NM_000405	1.80E+03	3.57E-05	9.89E-04
GMPR2	NM_001351026	1.14E+02	4.09E-07	4.32E-05
GNAI3	NM_006496	3.33E+03	5.86E-06	2.84E-04
GNG10	NM_001017998	2.28E+03	1.83E-05	6.13E-04
GNG5	NM_005274	2.52E+03	3.48E-05	9.71E-04
GNS	NM_002076	7.27E+03	6.94E-08	1.33E-05
GOLGA2P5	NR_024261	9.03E+01	3.88E-06	2.12E-04
GOLGA7	NM_001174124	2.11E+02	1.69E-06	1.17E-04
GOLGA7B	NM_001010917	4.50E+02	4.85E-06	2.48E-04
GPA33	NM_005814	1.47E+02	8.96E-09	3.94E-06
GPAT3	NM_001256421	7.98E+02	1.65E-06	1.15E-04
GPBP1L1	NM_021639	3.68E+03	7.99E-06	3.56E-04
GPR160	NM_014373	4.85E+02	1.48E-05	5.38E-04
GPR19	NM_006143	5.11E+01	3.62E-05	9.99E-04
GPR27	NM_018971	9.49E+02	1.91E-06	1.28E-04
GRAP2	NM_004810	1.14E+03	6.16E-10	6.30E-07
GRB10	NM_001350815	1.07E+01	1.27E-07	2.01E-05
GSR	NM_000637	1.81E+03	5.19E-08	1.15E-05
GYG1	NM_001184720	2.68E+03	6.24E-11	1.72E-07
H2AFY	NM_004893	1.67E+03	6.11E-07	5.62E-05
H3F3B	NM_005324	2.55E+04	2.31E-05	7.21E-04
HABP4	NM_014282	3.44E+02	8.31E-11	1.85E-07
HACD4	NM_001321903	1.23E+03	1.37E-05	5.08E-04
HADHB	NM_000183	2.26E+03	1.10E-06	8.54E-05

HAL	NM_002108	2.01E+03	7.06E-06	3.23E-04
HAUS4	NM_017815	8.66E+02	4.54E-07	4.56E-05
HAUS5	NM_015302	2.93E+02	4.48E-08	1.04E-05
HDAC4	NM_006037	1.80E+03	1.17E-07	1.90E-05
HEBP2	NM_001326380	8.94E+02	2.24E-05	7.04E-04
HEMK1	NM_016173	5.06E+02	2.03E-05	6.56E-04
HEXB	NM_000521	2.32E+03	3.63E-06	2.03E-04
HGF	NM_001010932	7.11E+01	1.04E-05	4.24E-04
HHEX	NM_002729	1.62E+03	1.54E-07	2.26E-05
HIC1	NM_006497	4.04E+01	2.98E-05	8.67E-04
HIPK1	NM_181358	1.91E+03	4.61E-07	4.60E-05
HIST2H2BE	NM_003528	1.86E+03	7.42E-08	1.38E-05
HKDC1	NM_025130	1.06E+02	3.42E-07	3.83E-05
HMGB2	NM_001130688	5.01E+02	1.47E-07	2.19E-05
HNMT	NM_006895	4.40E+02	3.29E-05	9.32E-04
HNRNPC	NM_004500	5.23E+03	5.23E-07	5.03E-05
HOPX	NM_139212	7.59E+00	7.52E-07	6.65E-05
HPCAL4	NM_001282396	2.82E+01	3.74E-06	2.08E-04
HPGD	NM_000860	2.00E+02	2.45E-05	7.47E-04
HSD17B11	NM_016245	6.65E+03	4.73E-06	2.44E-04
HSDL2	NM_032303	1.75E+03	2.92E-06	1.74E-04
HTATIP2	NM_006410	1.20E+03	4.19E-07	4.35E-05
HTRA1	NM_002775	6.91E+01	3.15E-06	1.82E-04
ID3	NM_002167	2.03E+02	1.01E-05	4.16E-04
IDH1	NM_005896	1.29E+03	2.42E-08	6.80E-06
IDO1	NM_002164	2.34E+02	1.10E-06	8.54E-05
IFFO2	NM_001136265	8.80E+02	1.05E-06	8.33E-05
IFNGR1	NM_000416	9.82E+03	1.36E-08	4.94E-06
IFNGR2	NM_005534	7.73E+03	9.46E-08	1.65E-05
IFRD1	NM_001197080	6.73E+02	2.88E-06	1.72E-04
IFT172	NM_015662	1.61E+02	1.18E-06	9.02E-05
IGFBP7	NM_001553	2.89E+02	2.47E-05	7.52E-04
IKZF3	NM_001257412	3.88E+02	1.29E-08	4.83E-06
IL10	NM_000572	3.04E+01	8.38E-06	3.64E-04
IL10RB	NM_000628	5.73E+03	5.22E-09	2.77E-06
IL11RA	NR_052010	1.26E+02	1.16E-07	1.89E-05
IL18	NM_001562	1.01E+02	4.83E-06	2.48E-04
IL18R1	NM_001282399	4.02E+02	2.45E-06	1.51E-04

IL18RAP	NM_003853	4.99E+03	6.08E-08	1.23E-05
IL1R1	NM_001288706	2.73E+01	1.58E-06	1.12E-04
IL1R2	NM_004633	5.49E+03	1.60E-06	1.13E-04
IL2RB	NM_001346223	7.35E+02	1.35E-06	9.94E-05
IL32	NM_001012718	2.97E+02	1.38E-06	1.01E-04
IL4R	NM_001257407	4.63E+02	2.48E-05	7.54E-04
IL5RA	NM_175727	3.05E+01	1.27E-05	4.83E-04
IL7R	NR_120485	4.13E+03	2.78E-05	8.22E-04
ING5	NM_032329	4.04E+02	2.59E-07	3.19E-05
INSC	NM_001278315	1.26E+01	6.98E-06	3.20E-04
INSIG1	NM_001346591	2.17E+01	4.49E-06	2.35E-04
INTS6	NM_001039937	8.57E+02	1.11E-05	4.43E-04
IPO11-LRRC70	NR_073584	1.45E+02	1.99E-08	6.24E-06
IPO9	NM_018085	1.31E+03	1.80E-05	6.06E-04
IQGAP1	NM_003870	2.03E+04	2.89E-05	8.45E-04
IQSEC2	NM_001111125	5.07E+01	1.52E-05	5.46E-04
IRAK3	NM_001142523	4.24E+03	4.12E-09	2.37E-06
IRAK4	NM_001351342	3.51E+02	3.09E-05	8.91E-04
IRF5	NM_001242452	7.19E+02	1.10E-05	4.40E-04
IRF8	NM_001363908	5.55E+02	6.83E-06	3.16E-04
ISM1	NM_080826	2.70E+01	2.24E-06	1.44E-04
ITGAM	NM_001145808	3.29E+03	1.40E-05	5.14E-04
ITK	NM_005546	3.10E+03	3.03E-07	3.50E-05
ITPKB	NM_002221	3.54E+03	1.16E-05	4.57E-04
ITPRID2	NM_001130445	1.16E+03	1.21E-05	4.71E-04
ITPRIP	NM_033397	2.36E+02	1.34E-05	5.02E-04
ITPRIPL1	NM_178495	1.88E+02	5.94E-06	2.86E-04
ITPRIPL2	NR_028028	5.34E+02	4.86E-06	2.48E-04
JPT2	NM_144570	7.46E+02	1.11E-05	4.44E-04
KANSL3	NR_047656	2.00E+02	1.22E-05	4.72E-04
KAT14	NM_020536	4.29E+02	2.50E-05	7.55E-04
KBTBD7	NM_032138	6.81E+02	1.70E-05	5.81E-04
KCMF1	NM_020122	1.95E+03	5.74E-06	2.80E-04
KCNA3	NM_002232	1.87E+02	4.37E-07	4.50E-05
KCNA6	NM_002235	5.25E+01	9.90E-06	4.11E-04
KCNE3	NM_005472	5.07E+03	1.13E-07	1.85E-05
KCNJ15	NM_002243	8.04E+01	1.73E-06	1.19E-04
KCNJ2	NM_000891	3.71E+03	3.59E-05	9.94E-04

KCTD21	NM_001029859	8.78E+02	2.99E-06	1.76E-04
KIAA0355	NM_014686	6.35E+02	1.08E-05	4.37E-04
KIAA1671	NM_001145206	2.14E+02	2.52E-05	7.58E-04
KIF1B	NM_183416	1.57E+02	8.41E-09	3.75E-06
KLF6	NM_001160124	1.78E+03	7.21E-06	3.28E-04
KLHL2	NM_001161521	8.30E+02	1.84E-05	6.14E-04
KLHL3	NM_017415	7.52E+01	2.22E-05	7.01E-04
KLLN	NM_001126049	6.40E+01	6.62E-08	1.29E-05
KNDC1	NM_152643	3.67E+01	1.54E-05	5.50E-04
LAMP2	NM_002294	2.99E+03	4.91E-06	2.50E-04
LAMTOR5	NM_006402	1.27E+03	1.84E-06	1.26E-04
LAPTM4A	NM_014713	2.67E+03	2.75E-06	1.65E-04
LBH	NM_030915	4.12E+03	5.03E-09	2.76E-06
LBR	NM_194442	1.20E+03	6.36E-06	3.01E-04
LDHA	NM_005566	5.92E+03	1.08E-08	4.45E-06
LDLRAP1	NM_015627	2.79E+03	1.52E-05	5.46E-04
LEF1	NM_016269	9.89E+02	6.33E-09	3.09E-06
LEPROT	NM_017526	1.73E+03	8.96E-06	3.82E-04
LHFPL2	NM_005779	6.39E+02	3.02E-06	1.77E-04
LIG3	NM_013975	4.64E+02	1.10E-08	4.45E-06
LIMK2	NM_016733	3.58E+02	6.89E-08	1.33E-05
LINC002481	NR_147207	1.83E+02	3.32E-06	1.89E-04
LINC00402	NR_144451	1.45E+02	2.78E-08	7.55E-06
LINC00565	NR_047495	1.05E+02	4.59E-06	2.40E-04
LINC00654	NR_015406	8.90E+01	4.16E-08	1.01E-05
LINC00861	NR_038446	5.41E+03	1.56E-06	1.10E-04
LINC00968	NR_038236	3.19E+01	5.63E-08	1.20E-05
LINC01238	NR_110592	6.08E+01	4.71E-06	2.44E-04
LINC01801	NR_033982	2.44E+01	1.21E-05	4.71E-04
LINC02035	NR_024618	1.26E+03	8.14E-10	7.16E-07
LINC02207	NR_120321	6.82E+01	5.71E-07	5.32E-05
LINC02210	NR_138257	1.17E+02	3.95E-06	2.14E-04
LINC02611	NR_126337	4.27E+01	6.88E-06	3.18E-04
LINC02648	NR_120634	1.26E+02	3.57E-05	9.89E-04
LINC02649	NR_040079	1.26E+02	8.20E-06	3.60E-04
LINC02656	NR_148966	1.42E+02	6.76E-07	6.09E-05
LINC02693	NR_160801	1.31E+02	1.91E-05	6.32E-04
LIPN	NM_001102469	1.42E+02	5.41E-07	5.15E-05

LMNB1	NM_001198557	2.57E+03	1.82E-09	1.39E-06
LOC100419170	NR_134873	7.48E+01	1.63E-05	5.68E-04
LOC100506123	NR_130704	7.34E+01	3.70E-07	4.04E-05
LOC100996724	NR_144516	1.43E+02	8.11E-08	1.45E-05
LOC101927759	NR_152848	1.44E+02	1.66E-05	5.72E-04
LOC101928143	NR_135251	3.39E+02	1.54E-05	5.50E-04
LOC101928429	NR_134635	1.65E+02	2.21E-06	1.43E-04
LOC102724159	NM_001368238.1	1.55E+02	2.41E-06	1.49E-04
LOC105369747	NR_135024	1.77E+01	3.09E-06	1.80E-04
LOC105377267	NR_136191	1.34E+02	4.56E-07	4.56E-05
LOC112268124	NR_160776	1.34E+02	3.87E-06	2.12E-04
LOC389906	NR_034031	1.01E+02	7.06E-06	3.23E-04
LOC728743	NR_027237	7.58E+02	3.45E-05	9.65E-04
LPGAT1	NM_014873	5.28E+03	1.38E-05	5.09E-04
LPIN1	NM_001349200	2.90E+01	4.70E-06	2.44E-04
LRATD2	NM_174911	2.28E+02	5.91E-06	2.85E-04
LRFN3	NM_024509	1.24E+02	3.37E-07	3.79E-05
LRIG1	NM_015541	5.11E+02	2.15E-10	3.79E-07
LRRC70	NM_181506	1.39E+02	3.01E-07	3.48E-05
LRRFIP1	NM_001137551	7.67E+02	1.24E-05	4.76E-04
LRRFIP2	NM_001134369	7.46E+02	5.30E-06	2.63E-04
LSMEM1	NM_001134468	3.36E+02	1.17E-08	4.60E-06
LTA4H	NR_132659	5.18E+02	2.24E-05	7.04E-04
LXN	NM_020169	1.72E+02	3.18E-05	9.10E-04
LY9	NM_001033667	1.92E+02	1.33E-07	2.07E-05
LY96	NM_001195797	7.07E+01	1.64E-05	5.69E-04
LYVE1	NM_006691	8.52E+01	2.14E-06	1.39E-04
MAFF	NM_001161572	1.81E+01	3.39E-07	3.80E-05
MAK	NM_001242385	1.98E+02	7.34E-07	6.55E-05
MAN1A1	NM_005907	2.13E+03	4.81E-07	4.75E-05
MAN1C1	NM_020379	3.51E+02	2.19E-08	6.48E-06
MAN2A2	NR_135503	2.91E+02	1.62E-05	5.68E-04
MANBA	NM_005908	2.15E+03	1.76E-05	5.95E-04
MANSC1	NM_018050	2.45E+03	9.07E-07	7.58E-05
MAP1LC3B	NM_022818	4.44E+03	8.83E-07	7.47E-05
MAP1LC3B2	NM_001085481	1.05E+03	8.88E-07	7.50E-05
MAP2K6	NM_001330450	7.12E+02	9.99E-07	8.03E-05
MAP3K2	NM_006609	4.40E+03	3.07E-05	8.88E-04

MAP3K20	NM_016653	2.81E+02	1.01E-05	4.18E-04
MAPK14	NM_139012	6.24E+03	1.54E-08	5.35E-06
MARC1	NM_022746	2.08E+03	1.27E-08	4.82E-06
MARCHF5	NM_017824	6.35E+02	1.48E-06	1.06E-04
MBD2	NM_003927	2.84E+03	1.06E-07	1.78E-05
MBD4	NM_001276270	1.02E+03	4.91E-06	2.50E-04
MBOAT2	NM_001321265	1.82E+02	2.42E-09	1.65E-06
MCEMP1	NM_174918	2.76E+03	2.03E-09	1.44E-06
MCM2	NM_004526	1.01E+02	3.28E-05	9.31E-04
MCM3	NM_001366372	3.92E+01	1.63E-05	5.68E-04
MCM4	NM_182746	1.53E+02	7.27E-06	3.30E-04
MCMBP	NM_024834	1.15E+03	4.72E-06	2.44E-04
MCOLN2	NM_153259	2.07E+02	2.15E-06	1.40E-04
MCTP1	NM_001002796	6.05E+02	2.53E-07	3.14E-05
MCTP2	NM_001159643	1.12E+03	8.31E-06	3.62E-04
MCU	NM_138357	6.57E+02	1.17E-05	4.59E-04
MDC1	NM_014641	9.03E+02	1.68E-08	5.73E-06
MDN1	NM_014611	1.11E+03	1.99E-05	6.46E-04
MDS2	NM_001348075	3.55E+01	1.03E-06	8.24E-05
ME1	NM_002395	4.48E+01	1.58E-05	5.61E-04
MEF2A	NM_001365204	7.00E+01	9.13E-08	1.60E-05
METTL7A	NM_014033	1.85E+03	6.59E-06	3.08E-04
METTL7B	NM_152637	4.75E+01	1.53E-05	5.48E-04
MFSD1	NM_001289407	7.27E+02	1.80E-05	6.06E-04
MGAM2	NM_001293626	4.83E+02	2.07E-06	1.37E-04
MGST1	NM_020300	5.01E+02	1.49E-08	5.24E-06
MGST2	NM_002413	4.64E+02	4.87E-07	4.77E-05
MICA-AS1	NR_148223	2.41E+02	3.30E-05	9.34E-04
MICAL3	NM_015241	4.45E+02	1.93E-05	6.34E-04
MID2	NM_052817	8.80E+01	2.86E-05	8.39E-04
MIR4697HG	NR_024344	3.22E+02	5.86E-08	1.20E-05
MIS18A	NM_018944	7.22E+01	7.71E-08	1.42E-05
MLLT3	NM_004529	1.81E+02	2.59E-07	3.19E-05
MLLT6	NM_005937	7.96E+03	1.40E-05	5.13E-04
MMADHC	NM_015702	1.77E+03	2.74E-07	3.28E-05
MORC2	NM_001303256	5.39E+02	1.38E-06	1.01E-04
MOSPD2	NM_152581	1.67E+03	3.35E-05	9.44E-04
MPP7	NM_173496	1.64E+02	3.16E-07	3.60E-05

MPRIP	NM_015134	1.46E+03	3.54E-05	9.83E-04
MPZL1	NM_024569	2.74E+03	6.34E-07	5.75E-05
MRNIP	NM_016175	2.07E+02	8.20E-06	3.60E-04
MRVI1	NM_130385	2.60E+01	7.30E-06	3.31E-04
MS4A4A	NM_148975	2.02E+02	1.07E-05	4.34E-04
MSH2	NM_000251	1.08E+02	2.42E-05	7.43E-04
MSL1	NM_001365921	3.84E+03	1.12E-08	4.45E-06
MSL3	NM_078629	1.27E+03	6.08E-10	6.30E-07
MSTO2P	NR_024117.1	1.34E+02	6.89E-06	3.18E-04
MTA1	NM_004689	4.99E+02	2.29E-05	7.16E-04
MTF1	NM_005955	1.75E+03	3.98E-06	2.15E-04
MTMR3	NM_153050	1.18E+03	2.79E-05	8.24E-04
MYADM	NM_001290194	5.11E+02	1.24E-05	4.77E-04
MYC	NM_002467	1.04E+03	3.58E-06	2.01E-04
MYL12A	NM_001303047	1.00E+02	2.44E-05	7.45E-04
MYLIP	NM_013262	2.23E+03	6.02E-06	2.88E-04
MYO10	NM_012334	4.45E+01	1.66E-06	1.15E-04
MYO7A	NM_001369365	7.08E+01	1.54E-05	5.50E-04
NAMPT	NM_005746	4.49E+04	1.38E-05	5.10E-04
NARF	NM_012336	1.73E+02	2.71E-06	1.63E-04
NDRG2	NM_201538	2.98E+01	3.07E-06	1.79E-04
NDUFB3	NM_002491	3.68E+02	1.68E-05	5.77E-04
NECAB1	NM_022351	5.64E+00	3.60E-07	3.95E-05
NEFL	NM_006158	9.27E+01	2.34E-05	7.28E-04
NELL2	NM_001145107	5.42E+01	3.42E-05	9.59E-04
NETO2	NM_018092	1.52E+02	2.25E-07	2.89E-05
NFATC2	NM_001258292	4.82E+01	3.03E-08	8.06E-06
NFATC2IP	NM_032815	1.98E+03	1.74E-07	2.45E-05
NFE2L2	NM_006164	3.10E+03	2.87E-05	8.42E-04
NFIL3	NM_005384	2.69E+03	5.19E-07	5.00E-05
NFKBIA	NM_020529	3.42E+03	2.66E-05	7.95E-04
NKD1	NM_033119	7.80E+01	2.50E-10	4.19E-07
NLRC3	NM_178844	2.10E+03	2.14E-10	3.79E-07
NLRC4	NM_021209	5.39E+02	1.29E-06	9.61E-05
NME8	NM_016616	1.82E+02	1.77E-05	5.96E-04
NMNAT1	NM_022787	4.53E+02	2.11E-07	2.80E-05
NMT2	NM_004808	3.20E+02	3.20E-05	9.12E-04
NOG	NM_005450	7.08E+01	7.24E-07	6.47E-05

NOL4L	NM_001256798	2.94E+02	1.08E-05	4.35E-04
NOL9	NM_024654	6.77E+02	3.73E-06	2.08E-04
NOP14	NM_001291978	6.83E+02	1.57E-05	5.59E-04
NOTCH2NLB	NM_001364007	2.65E+02	8.04E-06	3.57E-04
NPEPPS	NM_001330257	3.31E+03	9.62E-06	4.03E-04
NPR2	NM_003995	5.60E+01	3.91E-06	2.13E-04
NQO2	NM_000904	1.88E+02	4.53E-08	1.04E-05
NR4A1	NM_173157	8.06E+01	2.78E-05	8.22E-04
NRDC	NM_001242361	4.24E+03	1.97E-05	6.42E-04
NSUN3	NM_022072	4.89E+02	1.25E-05	4.80E-04
NSUN7	NM_024677	3.54E+02	1.85E-06	1.26E-04
NT5C2	NM_001351176	2.49E+02	2.87E-06	1.72E-04
NUDT16	NM_152395	1.69E+03	8.50E-07	7.28E-05
NUP210	NM_024923	3.57E+03	2.44E-05	7.45E-04
OBSCN	NM_001271223	3.47E+02	2.57E-06	1.56E-04
OLAH	NM_018324	3.47E+02	1.58E-10	3.10E-07
OLFM2	NM_001304348	1.38E+02	8.73E-07	7.41E-05
OLIG2	NM_005806	7.59E+01	4.29E-10	5.97E-07
OMG	NM_002544	9.46E+01	1.52E-06	1.08E-04
OSBPL1A	NM_001242508	1.68E+02	8.70E-06	3.75E-04
OSTF1	NM_012383	4.08E+03	2.35E-06	1.49E-04
OTUD7B	NM_020205	1.44E+02	1.10E-07	1.81E-05
OTULINL	NM_019018	1.33E+03	3.20E-10	4.86E-07
P4HA1	NM_000917	5.34E+02	9.76E-08	1.67E-05
PACSIN1	NM_020804	5.19E+01	2.31E-07	2.94E-05
PAFAH2	NM_000437	8.66E+02	3.94E-07	4.23E-05
PASK	NM_015148	1.60E+02	1.30E-07	2.04E-05
PATJ	NM_176877	2.35E+02	8.65E-07	7.38E-05
PCED1B	NM_138371	7.38E+02	1.02E-08	4.34E-06
PCED1B-AS1	NR_026544	8.57E+02	2.69E-11	1.32E-07
PCMT1	NM_001360456	3.35E+02	3.88E-06	2.12E-04
PCNT	NM_006031	6.96E+02	2.04E-07	2.74E-05
PCNX1	NM_001308160	3.29E+03	1.31E-06	9.71E-05
PCNX2	NM_014801	4.40E+02	1.21E-07	1.95E-05
PCTP	NM_021213	1.01E+03	7.23E-06	3.29E-04
PDZD8	NM_173791	1.14E+03	1.11E-05	4.43E-04
PEBP1	NM_002567	1.42E+03	7.60E-10	7.06E-07
PECR	NM_018441	2.75E+02	1.22E-07	1.95E-05

PFAS	NM_012393	2.39E+02	4.40E-06	2.33E-04
PFKFB2	NM_001018053	5.49E+02	1.25E-06	9.34E-05
PFKFB3	NR_136554	4.85E+02	2.24E-08	6.57E-06
PGK1	NM_000291	1.64E+04	7.53E-06	3.40E-04
PGM2	NM_018290	1.41E+03	3.52E-08	9.13E-06
PHC1	NM_004426	8.25E+02	4.08E-07	4.32E-05
PHF19	NM_015651	3.93E+02	1.22E-05	4.72E-04
PHKA2	NM_000292	2.08E+03	6.85E-06	3.17E-04
PHTF1	NM_001323042	2.08E+02	4.70E-06	2.44E-04
PIGU	NM_080476	2.34E+02	2.76E-05	8.18E-04
PIK3C2B	NM_002646	5.58E+02	3.29E-07	3.71E-05
PIK3CG	NM_001282427	1.45E+03	1.17E-05	4.61E-04
PIK3IP1	NM_052880	3.38E+03	2.54E-06	1.55E-04
PIM2	NM_006875	4.96E+03	7.72E-06	3.47E-04
PIP4P2	NM_018710	1.34E+03	2.03E-06	1.34E-04
PKD1P4-NPIPA8	NR_146336	8.36E+01	3.06E-08	8.06E-06
PKD1P5-LOC105376752	NR_146331	3.53E+02	1.77E-07	2.48E-05
PLA2G4A	NM_024420	2.12E+02	1.73E-05	5.89E-04
PLBD1	NM_024829	1.71E+04	6.45E-08	1.28E-05
PLBD1-AS1	NR_120465	1.82E+01	2.05E-05	6.59E-04
PLCG1	NM_002660	1.96E+03	1.60E-07	2.29E-05
PLD4	NM_001308174	8.33E+01	4.18E-07	4.35E-05
PLEKHB1	NM_001130035	2.35E+02	2.37E-06	1.49E-04
PLIN3	NM_001164194	3.72E+02	2.41E-05	7.40E-04
PLXDC1	NM_020405	4.04E+02	2.95E-06	1.74E-04
PLXDC2	NM_032812	3.23E+03	1.66E-05	5.72E-04
PLXNA3	NM_017514	6.22E+02	1.11E-05	4.43E-04
PLXNA4	NM_020911	4.66E+01	7.99E-08	1.44E-05
PLXNC1	NR_037687	9.70E+03	5.26E-07	5.03E-05
PNPLA1	NM_173676	9.62E+01	1.73E-05	5.90E-04
POLR1A	NM_015425	4.96E+02	1.64E-05	5.68E-04
POLR3D	NM_001722	3.83E+02	1.47E-07	2.19E-05
POU6F1	NM_001368008	9.56E+01	1.75E-05	5.92E-04
PPARG	NM_138712	1.38E+01	3.69E-14	6.17E-10
PPM1K	NM_152542	1.02E+03	1.76E-05	5.95E-04
PPP1R12A	NM_001244992	5.26E+02	1.29E-05	4.88E-04
PPP1R13B	NM_015316	2.37E+02	3.10E-06	1.80E-04

PPP1R16B	NM_001172735	7.83E+02	3.75E-07	4.05E-05
PPP1R21	NM_001135629	8.39E+02	1.36E-05	5.06E-04
PPP1R3B	NM_001201329	1.45E+03	9.11E-08	1.60E-05
PPP1R3D	NM_006242	1.22E+03	7.45E-09	3.48E-06
PPP2R5A	NM_001199756	3.43E+03	2.40E-06	1.49E-04
PPP3CC	NM_005605	4.01E+02	3.19E-05	9.11E-04
PPP4R1	NR_052003	1.40E+03	2.10E-08	6.39E-06
PRAG1	NM_001080826	2.75E+02	7.85E-06	3.52E-04
PRCP	NM_005040	4.73E+03	1.24E-05	4.77E-04
PRDM5	NM_018699	3.97E+01	1.16E-06	8.97E-05
PRDX3	NM_006793	1.65E+03	1.56E-06	1.11E-04
PRKCA	NM_002737	8.15E+02	5.05E-07	4.90E-05
PRKCB	NM_002738	1.08E+04	9.42E-06	3.97E-04
PRKCD	NM_001354679	1.51E+02	3.17E-12	2.64E-08
PRKCQ	NM_006257	4.86E+02	2.26E-07	2.89E-05
PRKCQ-AS1	NR_036503	2.26E+02	2.88E-07	3.40E-05
PROK2	NM_021935	3.80E+03	8.25E-06	3.61E-04
PRSS33	NM_152891	2.17E+02	8.02E-09	3.62E-06
PSEN1	NM_000021	2.65E+03	4.98E-06	2.52E-04
PSMD6	NM_001271781	7.95E+02	2.17E-05	6.85E-04
PSTPIP2	NM_024430	2.20E+03	8.05E-06	3.57E-04
PTEN	NM_001304718	6.80E+02	2.07E-06	1.36E-04
PTENP1	NR_023917	1.93E+03	1.69E-05	5.78E-04
PTGDR2	NM_004778	3.98E+02	4.44E-09	2.47E-06
PTPRC	NM_001267798	2.33E+02	2.75E-07	3.28E-05
PTPRS	NM_130854	2.41E+01	1.21E-05	4.71E-04
PWP1	NM_007062	4.22E+02	2.51E-05	7.55E-04
PWP2	NM_005049.1	1.55E+02	2.41E-06	1.49E-04
PXYLP1	NM_001037172	2.38E+02	8.46E-07	7.28E-05
PYGL	NM_001163940	3.84E+03	6.27E-07	5.71E-05
PYHIN1	NM_152501	3.60E+02	5.88E-07	5.43E-05
QPCT	NM_012413	5.07E+03	5.83E-08	1.20E-05
RAB10	NM_016131	3.53E+03	4.06E-06	2.19E-04
RAB13	NM_002870	7.40E+01	1.59E-05	5.62E-04
RAB1A	NM_004161	2.15E+03	6.68E-06	3.10E-04
RAB27A	NM_183235	2.13E+03	3.10E-06	1.80E-04
RAB2A	NM_002865	1.23E+03	7.19E-07	6.46E-05
RAB31	NM_006868	7.65E+03	9.44E-07	7.79E-05

RAB32	NM_006834	1.93E+03	2.54E-07	3.15E-05
RAB40B	NM_006822	4.27E+01	2.06E-07	2.74E-05
RAB5IF	NM_199483	8.15E+01	7.03E-06	3.22E-04
RAB7A	NM_004637	1.13E+04	1.46E-05	5.33E-04
RABIF	NM_002871	7.95E+02	2.92E-05	8.51E-04
RAF1	NM_001354690	4.77E+03	2.47E-06	1.52E-04
RALB	NM_002881	3.76E+03	2.96E-07	3.44E-05
RALGAPA2	NM_020343	2.84E+03	5.57E-07	5.26E-05
RAMAC	NM_031452	6.87E+02	5.81E-06	2.82E-04
RAP1A	NM_002884	1.19E+03	1.81E-07	2.51E-05
RASA3	NM_001320822	1.23E+03	9.73E-08	1.67E-05
RASGRF2	NM_006909	3.29E+02	2.61E-07	3.19E-05
RASGRP4	NM_001146206	1.62E+03	1.74E-05	5.92E-04
RASSF2	NM_170774	1.87E+04	2.50E-06	1.53E-04
RBM47	NM_001371114	1.13E+03	4.00E-08	9.98E-06
RBMS1	NM_016836	1.55E+03	2.06E-07	2.74E-05
RC3H1	NM_001300852	6.56E+02	2.34E-05	7.29E-04
RCAN3	NM_013441	3.19E+02	3.36E-06	1.92E-04
RCBTB2	NM_001352428	6.03E+02	2.00E-05	6.50E-04
RELA	NM_001243984	4.60E+02	1.36E-05	5.05E-04
REPS2	NM_001080975	9.41E+02	1.61E-05	5.67E-04
RFTN1	NM_015150	1.13E+03	8.13E-07	7.04E-05
RFX2	NM_000635	7.15E+02	3.95E-06	2.14E-04
RGS18	NM_130782	2.93E+03	3.12E-05	8.97E-04
RGS2	NM_002923	2.22E+04	3.00E-05	8.69E-04
RHEB	NM_005614	3.59E+02	7.86E-06	3.52E-04
RHOH	NM_001278363	4.65E+02	1.91E-08	6.13E-06
RHOT1	NM_001288754	3.66E+02	4.19E-06	2.24E-04
RHOU	NR_037962	3.00E+02	9.11E-06	3.86E-04
RIC3	NM_001206671	2.40E+01	1.81E-05	6.07E-04
RILPL2	NM_145058	2.13E+03	6.38E-06	3.01E-04
RIOX1	NM_024644	3.31E+02	8.30E-06	3.62E-04
RIT1	NM_006912	1.14E+03	1.18E-05	4.62E-04
RMDN3	NM_018145	1.92E+02	4.67E-06	2.44E-04
RNASE1	NM_002933	6.38E+01	6.03E-08	1.23E-05
RNASE4	NM_002937	1.76E+02	1.10E-05	4.41E-04
RNASE6	NM_005615	1.62E+03	1.09E-05	4.37E-04
RNF130	NM_018434	8.02E+03	1.36E-06	1.00E-04

RNF135	NM_032322	1.13E+03	3.17E-05	9.10E-04
RNF141	NM_016422	2.49E+03	2.28E-05	7.14E-04
RNF144A	NM_014746	1.00E+02	3.85E-06	2.12E-04
RNF144B	NM_182757	2.14E+03	5.30E-06	2.63E-04
RNF146	NM_001242850	4.66E+02	1.58E-05	5.59E-04
RNF149	NM_173647	1.25E+04	2.11E-05	6.72E-04
RNF157	NM_052916	4.84E+02	2.95E-06	1.74E-04
RNF207	NM_207396	5.63E+01	9.09E-07	7.58E-05
RNU6ATAC35P	NR_157260	2.36E+01	1.63E-05	5.68E-04
RRAGA	NM_006570	1.38E+03	3.37E-05	9.48E-04
RRAGD	NM_021244	7.35E+02	1.21E-06	9.21E-05
RRP1B	NM_015056	9.13E+02	1.08E-07	1.80E-05
RRS1	NM_015169	2.41E+02	2.53E-05	7.61E-04
RSRC2	NM_023012	6.54E+02	2.40E-05	7.39E-04
RTL10	NM_024627	5.61E+02	1.44E-06	1.04E-04
RTL6	NM_032287	6.46E+02	3.41E-05	9.59E-04
RTN3	NM_201429	1.59E+03	1.87E-06	1.26E-04
RUNX3	NM_004350	1.19E+03	5.21E-06	2.61E-04
S1PR5	NM_030760	9.31E+02	1.89E-05	6.26E-04
SALL2	NM_001364564	3.35E+01	4.29E-06	2.29E-04
SAMSN1	NM_022136	1.01E+03	1.31E-08	4.86E-06
SAP30	NM_003864	2.11E+02	4.44E-06	2.34E-04
SAP30L	NM_024632	1.29E+03	4.25E-06	2.27E-04
SARDH	NM_001134707	4.61E+01	3.08E-05	8.90E-04
SARM1	NM_015077	8.28E+02	9.64E-09	4.19E-06
SAT1	NM_002970	1.11E+04	9.06E-07	7.58E-05
SBK1	NM_001024401	6.11E+02	5.41E-06	2.66E-04
SCART1	NR_149718	6.09E+01	1.39E-06	1.01E-04
SCN1B	NM_199037	4.44E+01	1.02E-05	4.19E-04
SCPEP1	NM_021626	3.37E+03	1.42E-07	2.16E-05
SDC2	NM_002998	7.29E+01	6.61E-06	3.08E-04
SDCBP	NM_001007069	3.31E+03	1.50E-05	5.41E-04
SDHAF3	NM_020186	1.49E+02	6.11E-06	2.92E-04
SDHB	NM_003000	1.26E+03	2.62E-05	7.86E-04
SDHC	NM_003001	1.59E+03	2.09E-05	6.69E-04
SDR42E1	NM_145168	1.61E+02	2.63E-05	7.88E-04
SEC11A	NM_014300	1.60E+03	1.35E-05	5.05E-04
SEC62	NM_003262	2.28E+03	2.67E-05	7.97E-04

SEMA5A	NM_003966	1.74E+01	1.85E-05	6.17E-04
SEPHS2	NM_012248	2.53E+03	4.16E-08	1.01E-05
SEPTIN1	NM_001365977	1.41E+03	1.96E-05	6.40E-04
SEPTIN6	NM_145799	4.68E+03	1.70E-07	2.43E-05
SERINC1	NM_020755	7.34E+03	2.53E-05	7.61E-04
SERPINB1	NM_030666	9.49E+03	5.47E-10	6.30E-07
SETD7	NM_030648	7.38E+02	1.07E-05	4.35E-04
SFI1	NM_001007467	4.61E+02	1.66E-06	1.15E-04
SFMBT1	NM_016329	2.85E+02	1.86E-06	1.26E-04
SGMS2	NM_152621	3.05E+01	2.01E-05	6.52E-04
SH2D2A	NM_001161442	8.86E+01	1.32E-05	4.96E-04
SH3GLB1	NM_016009	5.36E+03	2.06E-08	6.33E-06
SH3PXD2B	NM_001017995	6.29E+01	4.26E-07	4.41E-05
SIGLEC8	NM_014442	1.48E+02	5.49E-13	6.12E-09
SIRPA	NM_080792	6.79E+02	5.62E-06	2.75E-04
SIRPG	NM_018556	4.66E+02	1.09E-06	8.54E-05
SKAP1	NM_003726	8.90E+02	3.45E-05	9.65E-04
SKAP2	NM_003930	3.85E+03	7.76E-08	1.42E-05
SLA	NM_001282965	5.73E+03	1.54E-06	1.10E-04
SLC15A4	NM_145648	2.61E+03	3.42E-05	9.59E-04
SLC16A6	NM_004694	5.49E+02	2.39E-05	7.38E-04
SLC17A5	NM_012434	4.76E+02	8.05E-08	1.45E-05
SLC1A3	NM_004172	3.95E+01	2.43E-07	3.04E-05
SLC20A2	NM_006749	5.50E+01	1.50E-07	2.22E-05
SLC22A15	NM_018420	7.71E+02	2.86E-05	8.39E-04
SLC22A17	NM_016609	2.90E+01	1.57E-05	5.58E-04
SLC22A4	NM_003059	1.15E+03	1.97E-09	1.43E-06
SLC24A4	NM_153646	7.97E+02	2.93E-06	1.74E-04
SLC25A23	NM_024103	2.87E+02	2.14E-07	2.82E-05
SLC25A4	NM_001151	7.16E+01	7.99E-06	3.56E-04
SLC2A3	NM_006931	1.43E+04	1.61E-08	5.56E-06
SLC31A1	NM_001859	1.12E+03	1.27E-07	2.01E-05
SLC31A2	NM_001860	3.94E+03	1.49E-09	1.18E-06
SLC36A1	NM_078483	1.27E+03	1.71E-05	5.84E-04
SLC37A3	NM_001363375	3.44E+02	1.63E-05	5.68E-04
SLC38A1	NM_001278388	2.70E+02	5.80E-06	2.82E-04
SLC40A1	NM_014585	3.63E+03	3.74E-07	4.05E-05
SLC41A1	NM_173854	5.25E+02	9.36E-07	7.74E-05

SLC49A4	NM_032839	5.22E+02	1.70E-08	5.73E-06
SLC5A9	NM_001135181	3.62E+01	6.89E-08	1.33E-05
SLC7A6	NM_003983	1.75E+03	1.86E-05	6.19E-04
SLC7A7	NM_001126105	8.65E+02	1.45E-07	2.18E-05
SLC8A1	NM_001351487	3.61E+01	2.14E-08	6.40E-06
SLC9A7P1	NR_033801	1.52E+02	1.07E-07	1.79E-05
SLFN13	NM_144682	1.06E+03	8.24E-06	3.61E-04
SLITRK4	NM_001184750	4.93E+01	4.26E-08	1.02E-05
SMAP2	NM_001198979	2.13E+03	1.35E-05	5.03E-04
SMIM10L2A	NM_203306	3.43E+01	2.68E-06	1.62E-04
SMPD3	NM_018667	3.95E+02	3.26E-09	2.08E-06
SMYD4	NM_052928	4.64E+02	1.88E-05	6.22E-04
SNPH	NM_014723	2.08E+02	9.30E-06	3.93E-04
SNRPN	NM_003097	6.42E+02	5.62E-07	5.26E-05
SNURF	NM_005678	6.42E+02	5.62E-07	5.26E-05
SNX2	NM_003100	1.88E+03	2.35E-05	7.31E-04
SNX27	NM_001330723	2.82E+03	2.25E-07	2.89E-05
SNX3	NM_003795	2.88E+03	2.02E-06	1.34E-04
SORBS3	NM_005775	1.48E+02	1.63E-05	5.68E-04
SORT1	NM_001205228	9.15E+02	4.08E-07	4.32E-05
SOX8	NM_014587	3.28E+01	1.31E-06	9.71E-05
SP100	NM_003113	4.71E+02	3.51E-05	9.77E-04
SPAG6	NM_012443	8.11E+00	1.31E-05	4.95E-04
SPIN3	NM_001010862	2.15E+02	3.21E-05	9.16E-04
SPN	NM_003123	2.84E+03	6.65E-06	3.09E-04
SPNS3	NM_182538	1.01E+02	1.27E-05	4.85E-04
SPOCK1	NM_004598	3.76E+01	1.85E-05	6.17E-04
SPOCK2	NM_001134434	5.60E+01	4.56E-07	4.56E-05
SPON1	NM_006108	4.19E+01	1.04E-06	8.28E-05
SPPL2A	NM_032802	1.58E+03	7.87E-06	3.52E-04
SPTLC1	NM_006415	8.86E+02	2.11E-05	6.71E-04
SPTLC2	NM_004863	4.90E+03	1.16E-06	8.94E-05
SRGAP1	NM_020762	1.45E+01	2.80E-07	3.33E-05
SRGAP3	NM_014850	3.55E+01	2.23E-06	1.43E-04
SRGN	NM_002727	4.24E+04	9.94E-08	1.69E-05
SRPK1	NM_003137	3.06E+03	1.03E-08	4.34E-06
SRSF8	NM_032102	8.43E+02	2.88E-06	1.72E-04
ST3GAL6	NM_001323368	3.84E+02	5.33E-06	2.63E-04

ST6GALNAC3	NM_152996	4.71E+01	5.51E-07	5.23E-05
STAT4	NM_003151	1.45E+03	4.76E-08	1.07E-05
STOM	NR_073037	1.92E+02	2.12E-05	6.72E-04
STX11	NM_003764	3.42E+03	2.78E-05	8.22E-04
STXBP1	NM_001032221	3.84E+01	2.10E-06	1.37E-04
SULT1B1	NM_014465	3.89E+03	7.34E-08	1.37E-05
SUMF1	NM_182760	7.15E+02	4.68E-07	4.66E-05
SUPT20H	NM_001278480	1.20E+03	6.22E-08	1.24E-05
SVIL	NM_003174	2.24E+03	1.60E-05	5.62E-04
SYNGAP1	NM_006772	3.70E+02	2.10E-06	1.37E-04
SYTL2	NM_032943	1.63E+01	1.13E-05	4.49E-04
TAF12	NM_005644	8.40E+02	3.19E-05	9.12E-04
TANK	NM_001199135	1.19E+03	1.57E-05	5.58E-04
TARBP1	NM_005646	4.75E+02	9.70E-06	4.05E-04
TARP	NM_001003799	3.63E+02	2.07E-05	6.65E-04
TBC1D15	NM_022771	2.34E+02	1.39E-05	5.12E-04
TBC1D8	NM_001330348	2.12E+02	6.44E-08	1.28E-05
TCF7	NM_201632	1.28E+03	1.41E-09	1.15E-06
TDRD9	NM_153046	2.26E+02	2.81E-09	1.88E-06
TEF	NM_003216	1.88E+02	5.06E-07	4.90E-05
TENT4A	NM_006999	5.91E+02	1.43E-06	1.04E-04
TESPA1	NM_001136030	8.77E+02	2.77E-11	1.32E-07
TGFA	NM_001308158	8.03E+01	5.34E-09	2.78E-06
THEM4	NM_053055	8.59E+02	1.99E-06	1.32E-04
TIFAB	NM_001099221	1.04E+02	8.53E-06	3.70E-04
TIGIT	NM_173799	7.21E+02	6.62E-11	1.72E-07
TK2	NM_001172643	5.76E+02	5.00E-07	4.87E-05
TLE4	NM_001351556	1.95E+02	8.13E-06	3.59E-04
TLR2	NM_001318790	1.49E+03	7.89E-07	6.91E-05
TLR4	NM_138557	3.01E+03	1.21E-05	4.71E-04
TLR5	NM_003268	2.43E+03	2.67E-10	4.24E-07
TLR8	NM_016610	1.25E+03	3.34E-09	2.08E-06
TMBIM4	NM_016056	2.65E+03	2.86E-05	8.39E-04
TMED8	NM_001346134	3.59E+02	2.28E-06	1.46E-04
TMEM120B	NM_001080825	1.74E+02	2.73E-05	8.11E-04
TMEM144	NM_018342	1.80E+02	6.42E-10	6.30E-07
TMEM165	NM_018475	7.79E+02	2.58E-05	7.73E-04
TMEM167A	NM_174909	2.74E+03	1.06E-05	4.34E-04

TMEM167B	NM_020141	1.97E+03	1.47E-05	5.37E-04
TMEM236	NM_001098844	1.65E+01	2.37E-05	7.35E-04
TMEM260	NM_017799	9.25E+02	4.29E-08	1.02E-05
TMEM30B	NM_001017970	3.13E+01	6.30E-06	2.98E-04
TMEM33	NM_018126	1.73E+03	1.72E-06	1.18E-04
TMEM59	NM_004872	3.53E+03	9.55E-07	7.82E-05
TMEM70	NR_033334	1.10E+02	1.39E-05	5.12E-04
TMEM88	NM_001319941	2.03E+01	4.69E-06	2.44E-04
TMEM8B	NM_001363622	5.00E+01	2.41E-06	1.49E-04
TMLHE	NM_018196	4.05E+02	6.57E-06	3.08E-04
TMX4	NM_021156	4.91E+03	2.65E-06	1.61E-04
TNFAIP8L1	NM_152362	3.30E+02	9.84E-06	4.09E-04
TNFAIP8L3	NM_001311175	5.94E+00	2.02E-08	6.24E-06
TNFRSF21	NM_014452	2.78E+01	5.60E-07	5.26E-05
TNFSF14	NM_172014	2.46E+03	1.02E-06	8.21E-05
TNIK	NM_001161561	1.10E+02	2.60E-06	1.58E-04
TP53I3	NM_147184	8.88E+01	1.32E-05	4.97E-04
TP73-AS1	NR_033711	4.86E+02	1.21E-05	4.72E-04
TPST2	NM_001362923	1.93E+02	1.18E-06	9.02E-05
TRAF1	NM_005658	1.75E+03	5.87E-06	2.84E-04
TRAF5	NM_001033910	3.48E+02	3.44E-06	1.95E-04
TRERF1	NM_001297573	9.04E+02	1.14E-06	8.82E-05
TRIB2	NM_021643	5.39E+02	8.04E-07	6.98E-05
TRIM27	NM_006510	4.03E+03	5.53E-07	5.24E-05
TRIP4	NM_016213	7.48E+02	9.29E-06	3.93E-04
TRIQK	NM_001171795	1.26E+02	1.24E-05	4.76E-04
TRPS1	NM_001282902	2.20E+02	1.92E-06	1.29E-04
TSC22D3	NM_001015881	8.01E+03	8.76E-06	3.76E-04
TSEN34	NM_024075	3.04E+02	3.25E-05	9.25E-04
TSHZ2	NM_173485	7.38E+01	2.06E-09	1.44E-06
TSPAN14	NM_001351267	5.33E+02	4.77E-06	2.46E-04
TSPAN18	NM_130783	5.71E+02	7.75E-09	3.55E-06
TSPAN2	NM_005725	1.21E+03	3.62E-05	1.00E-03
TSPOAP1-AS1	NR_038415	4.02E+02	2.38E-05	7.37E-04
TSPYL2	NM_022117	8.85E+02	4.71E-07	4.68E-05
TTC28-AS1	NR_026963	1.32E+02	1.08E-05	4.35E-04
TULP3	NM_003324	1.59E+02	1.94E-06	1.29E-04
TXNRD1	NM_182743	7.87E+02	3.49E-05	9.72E-04

UBASH3A	NM_001001895	4.24E+02	2.13E-08	6.40E-06
UBE2B	NM_003337	2.60E+03	2.04E-05	6.57E-04
UBE2G2	NM_003343	1.51E+03	2.03E-05	6.56E-04
UBE2J1	NM_016021	4.37E+03	3.91E-10	5.68E-07
UBE2Q2	NM_001145335	1.02E+02	1.27E-05	4.84E-04
UBE2R2	NM_017811	6.05E+03	1.39E-05	5.12E-04
UBFD1	NM_019116	6.41E+02	5.24E-06	2.62E-04
UBIAD1	NM_013319	5.58E+02	2.12E-06	1.39E-04
UBXN2B	NM_001077619	2.45E+03	9.91E-06	4.11E-04
UGCG	NM_003358	1.00E+03	4.38E-06	2.33E-04
UGP2	NM_001001521	2.11E+03	2.99E-05	8.68E-04
UPP1	NM_003364	1.09E+03	2.28E-05	7.14E-04
USB1	NM_001330568	2.79E+03	1.52E-05	5.47E-04
USP15	NR_147079	9.02E+02	2.99E-05	8.68E-04
USP36	NM_001321291	1.06E+03	1.57E-07	2.28E-05
VAPA	NM_194434	3.75E+03	1.06E-05	4.31E-04
VAV3	NM_001079874	7.10E+02	1.55E-07	2.27E-05
VDAC2	NM_001324087	1.87E+01	1.28E-05	4.87E-04
VIM	NM_003380	3.55E+04	2.39E-08	6.76E-06
VMAC	NM_001017921	3.36E+02	2.14E-05	6.78E-04
VMP1	NM_001329395	1.98E+03	3.35E-06	1.91E-04
VNN1	NM_004666	2.59E+03	6.95E-11	1.72E-07
VSIG1	NM_182607	2.07E+02	5.18E-08	1.15E-05
VSIG4	NM_007268	3.51E+02	5.31E-08	1.15E-05
WASF1	NM_003931	3.65E+01	2.82E-06	1.69E-04
WDFY2	NM_052950	2.81E+03	1.58E-07	2.29E-05
WDFY3	NM_014991	3.15E+03	3.45E-05	9.65E-04
WHAMM	NM_001080435	6.00E+02	2.72E-07	3.28E-05
WNT10B	NM_003394	1.84E+02	1.87E-05	6.20E-04
WNT7A	NM_004625	4.49E+01	6.23E-07	5.69E-05
WTAP	NM_001270531	9.85E+02	5.63E-06	2.75E-04
YIPF1	NM_018982	5.44E+02	1.17E-06	9.02E-05
ZBTB4	NM_001128833	1.47E+03	2.81E-07	3.33E-05
ZBTB9	NM_152735	2.95E+02	3.92E-06	2.13E-04
ZC3H12D	NM_207360	6.47E+02	1.16E-09	9.66E-07
ZDHHC3	NR_146162	2.69E+02	6.69E-07	6.04E-05
ZDHHC9	NM_001008222	1.82E+01	1.84E-06	1.26E-04
ZEB2	NR_033258	1.13E+03	8.21E-08	1.46E-05

ZNF106	NM_001284306	7.90E+02	2.29E-05	7.15E-04
ZNF142	NM_001366291	6.66E+01	2.24E-05	7.04E-04
ZNF148	NM_001348426	1.66E+02	3.60E-06	2.02E-04
ZNF195	NM_001242843	3.97E+01	3.21E-06	1.84E-04
ZNF230	NM_006300	4.99E+02	2.49E-05	7.55E-04
ZNF251	NM_138367	2.33E+02	1.83E-07	2.51E-05
ZNF275	NM_001367757	2.18E+02	1.19E-05	4.64E-04
ZNF395	NM_018660	9.44E+02	1.76E-06	1.20E-04
ZNF41	NM_001324148	1.80E+01	2.46E-06	1.51E-04
ZNF438	NM_182755	1.05E+02	3.29E-05	9.32E-04
ZNF608	NM_020747	2.01E+02	1.43E-07	2.17E-05
ZNF696	NM_030895	1.97E+02	7.50E-07	6.65E-05
ZNF710-AS1	NR_146321	4.52E+01	7.21E-07	6.47E-05
ZNF786	NM_152411	1.80E+02	1.19E-06	9.10E-05
ZNF792	NM_175872	2.82E+02	6.48E-06	3.05E-04
ZNF815P	NR_023382	1.99E+02	1.31E-05	4.96E-04
ZNF831	NM_178457	8.74E+02	1.02E-05	4.19E-04
ZNF853	NM_001353546	9.61E+01	3.87E-06	2.12E-04
ZSWIM5	NM_020883	6.34E+01	1.72E-07	2.45E-05
ZSWIM6	NM_020928	9.29E+02	4.86E-06	2.48E-04

**Supplementary Table S2: Supplementary Fig S1 PC1 top 175 genes**		
**Gene symbol**	**Transcript ID**	**Eigen value**
HAUS5	NM_015302	0.94536293
PCNX2	NM_014801	0.94105664
MDC1	NM_014641	0.93090761
ATP8B2	NM_020452	0.92906926
POLR3D	NM_001722	0.92534424
USP36	NM_001321291	0.92214609
LBH	NM_030915	0.92156862
PLCG1	NM_002660	0.92043907
PEBP1	NM_002567	0.91930597
ATP8B2	NM_001370597	0.91910184
CD5	NM_014207	0.91844281
SARM1	NM_015077	0.91662356
TCF7	NM_001346450	0.91215434
ATP8B2	NM_001367934	0.91090246
ZC3H12D	NM_207360	0.91001676
BCL11B	NM_001282237	0.90723031
BCL11B	NM_138576	0.90480507
UBIAD1	NM_013319	0.9040638
EVL	NM_016337	0.90284636
IFFO2	NM_001136265	0.90124191
PPP1R13B	NM_015316	0.90062315
ZBTB4	NM_001128833	0.89952177
TCF7	NM_201632	0.89704588
UBASH3A	NM_001001895	0.89610311
TSPAN18	NM_130783	0.89489175
GATA3	NM_001002295	0.89379407
RASA3	NM_001320822	0.89237186
CZ1P-ASNS	NR_147989	0.89234791
ESYT1	NM_015292	0.88937369
FAM171A1	NM_001010924	0.88844921
CD3E	NM_000733	0.88712971
ANKS6	NM_173551	0.88649495
LRIG1	NM_015541	0.88540474
HABP4	NM_014282	0.88416892
TSPYL2	NM_022117	0.88389191
ATP10A	NM_024490	0.88353457

LRFN3	NM_024509	0.88344204
SLC25A23	NM_024103	0.88318981
FAM102A	NM_001035254	0.88234567
ING5	NM_032329	0.88069268
B4GAT1	NM_006876	0.88061856
RIOX1	NM_024644	0.87793326
LY9	NM_001033667	0.87637685
IL11RA	NR_052010	0.87598242
TCF7	NM_001134851	0.8725878
GOLGA7B	NM_001010917	0.87235043
PIK3C2B	NM_002646	0.87144674
ZNF395	NM_018660	0.87084969
FMNL3	NM_198900	0.87045803
PLXNA3	NM_017514	0.87044155
AMIGO1	NM_020703	0.87015852
WNT7A	NM_004625	0.86951742
BDH1	NM_004051	0.86762059
CHMP7	NM_152272	0.86710648
PLD4	NM_138790	0.86658289
AQP3	NM_004925	0.86556604
SIRPG	NM_018556	0.86435739
MAN1C1	NM_020379	0.86431176
WHAMM	NM_001080435	0.86217933
FBXL16	NM_153350	0.8621217
TEF	NM_003216	0.86102166
TESPA1	NM_001136030	0.86083043
PCNT	NM_006031	0.86014106
MLLT6	NM_005937	0.85977604
TCF7	NM_213648	0.8597653
ARL4C	NM_005737	0.85969087
GAREM2	NM_001168241	0.85964926
PACSIN1	NM_020804	0.85926782
PFAS	NM_012393	0.85793681
MIR4697HG	NR_024344	0.85757589
TRAF1	NM_005658	0.85630476
NFATC2	NM_001258292	0.85539733
DHRS3	NM_004753	0.85538068
NLRC3	NM_178844	0.85515458

PLCG1	NM_182811	0.85513673
SBK1	NM_001024401	0.85491739
SFI1	NM_001007467	0.85417973
FAM102A	NM_203305	0.85411923
SPOCK2	NM_014767	0.85401253
SOX8	NM_014587	0.85392731
DGCR8	NM_022720	0.85294732
IFT172	NM_015662	0.85154353
PPP1R16B	NM_015568	0.85041406
KCNA3	NM_002232	0.85021848
TENT4A	NM_006999	0.84962071
TRIB2	NM_021643	0.84733536
FAN1	NM_014967	0.84719885
TCF7	NR_033449	0.84667227
PLD4	NM_001308174	0.84590059
CDC42BPG	NM_017525	0.84489656
ACACB	NM_001093	0.84479521
CCDC88C	NM_001080414	0.84433201
SPOCK2	NM_001244950	0.84195153
TTC28-AS1	NR_026963	0.84067294
PASK	NM_015148	0.84057525
SLC20A2	NM_006749	0.84022672
FMNL3	NM_175736	0.83924613
AXIN2	NM_004655	0.83898273
DOCK9-DT	NR_047482	0.83893864
MORC2	NM_001303256	0.83757956
CCDC85C	NM_001144995	0.83755105
ECHDC2	NM_001319958	0.83675132
NOL4L	NM_001256798	0.83628062
RAB40B	NM_006822	0.83554354
PLEKHB1	NM_001130035	0.83519478
IL11RA	NM_001142784	0.83495821
MRNIP	NM_016175	0.83451619
SEPTIN1	NM_001365977	0.83437917
TMEM120B	NM_001080825	0.83376034
ZNF853	NM_001353546	0.83355388
LEF1	NM_016269	0.83287052
SYNGAP1	NM_006772	0.83253547

RTL10	NM_024627	0.83070213
RUNX3	NM_004350	0.83053318
ZNF696	NM_030895	0.83050847
DTX1	NM_004416	0.83002592
SMIM10L2A	NM_203306	0.82894659
NUP210	NM_024923	0.82882989
APOBEC3D	NM_152426	0.82839216
GPA33	NM_005814	0.82748635
PCED1B-AS1	NR_026544	0.82694882
RRP1B	NM_015056	0.82647008
MICAL3	NM_015241	0.82620419
MSTO2P	NR_024117.1	0.82598293
RHOH	NM_001278363	0.82443655
STXBP1	NM_001032221	0.82443523
LOC728743	NR_027237	0.82383506
C12orf65	NM_152269	0.82321817
PHC1	NM_004426	0.82286191
CHST12	NM_001243794	0.8213658
OLFM2	NM_001304348	0.82099062
NFATC2IP	NM_032815	0.82060608
RFTN1	NM_015150	0.82040236
UBE2G2	NM_003343	0.82032575
SPN	NM_003123	0.82027907
SMPD3	NM_018667	0.81979431
ZSWIM5	NM_020883	0.81965432
HPCAL4	NM_016257	0.8190551
LIG3	NM_013975	0.81901648
ASB2	NM_016150	0.81836591
ZNF275	NM_001367757	0.81811537
CHST12	NM_018641	0.81802732
CHST12	NM_001243795	0.81802659
SLC20A2	NM_001257181	0.81785533
ID3	NM_002167	0.81757936
ATG9B	NR_073169	0.8168441
BCL9L	NM_182557	0.81653765
NLRC3	NR_075083	0.8164838
ADAMTS17	NM_139057	0.81600048
HEMK1	NM_016173	0.8158972

NFATC2	NM_001136021	0.81583202
RASA3	NM_007368	0.81532218
POLR1A	NM_015425	0.81342511
TNFAIP8L1	NM_152362	0.81320085
SIGLEC8	NM_014442	0.81141094
CCR7	NM_001301718	0.81125876
EP400	NM_015409	0.81092975
SLC25A4	NM_001151	0.81047735
PASK	NM_001252120	0.81044314
PCED1B	NM_138371	0.81008092
NKD1	NM_033119	0.80990999
BICDL1	NM_207311	0.80921025
WNT10B	NM_003394	0.80797639
EPHA1	NM_005232	0.80758535
SORBS3	NM_005775	0.80740121
GDF11	NM_005811	0.8073361
ESYT1	NM_001184796	0.80567105
CEACAM21	NM_001288773	0.80547949
NOP14	NM_001291978	0.80543659
DGKA	NM_001345	0.80474746
HKDC1	NM_025130	0.80340809
FOXP3	NM_014009	0.80263408
CYB561	NM_001915	0.80167343
EARS2	NR_003501	0.80092487
BICDL1	NR_147894	0.79966571

Supplementary Table S2: Figure 4A gene list				
**Gene symbol**	**Transcript ID**	**P-value (AB vs. CD)**	**Fold change (AB vs. CD)**	**FDR step up (AB vs. CD)**
A1BG-AS1	NR_015380	1.60E-05	1.95E+00	1.31E-03
AAAS	NM_015665	5.13E-03	1.69E+00	3.76E-02
AASDH	NM_181806	3.86E-03	-1.89E+00	3.15E-02
AASDHPPT	NM_015423	2.72E-04	-1.50E+00	6.69E-03
AASS	NM_005763	3.89E-04	-1.79E+00	8.16E-03
ABCA5	NM_018672	3.17E-03	-1.99E+00	2.80E-02
ABCB1	NM_001348944	1.06E-03	-3.01E+00	1.45E-02
ABCB8	NM_001282293	2.35E-03	2.76E+00	2.36E-02
ABCC10	NR_146762	1.27E-03	2.79E+00	1.62E-02
ABCC4	NM_005845	4.04E-03	-1.79E+00	3.23E-02
ABCD4	NM_001353601	2.18E-04	2.98E+00	5.92E-03
ABCF1	NM_001090	5.34E-03	2.23E+00	3.86E-02
ABCG2	NM_001348985	2.93E-03	-2.72E+00	2.68E-02
ABHD10	NR_073571	7.06E-03	1.74E+00	4.58E-02
ABHD11	NM_001321383	1.78E-03	2.04E+00	2.01E-02
ABHD16A	NR_033489	1.24E-03	2.59E+00	1.60E-02
ABHD17C	NM_021214	1.88E-04	-1.72E+00	5.44E-03
ABHD18	NM_001366043	6.03E-03	-2.61E+00	4.14E-02
ABTB1	NR_033429	1.93E-03	2.49E+00	2.09E-02
ACAA1	NM_001130410	6.63E-06	3.37E+00	8.39E-04
ACADSB	NM_001609	2.69E-04	-1.74E+00	6.66E-03
ACADVL	NM_000018	1.51E-04	1.87E+00	4.75E-03
ACBD4	NM_001135707	1.32E-04	2.18E+00	4.39E-03
ACBD5	NM_145698	3.05E-04	-1.84E+00	7.14E-03
ACD	NM_022914	4.59E-04	2.89E+00	8.92E-03
ACER2	NM_001010887	4.71E-04	-2.19E+00	9.04E-03
ACIN1	NM_001164817	1.09E-03	2.21E+00	1.47E-02
ACSL3	NM_203372	2.18E-04	-1.84E+00	5.92E-03
ACSL4	NM_001318509	9.17E-04	-2.82E+00	1.33E-02
ACTN1	NM_001130005	2.43E-03	-2.10E+00	2.41E-02
ACTR2	NM_005722	2.09E-05	-1.66E+00	1.56E-03
ACTR3B	NM_001350946	6.49E-03	-4.71E+00	4.35E-02
ADA	NR_136160	1.60E-03	2.52E+00	1.87E-02
ADA2	NM_177405	1.24E-03	1.96E+00	1.60E-02

ADAM10	NM_001110	2.80E-04	-1.65E+00	6.80E-03
ADAM22	NM_001324417	7.87E-03	-1.91E+00	4.90E-02
ADAMTSL4	NM_001288607	5.16E-04	1.95E+00	9.49E-03
ADAP1	NM_001284310	5.53E-04	3.76E+00	9.87E-03
ADAT1	NM_001324449	3.79E-04	-2.09E+00	8.04E-03
ADCY4	NM_139247	8.23E-04	1.81E+00	1.24E-02
ADD2	NM_017488	4.02E-03	-1.85E+00	3.22E-02
ADD3	NM_016824	2.77E-03	-1.58E+00	2.60E-02
ADGRG3	NM_001308360	4.24E-03	4.48E+00	3.32E-02
ADNP	NM_181442	2.85E-05	-1.61E+00	1.85E-03
ADORA2A	NR_103544	5.41E-03	2.08E+00	3.90E-02
ADORA2B	NM_000676	6.67E-03	2.05E+00	4.42E-02
AEBP2	NM_153207	1.07E-03	-1.90E+00	1.46E-02
AFF1	NM_001166693	1.24E-05	-2.18E+00	1.16E-03
AFF3	NM_002285	2.83E-04	-2.31E+00	6.81E-03
AFF4	NM_014423	1.11E-06	-1.88E+00	4.12E-04
AGAP3	NM_001281300	7.57E-03	1.75E+00	4.79E-02
AGAP9	NM_001190810	2.44E-03	1.98E+00	2.41E-02
AGER	NR_038190	3.55E-05	2.23E+00	2.10E-03
AGL	NM_000642	3.59E-03	-1.69E+00	3.01E-02
AGTPBP1	NM_001330701	3.36E-03	-1.69E+00	2.89E-02
AGTRAP	NM_001040195	4.18E-03	3.57E+00	3.30E-02
AHCTF1	NM_001323343	5.07E-04	-2.22E+00	9.39E-03
AHCTF1P1	NR_077058	6.50E-05	-2.36E+00	2.83E-03
AHNAK	NM_024060	8.15E-05	2.03E+00	3.30E-03
AIF1	NM_032955	3.81E-03	1.51E+00	3.12E-02
AK3	NM_001199856	3.55E-03	-1.88E+00	2.99E-02
AKAP11	NM_016248	2.72E-03	-1.52E+00	2.57E-02
AKAP13	NM_006738	2.90E-07	-2.96E+00	2.75E-04
AKAP17A	NM_005088	3.05E-03	1.52E+00	2.73E-02
AKAP5	NM_004857	1.56E-04	-2.99E+00	4.82E-03
AKAP8L	NR_111971	2.05E-04	2.42E+00	5.71E-03
AKAP9	NM_005751	2.53E-05	-1.71E+00	1.73E-03
AKR1B1	NR_144376	3.13E-03	1.67E+00	2.77E-02
AKR1C1	NM_001353	4.19E-03	2.28E+00	3.30E-02
AKT1S1	NM_001278160	1.98E-03	2.93E+00	2.12E-02
ALG10B	NM_001013620	4.94E-04	-1.93E+00	9.25E-03
ALG11	NM_001004127	3.07E-04	-1.68E+00	7.17E-03

ALG9	NM_001352419	2.24E-03	-3.12E+00	2.28E-02
ALKBH2	NM_001145374	5.89E-04	2.00E+00	1.02E-02
ALKBH8	NM_138775	2.93E-03	-1.67E+00	2.68E-02
ALMS1	NM_015120	1.07E-03	-1.69E+00	1.46E-02
ALPL	NM_001177520	4.06E-03	4.05E+00	3.23E-02
ALS2CL	NR_135622	1.66E-03	2.31E+00	1.92E-02
AMD1	NM_001287214	9.90E-05	-1.97E+00	3.76E-03
AMMECR1	NM_015365	1.33E-04	-1.81E+00	4.42E-03
AMPD2	NM_001257361	9.81E-05	1.94E+00	3.74E-03
AMT	NM_001164710	6.17E-04	2.73E+00	1.05E-02
ANAPC11	NM_016476	4.15E-03	2.99E+00	3.29E-02
ANAPC15	NM_001330321	4.81E-05	1.81E+00	2.42E-03
ANGPT1	NM_001199859	2.69E-04	-3.11E+00	6.66E-03
ANKIB1	NM_019004	2.35E-04	-1.52E+00	6.18E-03
ANKRD12	NM_001083625	3.39E-04	-1.76E+00	7.58E-03
ANKRD17	NM_015574	2.35E-04	-1.99E+00	6.18E-03
ANKRD26	NM_014915	4.72E-03	-1.64E+00	3.56E-02
ANKRD28	NM_001349283	9.83E-05	-5.66E+00	3.75E-03
ANKRD44	NM_001367497	5.78E-06	-2.20E+00	8.21E-04
ANO6	NM_001025356	2.25E-05	-1.66E+00	1.62E-03
ANO9	NM_001012302	6.42E-03	2.29E+00	4.31E-02
ANP32E	NM_030920	4.12E-04	-1.60E+00	8.35E-03
ANXA2R	NM_001014279	2.29E-03	1.99E+00	2.32E-02
AP1G2	NM_001354673	4.38E-06	5.58E+00	7.15E-04
AP1S1	NM_001283	5.81E-04	1.78E+00	1.01E-02
AP1S2	NM_001369008	2.92E-03	-2.64E+00	2.67E-02
AP1S3	NM_001039569	7.97E-03	-2.00E+00	4.95E-02
AP2S1	NM_004069	2.37E-03	1.61E+00	2.36E-02
AP4B1	NM_001308312	1.45E-05	3.22E+00	1.26E-03
AP4M1	NM_001363671	1.24E-03	1.68E+00	1.60E-02
APAF1	NM_181868	4.06E-05	-2.35E+00	2.21E-03
APBB3	NM_006051	1.23E-05	1.99E+00	1.15E-03
APC	NM_001354896	7.56E-07	-2.44E+00	3.69E-04
APEX1	NM_001641	3.98E-03	1.58E+00	3.20E-02
APH1A	NR_045033	4.15E-04	1.63E+00	8.37E-03
API5	NM_006595	6.70E-04	-2.08E+00	1.10E-02
APLP2	NR_024516	7.72E-04	2.69E+00	1.20E-02
APOBEC3A	NM_001270406	7.55E-03	3.68E+00	4.79E-02

APOBEC3B	NM_004900	5.28E-04	3.94E+00	9.63E-03
APOM	NM_001256169	1.24E-03	2.06E+00	1.60E-02
APP	NM_001204301	2.05E-03	-1.71E+00	2.16E-02
APPBP2	NM_006380	4.73E-04	-1.50E+00	9.05E-03
APTX	NM_001370669	6.18E-04	2.51E+00	1.05E-02
ARAP2	NM_015230	1.11E-07	-2.37E+00	1.62E-04
ARCN1	NM_001655	4.87E-04	-1.73E+00	9.19E-03
ARF5	NM_001662	3.22E-03	1.79E+00	2.82E-02
ARFGEF1	NM_006421	3.00E-05	-1.55E+00	1.92E-03
ARFGEF2	NM_006420	2.83E-05	-1.61E+00	1.85E-03
ARFIP2	NM_001370408	6.10E-05	2.12E+00	2.74E-03
ARHGAP12	NM_001270696	4.56E-03	-1.85E+00	3.48E-02
ARHGAP21	NM_001367451	1.17E-04	-3.51E+00	4.10E-03
ARHGAP42	NM_152432	1.02E-03	-1.86E+00	1.41E-02
ARHGAP5	NM_001030055	2.46E-03	-1.75E+00	2.42E-02
ARHGAP9	NM_001319851	3.02E-07	2.58E+00	2.75E-04
ARHGEF1	NM_198977	7.65E-03	3.21E+00	4.82E-02
ARHGEF12	NM_001301084	1.24E-03	-1.76E+00	1.60E-02
ARHGEF17	NM_014786	9.48E-06	4.49E+00	1.02E-03
ARHGEF3	NM_019555	2.55E-04	-1.93E+00	6.52E-03
ARHGEF7	NM_001354055	5.13E-04	2.19E+00	9.46E-03
ARHGEF9	NM_001369040	4.30E-03	-2.19E+00	3.35E-02
ARID2	NM_152641	1.03E-05	-1.53E+00	1.05E-03
ARID4A	NM_002892	2.82E-04	-1.67E+00	6.80E-03
ARID4B	NM_016374	1.31E-05	-1.99E+00	1.20E-03
ARID5B	NM_032199	4.15E-06	-2.58E+00	7.03E-04
ARIH2	NM_001349221	1.06E-06	2.66E+00	4.02E-04
ARL13B	NM_001174150	1.76E-03	-2.75E+00	2.00E-02
ARL16	NM_001329608	1.47E-05	1.53E+00	1.27E-03
ARL6IP4	NM_001002252	2.64E-03	3.06E+00	2.53E-02
ARMC12	NM_001286574	1.53E-03	2.10E+00	1.81E-02
ARMC8	NM_001363941	5.22E-03	-1.51E+00	3.80E-02
ARMCX6	NR_033669	1.36E-03	1.54E+00	1.69E-02
ARMH1	NM_001145636	5.86E-05	2.99E+00	2.68E-03
ARNTL	NR_147788	1.13E-04	-2.86E+00	4.04E-03
ARPC4	NM_001024959	5.73E-03	1.92E+00	4.03E-02
ARPC4-TTLL3	NM_001198793	3.09E-03	2.31E+00	2.75E-02
ARPIN	NM_182616	3.31E-04	2.59E+00	7.49E-03

ARPIN-AP3S2	NM_001199058	4.90E-07	8.02E+00	3.28E-04
ARRDC1-AS1	NR_122036	8.08E-03	2.39E+00	4.99E-02
ARRDC2	NM_001025604	5.36E-03	1.74E+00	3.87E-02
ARRDC3	NM_020801	4.96E-04	-1.97E+00	9.25E-03
ARRDC5	NM_001367189	7.28E-03	3.23E+00	4.68E-02
ASAP1-IT2	NR_045385	2.82E-03	1.90E+00	2.62E-02
ASAP2	NM_003887	7.15E-05	-2.17E+00	3.02E-03
ASB6	NM_177999	2.84E-04	1.53E+00	6.82E-03
ASCC3	NM_006828	1.10E-04	-1.61E+00	3.99E-03
ASGR1	NM_001197216	7.39E-03	2.25E+00	4.73E-02
ASGR2	NM_001201352	1.31E-03	3.31E+00	1.65E-02
ASH1L	NM_001366177	1.37E-06	-1.66E+00	4.50E-04
ASMTL-AS1	NR_026710.1	6.09E-05	2.80E+00	2.74E-03
ASPM	NM_018136	1.90E-03	-2.51E+00	2.08E-02
ASXL2	NM_018263	4.03E-08	-2.03E+00	1.23E-04
ATAD1	NM_032810	2.26E-04	-2.99E+00	6.03E-03
ATAD2	NM_014109	1.55E-04	-1.68E+00	4.82E-03
ATAD2B	NM_017552	2.36E-03	-1.70E+00	2.36E-02
ATAD5	NM_024857	4.75E-03	-1.60E+00	3.58E-02
ATE1	NM_007041	3.45E-05	-1.88E+00	2.08E-03
ATF2	NM_001880	3.92E-05	-2.13E+00	2.19E-03
ATF4	NM_182810	4.47E-06	1.54E+00	7.15E-04
ATF7IP	NM_018179	1.16E-05	-1.53E+00	1.11E-03
ATG13	NM_001205122	2.08E-03	2.88E+00	2.18E-02
ATG16L2	NM_033388	6.87E-03	1.83E+00	4.50E-02
ATG9A	NR_104255	6.01E-03	1.60E+00	4.14E-02
ATG9B	NR_073169	4.10E-03	1.77E+00	3.26E-02
ATL2	NM_022374	1.56E-04	-1.91E+00	4.82E-03
ATM	NM_001351834	4.60E-04	-1.70E+00	8.92E-03
ATOX1	NM_004045	7.93E-04	1.70E+00	1.21E-02
ATP10D	NM_020453	2.62E-06	-1.64E+00	5.85E-04
ATP13A4	NM_032279	1.42E-05	-2.21E+00	1.25E-03
ATP2A2	NM_001681	3.58E-04	-6.76E+00	7.79E-03
ATP2B1	NM_001366525	5.71E-04	-2.05E+00	1.00E-02
ATP2B4	NM_001684	1.40E-04	-1.54E+00	4.53E-03
ATP5F1E	NM_006886	1.18E-03	1.94E+00	1.54E-02
ATP5MC1	NM_001002027	1.60E-05	1.68E+00	1.31E-03
ATP5MC2	NR_163135	1.63E-03	1.74E+00	1.89E-02

ATP5MD	NM_001206427	1.09E-04	1.72E+00	3.98E-03
ATP5ME	NM_007100	5.07E-03	2.22E+00	3.74E-02
ATP5MF	NM_004889	2.23E-05	2.03E+00	1.61E-03
ATP5MG	NM_006476	1.63E-04	1.78E+00	4.97E-03
ATP5PF	NM_001003703	1.32E-03	1.74E+00	1.66E-02
ATP5PO	NM_001697	8.61E-04	1.51E+00	1.28E-02
ATP6V0B	NM_001294333	1.57E-05	1.81E+00	1.31E-03
ATP6V1A	NM_001690	3.68E-04	-1.58E+00	7.93E-03
ATP6V1F	NM_004231	5.47E-04	1.91E+00	9.82E-03
ATP7A	NM_001282224	2.22E-04	-2.17E+00	5.95E-03
ATP8A1	NM_001105529	3.32E-04	-1.70E+00	7.50E-03
ATPAF2	NM_145691	5.61E-03	1.51E+00	3.98E-02
ATR	NM_001184	5.72E-04	-2.04E+00	1.00E-02
ATRAID	NM_016085	7.54E-03	2.29E+00	4.78E-02
ATRX	NM_000489	2.23E-07	-1.95E+00	2.38E-04
ATXN1L	NM_001137675	1.66E-05	-1.51E+00	1.35E-03
ATXN2	NM_001310121	3.11E-03	-1.54E+00	2.76E-02
ATXN2L	NM_148415	3.85E-03	-1.69E+00	3.14E-02
ATXN7	NM_001128149	1.25E-08	-1.85E+00	5.24E-05
AUP1	NR_126510	2.56E-04	1.88E+00	6.52E-03
AUTS2	NM_015570	5.11E-04	-2.28E+00	9.44E-03
AZIN1	NM_015878	3.65E-05	-2.46E+00	2.13E-03
B3GLCT	NM_194318	5.97E-04	-1.58E+00	1.03E-02
B3GNT9	NM_033309	9.59E-04	1.59E+00	1.36E-02
B3GNTL1	NM_001009905	2.05E-03	1.68E+00	2.16E-02
B4GALT4	NM_212543	6.15E-03	-2.13E+00	4.20E-02
B4GALT6	NM_004775	5.63E-03	-1.92E+00	3.99E-02
BABAM1	NM_014173	3.28E-03	1.74E+00	2.85E-02
BACH1	NM_206866	6.45E-05	-1.79E+00	2.83E-03
BANF1	NM_003860	3.17E-04	1.53E+00	7.30E-03
BANK1	NM_017935	6.67E-04	-2.87E+00	1.10E-02
BARD1	NM_001282543	9.29E-04	-1.94E+00	1.34E-02
BATF3	NM_018664	7.32E-03	2.15E+00	4.69E-02
BAZ1A	NM_182648	3.74E-03	-1.62E+00	3.08E-02
BAZ1B	NM_032408	6.39E-03	-1.70E+00	4.30E-02
BAZ2B	NM_013450	1.09E-03	-1.83E+00	1.47E-02
BBS10	NM_024685	3.22E-03	-1.56E+00	2.82E-02
BBS9	NM_001348046	9.38E-04	2.22E+00	1.35E-02

BBX	NM_020235	1.57E-06	-2.60E+00	4.73E-04
BCAP29	NM_018844	4.64E-04	-1.73E+00	8.95E-03
BCL2L11	NM_138624	4.27E-03	-1.73E+00	3.33E-02
BCL7B	NM_001707	2.92E-03	1.55E+00	2.67E-02
BCLAF1	NM_014739	1.79E-05	-1.98E+00	1.41E-03
BCOR	NM_001123385	3.10E-03	-2.01E+00	2.75E-02
BDP1	NM_018429	5.29E-04	-1.83E+00	9.63E-03
BEGAIN	NM_020836	3.02E-03	3.41E+00	2.72E-02
BEND4	NM_207406	6.05E-04	-2.32E+00	1.04E-02
BET1L	NM_016526	2.59E-03	1.70E+00	2.51E-02
BEX2	NM_032621	1.83E-03	3.26E+00	2.03E-02
BEX3	NM_014380	1.58E-03	2.42E+00	1.86E-02
BEX5	NM_001012978	3.38E-03	1.71E+00	2.90E-02
BHLHE41	NM_030762	4.31E-04	-2.45E+00	8.59E-03
BICRAL	NM_015349	1.28E-05	-1.50E+00	1.18E-03
BIRC3	NM_001165	5.64E-04	-2.20E+00	9.98E-03
BIRC6	NM_016252	3.52E-06	-1.63E+00	6.72E-04
BLM	NM_000057	2.04E-03	-1.82E+00	2.16E-02
BLOC1S1	NM_001487	1.25E-03	2.48E+00	1.60E-02
BLOC1S5-TXNDC5	NR_037616	1.06E-05	-2.20E+00	1.06E-03
BLOC1S6	NM_001311255	2.32E-04	-2.80E+00	6.14E-03
BLZF1	NM_001320972	3.38E-03	-1.87E+00	2.90E-02
BMP2K	NM_198892	1.46E-03	-1.59E+00	1.77E-02
BMP6	NM_001718	5.22E-03	-1.59E+00	3.80E-02
BMPR1A	NM_004329	5.74E-03	-1.61E+00	4.03E-02
BNIPL	NM_138278	9.02E-04	3.03E+00	1.31E-02
BOD1L1	NM_148894	2.01E-06	-1.68E+00	5.14E-04
BOLA1	NM_016074	1.16E-04	1.67E+00	4.07E-03
BORCS7-ASMT	NR_037644	7.42E-04	2.26E+00	1.17E-02
BPHL	NM_004332	2.81E-03	1.51E+00	2.61E-02
BPTF	NM_004459	6.89E-07	-1.83E+00	3.69E-04
BRCA1	NM_007294	4.28E-07	-3.04E+00	3.14E-04
BRCA2	NM_000059	4.47E-03	-1.70E+00	3.44E-02
BRIP1	NM_032043	4.94E-03	-1.60E+00	3.68E-02
BROX	NM_144695	3.17E-05	-2.67E+00	1.97E-03
BRWD1	NM_033656	1.46E-03	-1.57E+00	1.77E-02
BRWD3	NM_153252	4.32E-05	-1.69E+00	2.27E-03

BTBD1	NM_025238	1.22E-04	-1.52E+00	4.20E-03
BTBD6	NM_033271	1.07E-03	1.82E+00	1.46E-02
BTBD7	NM_001002860	4.81E-04	-2.26E+00	9.15E-03
BTBD9	NM_052893	1.81E-03	-1.81E+00	2.02E-02
BTD	NM_001370658	1.63E-04	-1.55E+00	4.96E-03
BTN3A1	NM_001145009	4.79E-04	-1.55E+00	9.11E-03
BTN3A2	NM_001197248	2.70E-03	1.86E+00	2.56E-02
BZW1	NM_014670	3.80E-06	-1.74E+00	7.03E-04
C10orf143	NM_001355042	4.91E-03	1.58E+00	3.66E-02
C12orf10	NM_021640	4.33E-03	1.86E+00	3.36E-02
C16orf70	NM_001320543	2.53E-03	3.00E+00	2.46E-02
C16orf87	NM_001001436	3.56E-03	-1.53E+00	3.00E-02
C17orf49	NM_174893	7.03E-04	1.81E+00	1.13E-02
C17orf80	NR_110105	4.72E-04	2.58E+00	9.05E-03
C18orf21	NM_001201475	2.00E-03	1.94E+00	2.13E-02
C18orf25	NM_145055	5.13E-04	-2.36E+00	9.46E-03
C18orf54	NM_001288981	3.13E-03	-2.91E+00	2.77E-02
C19orf38	NM_001136482	2.42E-03	1.95E+00	2.40E-02
C19orf44	NM_032207	2.90E-03	1.84E+00	2.66E-02
C19orf53	NM_014047	1.32E-03	2.31E+00	1.66E-02
C19orf54	NM_001353807	5.41E-03	7.08E+00	3.90E-02
C1GALT1	NM_020156	2.48E-03	-1.62E+00	2.43E-02
C1orf122	NM_001142726	1.55E-05	2.17E+00	1.30E-03
C1orf21	NM_030806	4.75E-04	-1.85E+00	9.07E-03
C1orf43	NM_001297721	5.42E-04	1.86E+00	9.79E-03
C1orf50	NM_024097	2.08E-05	1.52E+00	1.56E-03
C1QTNF6	NM_031910	1.53E-05	2.59E+00	1.30E-03
C1R	NM_001733	5.67E-04	1.77E+00	1.00E-02
C20orf204	NM_001348090	2.39E-03	1.81E+00	2.38E-02
C21orf91	NM_017447	4.16E-04	-2.06E+00	8.37E-03
C22orf34	NM_001289922	2.66E-06	2.03E+00	5.85E-04
C2CD2L	NM_014807	7.48E-03	1.62E+00	4.76E-02
C2CD5	NM_014802	7.25E-06	-1.97E+00	8.72E-04
C2orf42	NM_001348758	8.74E-04	-2.22E+00	1.29E-02
C2orf69	NM_153689	3.71E-04	-1.74E+00	7.93E-03
C4A	NM_007293	8.37E-04	3.39E+00	1.26E-02
C4B	NM_001002029	1.15E-03	3.33E+00	1.52E-02
C4B_2	NM_001242823.1	1.15E-03	3.33E+00	1.52E-02

C5	NM_001735	6.26E-03	-1.59E+00	4.24E-02
C5orf22	NM_018356	4.64E-03	-1.55E+00	3.53E-02
C5orf24	NM_001135586	4.04E-03	-1.57E+00	3.23E-02
C5orf30	NM_001316969	7.00E-03	-3.76E+00	4.55E-02
C5orf67	NR_161255	5.18E-03	1.90E+00	3.78E-02
C7orf31	NM_001371351	7.24E-03	-2.10E+00	4.66E-02
C7orf61	NM_001004323	3.93E-03	2.39E+00	3.18E-02
CA2	NM_000067	2.46E-04	-2.18E+00	6.36E-03
CACNA1C-AS2	NR_046579	4.71E-03	1.61E+00	3.56E-02
CACNA1H	NM_001005407	7.14E-03	2.81E+00	4.62E-02
CACNA1I	NM_021096	6.58E-03	2.32E+00	4.38E-02
CACNB3	NM_001206917	6.68E-03	1.63E+00	4.42E-02
CALCRL	NM_001369434	2.61E-05	-5.05E+00	1.76E-03
CALD1	NM_004342	8.05E-03	-2.97E+00	4.98E-02
CALM2	NM_001305626	4.29E-03	-1.75E+00	3.34E-02
CALU	NM_001130674	2.16E-03	-1.73E+00	2.23E-02
CAMK1	NM_003656	2.76E-04	1.73E+00	6.75E-03
CAMKK1	NM_172206	2.22E-03	1.80E+00	2.27E-02
CAMSAP2	NM_203459	1.01E-03	-1.75E+00	1.41E-02
CANX	NM_001746	1.23E-07	-1.78E+00	1.63E-04
CAPN10-DT	NR_103792	1.02E-05	1.54E+00	1.05E-03
CAPN3	NM_173090	7.87E-06	1.98E+00	9.10E-04
CAPRIN2	NM_001319842	3.05E-03	-1.97E+00	2.73E-02
CARD19	NR_134465	3.91E-03	2.24E+00	3.17E-02
CARNMT1	NM_152420	2.51E-03	-1.98E+00	2.45E-02
CARS2	NR_147942	1.22E-03	1.62E+00	1.58E-02
CASC4	NR_157849	1.00E-03	-1.86E+00	1.40E-02
CASD1	NM_001363427	1.08E-04	4.71E+00	3.98E-03
CASP8AP2	NM_001137667	1.09E-04	-2.17E+00	3.98E-03
CAST	NM_001330632	3.93E-03	-1.83E+00	3.18E-02
CATSPER1	NM_053054	9.99E-04	2.08E+00	1.40E-02
CAVIN2	NM_004657	5.86E-04	-1.97E+00	1.02E-02
CAVIN3	NM_145040	7.74E-07	5.52E+00	3.69E-04
CBFB	NM_001755	1.52E-03	-1.67E+00	1.81E-02
CBLB	NM_001321793	8.09E-04	-2.12E+00	1.23E-02
CBLL1	NM_024814	7.61E-05	-1.51E+00	3.15E-03
CBR1	NM_001286789	1.13E-06	1.83E+00	4.12E-04
CBR3	NM_001236	3.16E-03	2.08E+00	2.79E-02

CCDC107	NM_174923	3.47E-04	1.94E+00	7.70E-03
CCDC117	NM_001284265	4.26E-04	-1.59E+00	8.52E-03
CCDC125	NM_001297697	5.33E-03	-1.62E+00	3.86E-02
CCDC142	NM_032779	2.82E-03	1.71E+00	2.62E-02
CCDC159	NM_001080503	1.21E-03	1.66E+00	1.57E-02
CCDC167	NM_138493	7.23E-04	2.65E+00	1.16E-02
CCDC186	NM_001321829	4.51E-05	-1.88E+00	2.32E-03
CCDC84	NR_104049	6.87E-05	1.75E+00	2.96E-03
CCDC90B	NM_001286116	4.28E-03	-3.83E+00	3.34E-02
CCDC91	NM_018318	7.57E-03	-1.70E+00	4.79E-02
CCNB1IP1	NM_182849	2.06E-04	2.83E+00	5.72E-03
CCNG2	NM_004354	5.42E-05	-1.82E+00	2.60E-03
CCNL2	NM_001350497	8.04E-03	4.55E+00	4.98E-02
CCNT1	NM_001240	2.71E-06	-1.77E+00	5.85E-04
CCP110	NM_001323569	4.18E-03	-1.81E+00	3.30E-02
CCR6	NM_031409	5.58E-05	-2.67E+00	2.62E-03
CCR7	NM_001838	6.80E-03	1.93E+00	4.47E-02
CCSER2	NM_018999	6.17E-06	-2.35E+00	8.32E-04
CD164	NM_001142403	4.50E-03	7.81E+00	3.45E-02
CD200	NM_005944	5.90E-03	-2.13E+00	4.09E-02
CD226	NM_001303618	2.04E-03	-2.21E+00	2.16E-02
CD24	NM_001359084	3.50E-03	-1.63E+00	2.97E-02
CD248	NM_020404	6.76E-03	2.75E+00	4.45E-02
CD300LB	NM_174892	1.95E-04	1.52E+00	5.56E-03
CD300LF	NM_139018	2.29E-03	1.53E+00	2.32E-02
CD33	NM_001772	3.09E-05	1.78E+00	1.96E-03
CD36	NM_001371077	2.05E-03	1.91E+00	2.16E-02
CD38	NM_001775	7.39E-06	-2.20E+00	8.73E-04
CD3D	NM_000732	2.33E-03	1.88E+00	2.34E-02
CD4	NM_001195014	6.22E-04	1.86E+00	1.06E-02
CD46	NM_172352	1.99E-03	-1.87E+00	2.13E-02
CD48	NM_001778	1.03E-03	1.56E+00	1.43E-02
CD5	NM_001346456	3.66E-03	1.90E+00	3.05E-02
CD52	NM_001803	5.25E-05	2.49E+00	2.57E-03
CD68	NM_001040059	3.70E-03	1.82E+00	3.07E-02
CD84	NM_003874	6.55E-04	-1.62E+00	1.09E-02
CD8A	NM_001768	3.22E-03	2.17E+00	2.82E-02
CD8B	NM_172101	1.02E-04	3.40E+00	3.81E-03

CD99	NM_002414	5.48E-04	1.57E+00	9.83E-03
CDA	NM_001785	3.46E-03	1.76E+00	2.95E-02
CDC14A	NM_003672	3.53E-04	-1.54E+00	7.75E-03
CDC14B	NM_001077181	4.65E-04	-1.97E+00	8.96E-03
CDC25B	NR_136335	4.33E-03	1.93E+00	3.36E-02
CDC27	NM_001293089	1.57E-03	-1.95E+00	1.85E-02
CDC42	NM_044472	4.07E-03	-1.79E+00	3.24E-02
CDC42SE2	NM_020240	1.77E-05	-1.75E+00	1.41E-03
CDC7	NM_001134420	3.20E-03	-1.82E+00	2.81E-02
CDCA7L	NM_018719	1.82E-04	-2.21E+00	5.31E-03
CDH23	NM_001171931	6.72E-04	2.32E+00	1.10E-02
CDIP1	NM_001199055	1.66E-03	1.91E+00	1.92E-02
CDIPT	NM_001286586	4.85E-04	1.86E+00	9.19E-03
CDK14	NM_001287136	1.98E-03	-1.66E+00	2.12E-02
CDK17	NM_001170464	2.12E-05	-2.00E+00	1.57E-03
CDK19	NM_015076	1.56E-06	-2.79E+00	4.73E-04
CDK3	NM_001258	2.71E-06	2.12E+00	5.85E-04
CDK5	NM_004935	4.73E-03	1.61E+00	3.57E-02
CDK5RAP1	NM_001278169	1.32E-05	2.16E+00	1.20E-03
CDK5RAP2	NM_001272039	7.59E-03	4.04E+00	4.80E-02
CDK5RAP3	NM_001278216	9.80E-04	1.79E+00	1.38E-02
CDKL5	NM_001323289	8.59E-04	-1.57E+00	1.28E-02
CDPF1	NM_207327	4.03E-03	1.80E+00	3.23E-02
CDV3	NM_001134423	6.45E-05	-2.36E+00	2.83E-03
CEACAM3	NR_102333	4.03E-05	1.89E+00	2.21E-03
CEBPA	NM_001287424	5.99E-03	1.65E+00	4.13E-02
CEBPA-DT	NR_026887	5.64E-04	1.68E+00	9.97E-03
CEBPZOS	NM_001322375	2.85E-03	2.86E+00	2.63E-02
CELF1	NM_001330272	9.00E-04	-1.68E+00	1.31E-02
CELF2	NM_001326349	1.60E-06	-6.02E+00	4.73E-04
CEMIP2	NM_013390	3.78E-04	-1.78E+00	8.02E-03
CENPC	NM_001812	7.68E-04	-1.65E+00	1.19E-02
CENPJ	NM_018451	3.72E-03	-1.63E+00	3.08E-02
CENPT	NM_025082	2.10E-03	1.85E+00	2.20E-02
CENPV	NM_181716	1.52E-03	1.62E+00	1.81E-02
CEP120	NM_153223	5.85E-06	-1.84E+00	8.22E-04
CEP128	NM_152446	5.09E-03	-1.51E+00	3.75E-02
CEP170	NM_014812	5.44E-05	-1.85E+00	2.61E-03

CEP192	NM_032142	8.12E-05	-1.65E+00	3.30E-03
CEP295	NM_033395	9.54E-04	-1.54E+00	1.36E-02
CEP350	NM_014810	7.14E-07	-1.74E+00	3.69E-04
CEP76	NM_024899	6.89E-03	-1.58E+00	4.51E-02
CEP78	NM_001349839	5.40E-03	-1.82E+00	3.89E-02
CEP85L	NM_001178035	1.93E-03	-1.71E+00	2.10E-02
CEP97	NM_024548	3.11E-04	-1.94E+00	7.22E-03
CERT1	NM_005713	2.72E-03	1.69E+00	2.57E-02
CES2	NM_001365407	4.93E-04	2.50E+00	9.24E-03
CES4A	NM_001190202	4.13E-04	2.07E+00	8.35E-03
CFAP97	NM_020827	3.31E-03	-1.67E+00	2.86E-02
CFD	NM_001317335	5.82E-03	1.94E+00	4.06E-02
CHAMP1	NM_032436	2.30E-03	-1.68E+00	2.33E-02
CHCHD1	NM_203298	2.18E-03	1.56E+00	2.25E-02
CHCHD2	NM_001320327	2.82E-04	1.91E+00	6.80E-03
CHCHD3	NM_001317177	1.45E-03	-2.39E+00	1.76E-02
CHD1	NM_001270	2.06E-04	-1.58E+00	5.72E-03
CHD1L	NR_145687	1.54E-04	2.00E+00	4.80E-03
CHD6	NM_032221	2.57E-04	-1.55E+00	6.54E-03
CHD7	NM_017780	1.89E-04	-1.85E+00	5.46E-03
CHD8	NM_001170629	1.61E-03	-2.24E+00	1.88E-02
CHD9	NM_001308319	3.09E-04	-4.22E+00	7.19E-03
CHI3L2	NM_004000	2.75E-04	2.63E+00	6.73E-03
CHIC1	NM_001039840	1.06E-03	-2.01E+00	1.45E-02
CHKB	NM_005198	3.97E-05	1.54E+00	2.20E-03
CHN2	NM_004067	5.63E-03	-3.26E+00	3.99E-02
CHRNE	NM_000080	4.01E-03	1.83E+00	3.22E-02
CHURC1	NM_145165	4.87E-04	-1.73E+00	9.19E-03
CIAO2B	NM_016062	3.32E-03	2.11E+00	2.87E-02
CIDEB	NM_014430	3.57E-04	1.59E+00	7.78E-03
CIDECP1	NR_002786	7.82E-03	1.68E+00	4.88E-02
CILP	NM_003613	1.43E-03	1.74E+00	1.74E-02
CISD3	NM_001136498	7.12E-03	1.82E+00	4.61E-02
CKAP2	NM_018204	3.85E-04	-1.53E+00	8.09E-03
CKAP5	NM_014756	7.14E-03	-1.57E+00	4.62E-02
CKLF	NM_181640	3.79E-03	1.88E+00	3.11E-02
CLASP1	NM_015282	6.69E-04	-1.93E+00	1.10E-02
CLASP2	NM_001365634	4.12E-04	-1.60E+00	8.35E-03

CLCC1	NM_001278202	6.76E-04	2.01E+00	1.10E-02
CLCN1	NR_046453	7.96E-04	4.00E+00	1.22E-02
CLCN3	NM_001829	1.46E-05	-2.41E+00	1.27E-03
CLDN9	NM_020982	7.05E-03	2.85E+00	4.57E-02
CLEC10A	NM_001330070	5.47E-04	2.42E+00	9.82E-03
CLEC12A	NM_138337	2.62E-03	1.97E+00	2.52E-02
CLEC12B	NM_205852	2.10E-03	1.79E+00	2.20E-02
CLEC18B	NM_001011880	8.55E-04	2.72E+00	1.28E-02
CLIC1	NM_001287594	2.97E-03	1.70E+00	2.69E-02
CLIC4	NM_013943	1.92E-04	-1.85E+00	5.51E-03
CLINT1	NM_001195555	1.72E-06	-1.72E+00	4.95E-04
CLIP4	NR_109844	1.54E-03	-1.81E+00	1.82E-02
CLK2	NM_001363704	6.09E-05	1.65E+00	2.74E-03
CLN3	NM_001042432	3.96E-03	1.79E+00	3.19E-02
CLSPN	NM_022111	3.05E-03	-1.64E+00	2.73E-02
CLTB	NR_045724	4.74E-03	2.85E+00	3.57E-02
CLTCL1	NM_007098	4.11E-03	1.60E+00	3.26E-02
CMKLR1	NM_001142343	4.13E-07	-3.16E+00	3.14E-04
CMTM2	NM_001199317	2.04E-03	1.68E+00	2.16E-02
CMTM4	NM_178818	2.07E-03	1.76E+00	2.17E-02
CMTR2	NM_001324378	6.37E-03	-1.80E+00	4.29E-02
CNKSR2	NM_001330770	7.42E-03	-2.30E+00	4.74E-02
CNOT1	NR_049763	6.90E-07	-1.79E+00	3.69E-04
CNOT6	NM_001370472	3.15E-05	-1.73E+00	1.97E-03
CNOT6L	NM_001286790	2.54E-03	-1.52E+00	2.48E-02
CNOT7	NM_001322088	2.99E-03	-1.78E+00	2.70E-02
CNOT9	NR_073390	4.24E-03	1.58E+00	3.32E-02
CNPY2	NM_014255	1.05E-04	1.55E+00	3.89E-03
CNPY3	NM_001318847	1.57E-06	1.59E+00	4.73E-04
CNPY4	NM_152755	3.73E-07	1.59E+00	3.14E-04
CNTNAP2	NM_014141	1.19E-03	-3.18E+00	1.55E-02
CNTRL	NM_007018	6.56E-06	-2.69E+00	8.39E-04
CNTROB	NM_001353209	2.52E-03	3.78E+00	2.46E-02
COA1	NR_135580	1.42E-05	1.94E+00	1.25E-03
COA3	NM_001040431	1.12E-04	1.57E+00	4.03E-03
COA6	NM_001206641	1.88E-03	2.52E+00	2.07E-02
COASY	NM_001042529	1.58E-03	1.88E+00	1.86E-02
COBLL1	NM_001365671	3.55E-04	-4.13E+00	7.76E-03

COG4	NM_015386	3.29E-04	1.69E+00	7.47E-03
COMMD3	NM_012071	1.52E-03	1.62E+00	1.81E-02
COMMD5	NM_001081004	2.42E-03	1.57E+00	2.40E-02
COMMD9	NM_014186	2.98E-05	1.58E+00	1.92E-03
COPS6	NM_006833	1.03E-04	1.58E+00	3.83E-03
COPS7A	NM_001164093	4.34E-03	1.73E+00	3.37E-02
COPS9	NM_001163424	4.05E-03	2.12E+00	3.23E-02
COPZ2	NM_016429	5.20E-03	1.56E+00	3.79E-02
COQ8B	NM_001142555	4.47E-03	2.29E+00	3.44E-02
CORO7	NM_001201473	3.39E-03	3.25E+00	2.91E-02
COX14	NM_032901	3.69E-05	2.02E+00	2.13E-03
COX15	NM_004376	9.87E-06	2.32E+00	1.04E-03
COX17	NM_005694	7.59E-03	1.78E+00	4.80E-02
COX4I1	NM_001318794	5.81E-05	2.13E+00	2.67E-03
COX6A1	NM_004373	1.85E-03	1.60E+00	2.04E-02
COX6B1	NM_001863	4.64E-04	1.64E+00	8.94E-03
COX6C	NM_004374	3.66E-03	2.14E+00	3.05E-02
COX7A2	NR_158783	4.96E-04	2.03E+00	9.25E-03
COX7C	NM_001867	2.89E-03	2.41E+00	2.66E-02
CPAMD8	NR_147452	6.76E-05	2.50E+00	2.92E-03
CPEB2	NM_001177381	1.47E-03	-1.62E+00	1.77E-02
CPEB4	NM_030627	7.49E-04	-2.31E+00	1.18E-02
CPLANE1	NM_023073	4.67E-03	-1.73E+00	3.53E-02
CPNE1	NR_037188	3.89E-03	1.65E+00	3.16E-02
CPSF4	NM_006693	2.76E-03	1.97E+00	2.59E-02
CPT1B	NM_004377	2.35E-06	2.52E+00	5.56E-04
CRABP2	NM_001878	7.45E-04	1.60E+00	1.18E-02
CRAT	NM_001346549	3.80E-03	2.52E+00	3.12E-02
CRCP	NM_001142414	5.00E-03	1.80E+00	3.71E-02
CREB1	NM_134442	2.52E-03	-1.59E+00	2.46E-02
CREB3L2	NM_001318246	1.40E-05	-1.53E+00	1.24E-03
CREB3L4	NM_001255978	3.21E-03	1.90E+00	2.82E-02
CREBBP	NM_004380	8.44E-05	-2.03E+00	3.38E-03
CREBRF	NM_153607	1.68E-03	-1.70E+00	1.93E-02
CREBZF	NM_001039618	5.05E-03	-1.55E+00	3.73E-02
CRIM1	NM_016441	2.34E-05	-2.46E+00	1.66E-03
CRLF3	NM_015986	4.04E-05	-1.56E+00	2.21E-03
CRY1	NM_004075	9.34E-04	-1.59E+00	1.34E-02

CRYGS	NM_017541	9.28E-04	1.53E+00	1.34E-02
CRYZL2P	NR_151484	4.08E-03	2.59E+00	3.25E-02
CSDE1	NM_001242892	5.49E-05	2.38E+00	2.61E-03
CSF1R	NR_109969	5.94E-04	1.91E+00	1.03E-02
CSF2RA	NM_001161532	2.23E-03	2.00E+00	2.28E-02
CSGALNACT1	NM_001354476	8.05E-03	-2.38E+00	4.98E-02
CSNK1G3	NM_001364143	1.40E-03	-2.06E+00	1.72E-02
CSNK2A3	NM_001256686	1.50E-03	-1.50E+00	1.80E-02
CSRP1	NM_001193572	8.06E-03	1.61E+00	4.99E-02
CSTB	NM_000100	3.58E-05	1.83E+00	2.10E-03
CSTF2	NM_001306206	5.18E-03	-1.63E+00	3.78E-02
CTAGE1	NM_172241	6.86E-04	-1.88E+00	1.11E-02
CTCF	NM_001363916	6.69E-03	-1.52E+00	4.42E-02
CTDSP1	NM_001206878	2.33E-03	1.67E+00	2.34E-02
CTNNA1	NM_001324012	4.05E-04	5.50E+00	8.31E-03
CTNNBIP1	NM_001012329	1.29E-03	1.53E+00	1.64E-02
CTNS	NM_004937	4.62E-04	1.54E+00	8.94E-03
CTPS1	NM_001301237	2.22E-03	2.22E+00	2.27E-02
CTR9	NM_001346279	2.23E-03	-1.54E+00	2.28E-02
CTRL	NM_001907	4.24E-03	1.98E+00	3.32E-02
CTSF	NM_003793	2.02E-04	1.98E+00	5.68E-03
CTSG	NM_001911	2.22E-04	2.87E+00	5.95E-03
CTSH	NM_004390	2.21E-04	1.62E+00	5.95E-03
CTTN	NM_005231	1.05E-03	3.18E+00	1.44E-02
CTU1	NM_145232	5.50E-03	2.47E+00	3.94E-02
CUEDC1	NM_001271875	2.73E-03	2.18E+00	2.57E-02
CUEDC2	NM_024040	6.25E-03	1.78E+00	4.24E-02
CUL3	NM_001257197	1.72E-03	1.77E+00	1.96E-02
CUL4B	NM_001079872	2.62E-04	-1.78E+00	6.60E-03
CWC15	NM_001363372	4.20E-05	3.65E+00	2.23E-03
CWF19L1	NM_001303406	8.75E-05	2.66E+00	3.46E-03
CXorf65	NR_033212	5.39E-03	1.56E+00	3.89E-02
CXXC5	NM_001317204	6.74E-03	-2.05E+00	4.45E-02
CYB561D2	NM_001291284	5.88E-03	1.55E+00	4.08E-02
CYB5A	NM_148923	4.68E-04	1.55E+00	9.01E-03
CYB5D1	NM_001330110	1.30E-03	1.95E+00	1.64E-02
CYBC1	NR_036516	4.95E-04	2.13E+00	9.25E-03
CYHR1	NM_001129888	2.48E-03	2.63E+00	2.43E-02

CYP27A1	NM_000784	2.86E-03	1.74E+00	2.64E-02
CYP2U1	NM_183075	1.52E-03	-1.60E+00	1.81E-02
CYP4F12	NR_117085	4.03E-03	2.06E+00	3.23E-02
CYP7B1	NM_004820	7.06E-03	-1.88E+00	4.58E-02
CYTH2	NM_004228	4.50E-03	1.51E+00	3.45E-02
CYTH4	NM_001318024	8.96E-04	1.61E+00	1.31E-02
CYTOR	NR_024206	4.84E-04	2.22E+00	9.16E-03
CZIB	NM_017887	5.55E-04	1.75E+00	9.88E-03
DAAM1	NM_001270520	2.45E-04	-2.03E+00	6.36E-03
DALRD3	NM_001009996	5.61E-06	2.10E+00	8.21E-04
DANCR	NR_024031	3.83E-04	1.63E+00	8.07E-03
DBF4	NM_006716	1.58E-03	-2.11E+00	1.86E-02
DBH-AS1	NR_102735	1.40E-03	2.35E+00	1.73E-02
DCAF1	NM_001349170	6.40E-04	-1.65E+00	1.08E-02
DCAF16	NM_001345882	1.81E-03	-1.66E+00	2.02E-02
DCAF17	NM_025000	4.03E-03	-1.73E+00	3.23E-02
DCK	NM_000788	2.00E-04	-1.66E+00	5.66E-03
DCLRE1A	NM_014881	5.40E-04	-2.44E+00	9.78E-03
DCP1A	NM_018403	4.29E-06	-1.94E+00	7.14E-04
DCP2	NM_152624	1.82E-03	-1.81E+00	2.03E-02
DCTN2	NM_001348068	4.94E-03	1.86E+00	3.68E-02
DCTN3	NM_007234	3.14E-03	1.56E+00	2.78E-02
DDIT3	NM_004083	1.58E-03	1.85E+00	1.86E-02
DDX11	NM_152438	6.25E-03	3.27E+00	4.24E-02
DDX11L2	NR_024004	6.02E-06	7.62E+00	8.30E-04
DDX21	NM_004728	7.59E-06	-1.58E+00	8.87E-04
DDX39B	NM_080598	5.80E-06	2.02E+00	8.21E-04
DDX3X	NM_001363819	9.54E-05	-1.98E+00	3.67E-03
DDX41	NM_016222	7.49E-03	1.64E+00	4.76E-02
DDX51	NM_175066	7.66E-03	1.51E+00	4.82E-02
DDX56	NM_019082	1.78E-03	1.52E+00	2.01E-02
DDX6	NM_004397	9.27E-08	-1.59E+00	1.55E-04
DEF8	NM_001242821	4.94E-05	1.96E+00	2.46E-03
DEK	NM_003472	1.36E-04	-1.70E+00	4.48E-03
DENND10	NM_001303111	1.91E-03	-2.50E+00	2.08E-02
DENND1C	NM_001290331	1.50E-04	1.99E+00	4.72E-03
DENND4A	NM_001144823	1.51E-03	-1.68E+00	1.81E-02
DENND4B	NM_014856	2.10E-03	1.62E+00	2.20E-02

DENND4C	NM_001330640	6.35E-05	-2.79E+00	2.80E-03
DESI2	NM_016076	2.39E-04	-1.83E+00	6.26E-03
DGAT1	NM_012079	7.56E-03	2.14E+00	4.79E-02
DGAT2	NM_001253891	2.88E-03	1.97E+00	2.65E-02
DGKA	NR_147026	1.24E-03	2.79E+00	1.60E-02
DGKH	NM_152910	5.75E-05	-7.51E+00	2.66E-03
DHPS	NR_161469	5.46E-04	3.10E+00	9.81E-03
DHRS4	NM_001282989.1	4.57E-03	1.91E+00	3.49E-02
DHRS4L2	NM_001193637	7.54E-03	3.07E+00	4.78E-02
DHRS7	NM_016029	1.57E-03	1.52E+00	1.85E-02
DHRS9	NM_001142270	3.79E-04	-3.95E+00	8.04E-03
DHX29	NR_144324	1.46E-03	-1.77E+00	1.77E-02
DHX36	NM_020865	1.31E-03	-1.65E+00	1.65E-02
DHX40	NM_001166301	4.09E-04	1.53E+00	8.33E-03
DIAPH2	NM_007309	3.18E-04	-1.63E+00	7.31E-03
DICER1	NM_030621	3.10E-06	-2.18E+00	6.12E-04
DICER1-AS1	NR_015415	2.35E-03	1.67E+00	2.36E-02
DIP2A	NM_001353942	3.70E-05	-1.63E+00	2.13E-03
DIS3	NM_001322349	6.04E-04	-1.70E+00	1.04E-02
DLEU2	NR_152572	9.49E-04	-1.88E+00	1.36E-02
DLG1	NM_001366208	3.24E-04	-2.19E+00	7.40E-03
DLG4	NR_135527	1.05E-03	2.14E+00	1.44E-02
DLGAP1-AS1	NR_024101	1.95E-03	1.52E+00	2.11E-02
DLST	NR_033814	2.12E-03	2.15E+00	2.21E-02
DMAC1	NM_033428	3.01E-03	1.66E+00	2.71E-02
DMAC2	NM_001167869	7.79E-03	1.52E+00	4.87E-02
DMTF1	NR_024550	1.09E-03	-1.61E+00	1.47E-02
DMXL1	NM_001349240	3.47E-04	-2.04E+00	7.70E-03
DMXL2	NM_015263	5.59E-05	-3.36E+00	2.62E-03
DNAJA3	NM_001286516	6.48E-04	1.10E+01	1.08E-02
DNAJB14	NM_001031723	3.01E-04	-1.70E+00	7.10E-03
DNAJB6	NM_005494	1.88E-03	-1.58E+00	2.07E-02
DNAJC10	NM_018981	2.23E-04	-1.66E+00	5.98E-03
DNAJC13	NM_015268	1.38E-04	-1.83E+00	4.51E-03
DNAJC16	NR_109898	7.57E-03	-1.74E+00	4.79E-02
DNAJC17	NM_018163	1.01E-03	1.57E+00	1.41E-02
DNAJC27	NM_016544	3.40E-03	-1.65E+00	2.91E-02
DNM3	NM_001136127	2.65E-03	-2.06E+00	2.53E-02

DNMBP	NM_015221	2.79E-04	-2.02E+00	6.78E-03
DNPH1	NM_006443	4.10E-03	2.74E+00	3.26E-02
DOCK10	NM_001290263	1.57E-05	-2.17E+00	1.31E-03
DOCK11	NM_144658	3.18E-04	-1.53E+00	7.30E-03
DOCK5	NM_024940	7.83E-03	-1.52E+00	4.89E-02
DOCK7	NM_001272001	3.52E-03	-1.73E+00	2.98E-02
DOCK8	NM_203447	5.97E-04	-1.74E+00	1.03E-02
DOK1	NM_001197260	5.93E-03	1.80E+00	4.11E-02
DOP1A	NM_001199942	5.46E-03	-1.73E+00	3.92E-02
DPCD	NM_015448	1.95E-03	1.82E+00	2.11E-02
DPEP2	NM_001324159	4.46E-04	1.73E+00	8.73E-03
DPEP3	NM_001370198	2.49E-05	2.42E+00	1.73E-03
DPH7	NM_001346386	1.42E-03	2.18E+00	1.73E-02
DPP8	NR_135485	5.32E-05	-2.02E+00	2.58E-03
DPYD	NM_000110	1.85E-03	-1.65E+00	2.05E-02
DSE	NM_001322943	4.25E-03	3.76E+00	3.32E-02
DST	NM_015548	1.69E-04	-1.93E+00	5.10E-03
DTHD1	NM_001170700	2.18E-03	-3.50E+00	2.25E-02
DTX3L	NM_138287	3.38E-03	-1.58E+00	2.90E-02
DUS2	NM_001271762	2.34E-03	1.78E+00	2.35E-02
DUSP22	NM_020185	4.51E-04	1.82E+00	8.80E-03
DUSP23	NM_017823	2.49E-04	1.62E+00	6.39E-03
DYNLRB1	NM_014183	6.59E-03	1.50E+00	4.39E-02
DYRK4	NM_001282285	2.80E-04	1.82E+00	6.80E-03
E2F2	NM_004091	5.37E-03	1.58E+00	3.87E-02
E2F3	NM_001243076	5.21E-04	-2.03E+00	9.54E-03
E2F5	NM_001083589	2.79E-03	-1.55E+00	2.61E-02
EAF1	NM_033083	1.49E-07	-1.57E+00	1.72E-04
EBP	NM_006579	2.82E-03	1.71E+00	2.62E-02
EDC3	NM_001351379	1.73E-03	2.39E+00	1.97E-02
EDEM3	NM_025191	8.90E-04	-1.78E+00	1.31E-02
EDF1	NM_153200	8.65E-04	2.25E+00	1.28E-02
EDRF1	NR_110859	7.39E-04	-3.12E+00	1.17E-02
EEF1AKMT3	NM_015433	1.28E-03	1.72E+00	1.63E-02
EEF1B2	NM_001959	7.40E-03	2.21E+00	4.73E-02
EEF1D	NM_001317743	2.02E-04	2.14E+00	5.69E-03
EEF1G	NM_001404	2.70E-05	2.11E+00	1.80E-03
EFCAB8	NM_001143967	2.12E-03	1.66E+00	2.21E-02

EFNA4	NM_005227	1.92E-03	1.57E+00	2.09E-02
EFR3A	NM_015137	2.97E-05	-1.67E+00	1.92E-03
EFTUD2	NM_001142605	2.67E-03	2.11E+00	2.54E-02
EGF	NM_001178130	1.40E-03	-3.07E+00	1.73E-02
EGLN2	NM_053046	1.30E-03	2.00E+00	1.64E-02
EHBP1	NM_001142614	5.12E-03	-2.23E+00	3.76E-02
EIF2AK2	NM_002759	1.13E-06	-5.20E+00	4.12E-04
EIF2AK3	NM_001313915	9.20E-04	-1.58E+00	1.33E-02
EIF2B4	NM_015636	6.29E-03	1.86E+00	4.26E-02
EIF3A	NM_003750	5.70E-05	-1.59E+00	2.65E-03
EIF3G	NM_003755	4.20E-03	1.91E+00	3.30E-02
EIF3K	NM_013234	1.05E-03	1.71E+00	1.44E-02
EIF3L	NM_001363785	4.79E-04	2.21E+00	9.11E-03
EIF4E2	NM_004846	8.77E-04	1.52E+00	1.29E-02
EIF4G3	NM_003760	2.35E-04	-1.69E+00	6.18E-03
EIF5A	NM_001370420	5.59E-04	2.06E+00	9.91E-03
EIF6	NR_052022	6.27E-03	2.42E+00	4.24E-02
EIPR1	NM_003310	6.21E-03	1.53E+00	4.23E-02
ELF1	NM_172373	1.59E-06	-2.02E+00	4.73E-04
ELK4	NM_001973	5.60E-04	-1.65E+00	9.93E-03
ELMO1	NM_001206482	3.76E-03	-2.78E+00	3.09E-02
ELMOD3	NR_138133	6.04E-07	2.06E+00	3.62E-04
ELMSAN1	NM_001367710	2.52E-03	-1.62E+00	2.46E-02
ELOA-AS1	NR_038280	5.12E-04	1.57E+00	9.44E-03
ELOVL1	NM_001256399	6.88E-03	1.52E+00	4.50E-02
ELOVL5	NM_021814	1.08E-03	-1.52E+00	1.46E-02
ELOVL6	NM_024090	4.34E-03	-2.37E+00	3.36E-02
ELP1	NM_003640	1.39E-03	-1.69E+00	1.72E-02
ELP6	NM_001031703	1.12E-04	1.56E+00	4.02E-03
EMC6	NM_031298	1.22E-05	2.17E+00	1.15E-03
EMG1	NR_135131	3.29E-04	2.04E+00	7.47E-03
EML4	NM_019063	1.48E-04	-1.74E+00	4.69E-03
EMP3	NM_001425	4.97E-03	1.56E+00	3.70E-02
EMSY	NM_020193	2.74E-05	-1.73E+00	1.82E-03
ENC1	NM_001256574	3.85E-03	-2.63E+00	3.14E-02
ENKD1	NR_138150	2.09E-03	3.11E+00	2.19E-02
ENO2	NM_001975	2.54E-03	1.73E+00	2.48E-02
ENO3	NM_053013	3.43E-03	2.04E+00	2.93E-02

ENPP4	NM_014936	1.12E-06	-2.82E+00	4.12E-04
ENPP5	NM_001290072	3.87E-03	-2.85E+00	3.15E-02
ENTPD6	NM_001322381	1.11E-04	9.46E+00	4.00E-03
ENTR1	NM_001039708	1.85E-03	1.52E+00	2.05E-02
ENY2	NM_020189	4.29E-03	1.53E+00	3.34E-02
EOMES	NM_001278183	9.96E-04	-1.99E+00	1.40E-02
EP300	NM_001429	4.75E-05	-1.88E+00	2.40E-03
EPAS1	NM_001430	4.41E-03	-1.63E+00	3.40E-02
EPB41	NM_001166007	1.09E-04	-1.64E+00	3.98E-03
EPB41L2	NM_001350304	3.46E-03	-2.48E+00	2.95E-02
EPB41L4A	NM_022140	7.05E-04	-2.39E+00	1.14E-02
EPC2	NM_015630	1.83E-06	-1.65E+00	4.95E-04
EPG5	NM_020964	5.09E-08	-1.64E+00	1.37E-04
EPHA1	NM_005232	7.81E-03	2.01E+00	4.88E-02
EPHA1-AS1	NR_033897	5.82E-03	-2.62E+00	4.06E-02
EPHB6	NR_104001	1.67E-03	4.24E+00	1.92E-02
EPS15	NM_001981	5.91E-05	-1.99E+00	2.69E-03
EPS8	NM_004447	3.53E-03	-1.83E+00	2.98E-02
ERAL1	NM_001317985	2.12E-03	2.25E+00	2.21E-02
ERAP1	NM_001349244	2.59E-03	-1.91E+00	2.50E-02
ERAP2	NM_022350	4.35E-04	-4.99E+00	8.63E-03
ERBIN	NM_001006600	1.03E-05	-1.86E+00	1.05E-03
ERC1	NM_178039	3.25E-03	-2.20E+00	2.84E-02
ERCC1	NM_001369414	3.35E-03	1.70E+00	2.89E-02
ERCC4	NM_005236	3.01E-05	-1.56E+00	1.92E-03
ERCC6	NM_001346440	1.86E-04	-1.65E+00	5.38E-03
ERFL	NM_001365103	1.31E-03	2.80E+00	1.65E-02
ERGIC3	NM_015966	2.34E-04	1.75E+00	6.18E-03
ERMP1	NM_024896	2.17E-04	-1.56E+00	5.91E-03
ERP27	NM_152321	1.28E-03	1.51E+00	1.63E-02
ERRFI1	NM_018948	5.01E-03	-1.74E+00	3.71E-02
ERV3-1-ZNF117	NM_001348050	5.55E-03	-2.86E+00	3.95E-02
ESF1	NM_001276380	3.64E-04	-1.94E+00	7.86E-03
ETFB	NM_001014763	6.54E-03	1.61E+00	4.37E-02
EVI5	NM_005665	1.89E-03	-1.55E+00	2.07E-02
EWSR1	NM_001163286	3.93E-03	3.38E+00	3.18E-02
EXOC7	NM_001145298	3.66E-03	2.14E+00	3.05E-02
EXOC8	NM_175876	8.18E-05	-1.71E+00	3.30E-03

EXOSC10	NM_002685	1.36E-03	-2.14E+00	1.69E-02
EZH1	NM_001321082	4.06E-04	1.78E+00	8.31E-03
F12	NM_000505	2.69E-03	2.78E+00	2.55E-02
F2R	NM_001992	2.74E-07	-2.77E+00	2.69E-04
F2RL1	NM_005242	6.54E-07	-2.31E+00	3.69E-04
FAAP20	NM_001256946	6.05E-03	1.96E+00	4.15E-02
FAHD2A	NM_016044	5.91E-04	1.51E+00	1.02E-02
FAHD2B	NM_199336	6.92E-03	2.34E+00	4.52E-02
FAHD2CP	NR_003698	2.92E-03	1.70E+00	2.67E-02
FAM107B	NM_001282700	1.64E-05	-1.92E+00	1.34E-03
FAM110A	NM_207121	2.69E-04	2.63E+00	6.66E-03
FAM111A	NM_001312910	1.78E-03	-2.10E+00	2.00E-02
FAM120B	NM_001286381	5.03E-03	2.03E+01	3.72E-02
FAM126B	NM_173822	2.23E-03	-2.00E+00	2.28E-02
FAM135A	NM_001330998	3.39E-07	-5.99E+00	2.98E-04
FAM13B	NM_016603	1.17E-04	-1.71E+00	4.10E-03
FAM156A	NM_001242490	4.11E-03	5.26E+00	3.26E-02
FAM160B1	NM_020940	2.21E-03	-1.54E+00	2.27E-02
FAM160B2	NR_148727	2.53E-03	2.78E+00	2.46E-02
FAM172A	NM_032042	2.82E-06	-2.12E+00	5.95E-04
FAM174B	NM_207446	7.81E-04	1.89E+00	1.20E-02
FAM177B	NR_136691	2.72E-03	-3.04E+00	2.57E-02
FAM189B	NM_006589	5.27E-03	1.62E+00	3.83E-02
FAM199X	NM_207318	1.61E-04	-1.53E+00	4.92E-03
FAM214A	NR_104457	4.03E-04	-1.63E+00	8.31E-03
FAM217B	NM_022106	1.09E-03	-1.74E+00	1.48E-02
FAM219B	NR_135846	3.39E-05	2.29E+00	2.06E-03
FAM239B	NR_146578	1.21E-05	5.61E+00	1.15E-03
FAM3A	NM_001282312	2.23E-03	3.17E+00	2.28E-02
FAM50A	NM_004699	2.32E-03	1.84E+00	2.33E-02
FAM72A	NM_001123168	1.52E-04	-2.95E+00	4.76E-03
FAM83A	NM_032899	1.53E-03	5.23E+00	1.82E-02
FAM86FP	NR_024254	7.18E-04	2.06E+00	1.15E-02
FAM91A1	NM_001317918	3.32E-05	-3.16E+00	2.02E-03
FAM95C	NR_047651	1.51E-03	2.17E+00	1.81E-02
FAM98C	NM_174905	4.76E-03	2.24E+00	3.58E-02
FANCM	NM_020937	2.17E-03	-2.26E+00	2.24E-02
FAR2P2	NR_046260	4.83E-06	-4.00E+00	7.54E-04

FAU	NM_001997	3.00E-03	2.70E+00	2.71E-02
FBL	NM_001436	7.33E-04	1.92E+00	1.16E-02
FBXL3	NM_012158	2.99E-05	-1.81E+00	1.92E-03
FBXL4	NM_001278716	1.84E-04	-1.60E+00	5.36E-03
FBXO11	NM_025133	7.32E-03	-1.53E+00	4.69E-02
FBXO28	NM_015176	4.60E-04	-1.74E+00	8.92E-03
FBXO44	NM_001330355	7.00E-03	5.41E+00	4.55E-02
FBXW11	NM_033644	9.87E-08	-2.02E+00	1.57E-04
FCGR1B	NM_001017986	7.62E-03	1.88E+00	4.80E-02
FCGRT	NM_004107	1.28E-03	2.05E+00	1.63E-02
FCHO2	NM_138782	2.28E-03	-1.75E+00	2.31E-02
FCHSD1	NM_033449	2.64E-03	1.73E+00	2.53E-02
FCN1	NM_002003	7.19E-04	1.68E+00	1.15E-02
FCRL1	NM_052938	5.83E-04	-1.96E+00	1.02E-02
FCRL6	NM_001284217	1.12E-03	-2.21E+00	1.50E-02
FCSK	NM_145059	7.67E-03	1.78E+00	4.82E-02
FEM1B	NM_015322	3.72E-05	-1.56E+00	2.13E-03
FER	NR_146155	3.74E-03	-1.93E+00	3.08E-02
FES	NM_001143783	1.95E-03	2.13E+00	2.11E-02
FGD5-AS1	NR_046254	5.28E-03	-1.53E+00	3.83E-02
FHIT	NM_001320900	1.10E-04	2.40E+00	4.00E-03
FHL3	NM_001243878	1.26E-03	2.13E+00	1.61E-02
FHOD1	NM_001318202	1.60E-03	2.32E+00	1.87E-02
FIBP	NM_004214	7.72E-04	1.59E+00	1.20E-02
FIGNL1	NM_001287492	1.44E-03	-3.31E+00	1.75E-02
FIS1	NM_016068	7.00E-03	2.53E+00	4.55E-02
FKBP11	NM_016594	5.33E-03	1.66E+00	3.86E-02
FLAD1	NM_001184892	4.09E-03	1.59E+00	3.25E-02
FLJ37453	NR_024279	2.16E-03	1.65E+00	2.24E-02
FLVCR1	NM_014053	3.78E-03	-1.50E+00	3.11E-02
FLYWCH1	NM_032296	7.36E-03	2.33E+00	4.71E-02
FMR1	NM_001185082	4.40E-04	-2.01E+00	8.67E-03
FNBP1L	NM_001024948	4.00E-04	-1.93E+00	8.27E-03
FNDC3A	NM_001079673	4.13E-06	-2.30E+00	7.03E-04
FNDC3B	NM_001135095	7.07E-04	-1.61E+00	1.14E-02
FNIP1	NM_001008738	1.34E-04	-2.12E+00	4.44E-03
FNIP2	NM_020840	2.65E-04	-2.03E+00	6.63E-03
FOPNL	NM_144600	1.80E-04	-1.66E+00	5.28E-03

FOXJ3	NM_014947	9.20E-04	-1.55E+00	1.33E-02
FOXN2	NM_002158	3.33E-03	-1.57E+00	2.88E-02
FOXN3	NM_005197	1.85E-07	-1.52E+00	2.06E-04
FOXO3	NM_201559	1.04E-03	-1.56E+00	1.43E-02
FOXO3B	NM_001368135	4.02E-06	-2.11E+00	7.03E-04
FOXP1	NM_001244816	2.53E-03	-1.75E+00	2.47E-02
FOXQ1	NM_033260	2.55E-03	1.03E+01	2.48E-02
FOXRED2	NM_001363042	6.55E-03	1.54E+00	4.38E-02
FPGS	NR_110170	7.50E-04	4.43E+00	1.18E-02
FRMD3	NM_001244960	6.93E-03	-2.03E+00	4.52E-02
FRS2	NM_001278356	1.57E-06	-1.97E+00	4.73E-04
FRYL	NM_015030	5.98E-09	-1.76E+00	3.33E-05
FSTL3	NM_005860	9.77E-05	2.97E+00	3.73E-03
FTL	NM_000146	1.66E-03	1.96E+00	1.92E-02
FTO	NM_001363899	3.53E-03	-1.90E+00	2.98E-02
FUT11	NM_173540	7.78E-06	-2.44E+00	9.06E-04
FXYD5	NM_014164	7.90E-03	1.61E+00	4.91E-02
FYB1	NM_001349333	6.35E-05	-2.34E+00	2.80E-03
FYTTD1	NM_001011537	5.15E-04	-1.51E+00	9.49E-03
G3BP2	NM_203504	8.83E-07	-1.60E+00	3.78E-04
GAA	NR_134848	3.46E-03	1.94E+00	2.95E-02
GABPB2	NM_001323910	1.01E-04	-2.04E+00	3.80E-03
GAL3ST4	NM_024637	1.92E-03	2.34E+00	2.09E-02
GALNT11	NM_001304514	6.98E-05	6.22E+00	2.98E-03
GALNT4	NM_003774	4.30E-03	-2.24E+00	3.35E-02
GALNT7	NM_017423	7.25E-04	-1.57E+00	1.16E-02
GANAB	NM_001278193	2.13E-03	1.56E+00	2.21E-02
GAPDH	NM_001289746	2.22E-04	2.51E+00	5.95E-03
GAPVD1	NM_001282679	4.16E-06	-2.69E+00	7.03E-04
GAS5	NR_152522	5.00E-04	2.61E+00	9.29E-03
GAS6	NM_000820	5.43E-04	2.52E+00	9.79E-03
GATB	NM_001363341	1.74E-03	2.64E+00	1.98E-02
GATD1	NM_001318820	1.22E-04	2.00E+00	4.20E-03
GATD3A	NM_004649.1	6.65E-03	1.84E+00	4.41E-02
GATD3B	NM_001363758.1	6.65E-03	1.84E+00	4.41E-02
GBA	NM_001171811	8.76E-04	5.56E+00	1.29E-02
GBAP1	NR_002188	2.48E-04	2.21E+00	6.39E-03
GBP3	NR_135007	5.51E-03	-2.44E+00	3.94E-02

GBP5	NM_052942	6.44E-03	-2.97E+00	4.32E-02
GCC2	NM_181453	7.00E-04	-1.80E+00	1.13E-02
GCH1	NM_000161	5.80E-03	-1.66E+00	4.05E-02
GCHFR	NM_005258	5.99E-03	2.84E+00	4.13E-02
GDE1	NM_001324067	1.10E-03	2.28E+00	1.48E-02
GDPD3	NM_024307	1.21E-05	2.12E+00	1.15E-03
GEMIN8	NM_001042480	2.98E-03	1.98E+00	2.70E-02
GEN1	NM_001130009	6.14E-03	-1.63E+00	4.19E-02
GFI1	NM_001127216	1.04E-04	-1.90E+00	3.86E-03
GFOD1	NM_018988	7.06E-05	-1.72E+00	3.00E-03
GGA1	NM_001001560	2.87E-03	2.59E+00	2.64E-02
GGA3	NM_001291642	6.24E-05	2.27E+00	2.78E-03
GGCX	NM_001311312	6.63E-05	1.69E+00	2.88E-03
GGT1	NM_013421	6.48E-03	2.01E+00	4.34E-02
GHRL	NM_001302822	2.32E-04	4.45E+00	6.15E-03
GIGYF2	NM_001103146	2.28E-04	-1.53E+00	6.08E-03
GIMAP5	NM_018384	4.98E-04	1.65E+00	9.28E-03
GK5	NR_033289	9.50E-04	-1.94E+00	1.36E-02
GLCCI1	NM_138426	6.95E-06	-1.92E+00	8.64E-04
GLCE	NM_001324093	8.59E-05	-3.05E+00	3.42E-03
GLG1	NR_027265	3.09E-04	6.67E+00	7.19E-03
GLIPR2	NM_001287013	1.33E-03	1.63E+00	1.66E-02
GLMP	NM_001256605	4.37E-03	2.18E+00	3.38E-02
GLYCTK	NR_026699	1.06E-03	2.81E+00	1.45E-02
GLYR1	NR_136697	3.21E-03	-1.79E+00	2.82E-02
GMCL2	NM_001358008	4.09E-05	-2.59E+00	2.21E-03
GMFG	NM_004877	3.71E-03	1.63E+00	3.07E-02
GMPPA	NM_013335	2.96E-03	1.89E+00	2.69E-02
GMPR2	NM_001283022	4.88E-04	1.62E+00	9.19E-03
GNA13	NM_006572	8.22E-05	-2.00E+00	3.31E-03
GNAI2	NM_001282618	3.03E-03	1.82E+00	2.73E-02
GNAL	NM_001369387	5.82E-04	-5.84E+00	1.01E-02
GNAS	NR_132272	4.74E-04	-1.56E+00	9.07E-03
GNE	NM_005476	1.42E-04	-2.02E+00	4.55E-03
GNG2	NM_001243774	1.10E-03	-2.33E+00	1.48E-02
GNL2	NM_001323624	3.29E-03	4.82E+00	2.86E-02
GNL3L	NM_019067	1.36E-05	-1.51E+00	1.22E-03
GNPTAB	NM_024312	3.18E-05	-1.66E+00	1.97E-03

GOLGA2P7	NR_027001	5.07E-03	2.27E+00	3.74E-02
GOLGA4	NM_001172713	1.93E-05	-1.79E+00	1.48E-03
GOLGA8N	NM_001282494.1	4.25E-05	-1.86E+00	2.25E-03
GOLGA8O	NM_001277308	1.42E-07	-2.85E+00	1.70E-04
GOLGA8R	NM_001282484	1.36E-04	-2.32E+00	4.48E-03
GOLIM4	NM_001308155	1.46E-04	-1.78E+00	4.66E-03
GON4L	NM_032292	2.92E-04	-2.44E+00	6.94E-03
GOPC	NM_001017408	4.50E-03	-1.52E+00	3.46E-02
GORASP1	NM_001278790	7.48E-03	1.66E+00	4.76E-02
GOSR2	NR_148349	2.40E-03	1.66E+00	2.39E-02
GPALPP1	NM_018559	4.19E-03	-1.56E+00	3.30E-02
GPAM	NM_001244949	1.94E-03	-1.63E+00	2.10E-02
GPANK1	NM_001199240	8.05E-04	1.56E+00	1.22E-02
GPATCH11	NM_174931	7.30E-04	-1.83E+00	1.16E-02
GPATCH2L	NR_110314	6.63E-04	-2.71E+00	1.09E-02
GPATCH8	NM_001002909	3.66E-05	-1.92E+00	2.13E-03
GPBP1	NM_022913	6.15E-06	-2.01E+00	8.32E-04
GPR132	NM_001278696	5.68E-03	2.78E+00	4.01E-02
GPR135	NM_022571	6.27E-04	-1.99E+00	1.06E-02
GPR155	NM_001033045	5.72E-04	-1.51E+00	1.00E-02
GPR162	NM_014449	4.19E-03	2.03E+00	3.30E-02
GPX1	NM_201397	5.57E-03	2.90E+00	3.96E-02
GPX3	NM_002084	3.98E-04	1.67E+00	8.25E-03
GPX4	NM_001039847	2.50E-04	3.02E+00	6.40E-03
GRAMD1A	NM_001320035	2.67E-03	2.24E+00	2.54E-02
GRHL1	NM_198182	3.09E-03	-2.27E+00	2.75E-02
GSAP	NR_146937	3.82E-03	-1.78E+00	3.13E-02
GSEC	NR_033839	1.51E-03	2.35E+00	1.80E-02
GSK3B	NM_001354596	7.51E-08	-3.07E+00	1.42E-04
GSN	NM_001258029	6.32E-03	1.84E+00	4.27E-02
GSTK1	NM_001143679	2.05E-03	2.11E+00	2.16E-02
GTF2A1	NM_015859	1.85E-05	-2.17E+00	1.44E-03
GTF2H2	NM_001364567	7.96E-04	-2.30E+00	1.22E-02
GTF2H2B	NR_033417	2.22E-05	-1.84E+00	1.61E-03
GTF2I	NM_033000	4.05E-03	-1.60E+00	3.23E-02
GTF2IRD2	NM_173537	3.13E-04	1.54E+00	7.24E-03
GTF3C4	NM_012204	4.18E-05	-1.52E+00	2.22E-03
GTPBP8	NM_014170	1.37E-03	-1.61E+00	1.70E-02

GUCY1A1	NM_000856	6.60E-03	-2.11E+00	4.39E-02
GUCY1B1	NM_000857	4.04E-03	-2.86E+00	3.23E-02
GUF1	NM_001345867	4.94E-03	-1.52E+00	3.68E-02
GUSB	NM_001293105	3.34E-04	1.92E+00	7.52E-03
GUSBP11	NR_024448	7.03E-06	1.66E+00	8.64E-04
GVINP1	NR_003945	3.25E-06	-2.08E+00	6.32E-04
GYS1	NR_027763	7.61E-04	1.74E+00	1.19E-02
H2AFV	NM_201517	6.16E-03	-3.01E+00	4.20E-02
H2AFY2	NM_018649	1.42E-04	1.75E+00	4.56E-03
HACD2	NM_198402	4.55E-06	-1.90E+00	7.25E-04
HAR1A	NR_003244	3.55E-04	3.30E+00	7.76E-03
HAUS6	NM_017645	5.71E-04	-1.79E+00	1.00E-02
HAX1	NM_006118	2.77E-06	1.88E+00	5.89E-04
HCFC1R1	NM_001288668	7.60E-03	2.26E+00	4.80E-02
HDAC3	NR_149168	2.79E-05	1.75E+00	1.83E-03
HDAC9	NM_178425	2.62E-03	-1.56E+00	2.52E-02
HDDC3	NM_001286451	3.64E-05	1.82E+00	2.13E-03
HEATR5A	NM_015473	3.21E-04	-1.65E+00	7.34E-03
HEATR5B	NM_019024	4.10E-06	-1.62E+00	7.03E-04
HECA	NM_016217	3.94E-05	-1.74E+00	2.20E-03
HECTD1	NM_015382	1.10E-05	-1.56E+00	1.09E-03
HEG1	NM_020733	1.13E-03	-1.88E+00	1.51E-02
HEIH	NR_045680	1.22E-03	1.50E+00	1.58E-02
HELZ	NM_014877	4.55E-07	-1.59E+00	3.14E-04
HERC3	NM_014606	7.01E-03	-1.54E+00	4.56E-02
HERC4	NM_015601	2.95E-04	-1.67E+00	6.98E-03
HEXA	NM_000520	5.71E-04	1.69E+00	1.00E-02
HGSNAT	NM_001363228	1.80E-04	1.79E+00	5.28E-03
HHLA3	NM_001031693	5.68E-03	2.59E+00	4.01E-02
HID1	NM_030630	2.94E-03	2.69E+00	2.68E-02
HIF1A	NM_001530	1.59E-05	-1.69E+00	1.31E-03
HINFP	NM_001351962	3.17E-04	1.87E+00	7.30E-03
HINT1	NM_005340	9.12E-04	2.12E+00	1.32E-02
HINT2	NM_032593	1.35E-05	1.80E+00	1.22E-03
HIPK1	NM_198268	5.37E-06	-3.34E+00	7.98E-04
HIPK2	NM_001113239	4.33E-07	-2.27E+00	3.14E-04
HIPK3	NM_005734	3.96E-05	-2.60E+00	2.20E-03
HIST1H1C	NM_005319	2.97E-03	1.97E+00	2.69E-02

HIVEP2	NM_006734	7.52E-05	-1.80E+00	3.13E-03
HLCS	NR_148020	6.01E-03	-2.04E+00	4.14E-02
HMBOX1	NR_136758	5.82E-03	-1.78E+00	4.06E-02
HMG20A	NM_018200	2.72E-04	-2.32E+00	6.69E-03
HMGB1	NM_002128	2.64E-03	5.38E+00	2.53E-02
HMGCR	NM_000859	4.77E-03	-1.54E+00	3.59E-02
HMGCS1	NM_001330663	2.90E-03	-1.62E+00	2.66E-02
HMGXB3	NM_001366501	2.78E-04	1.89E+00	6.77E-03
HMOX2	NM_002134	2.99E-03	1.62E+00	2.70E-02
HNMT	NM_001024074	1.06E-03	2.10E+00	1.45E-02
HNRNPA3	NM_001330248	1.70E-05	2.55E+00	1.38E-03
HNRNPA3P1	NR_002726	1.38E-03	-1.51E+00	1.71E-02
HNRNPH1	NM_001364232	8.00E-03	-1.57E+00	4.96E-02
HNRNPH3	NM_001322446	7.81E-03	-1.50E+00	4.88E-02
HNRNPK	NM_001318187	7.95E-03	-1.58E+00	4.94E-02
HNRNPLL	NM_001142650	1.24E-03	-1.51E+00	1.60E-02
HNRNPR	NM_005826	1.21E-03	-1.58E+00	1.57E-02
HOMER3	NR_027297	1.50E-03	2.12E+00	1.80E-02
HOOK2	NM_001100176	5.81E-03	2.17E+00	4.05E-02
HOXC4	NM_153633	1.17E-04	-2.45E+00	4.10E-03
HP	NM_001318138	4.17E-03	6.98E+00	3.29E-02
HPS1	NM_001322480	1.15E-03	2.31E+00	1.52E-02
HPS3	NM_032383	4.64E-04	-1.58E+00	8.94E-03
HPS4	NR_146313	2.63E-03	1.68E+00	2.52E-02
HPS5	NM_007216	1.59E-04	-1.70E+00	4.90E-03
HPSE	NM_001199830	1.00E-03	3.72E+00	1.40E-02
HRK	NM_003806	7.61E-04	-6.26E+00	1.19E-02
HS3ST3B1	NM_006041	2.05E-04	-1.93E+00	5.71E-03
HSBP1L1	NM_001136180	5.58E-04	2.08E+00	9.91E-03
HSCB	NM_172002	6.64E-03	1.54E+00	4.41E-02
HSD17B10	NM_004493	3.06E-03	1.69E+00	2.73E-02
HSD17B12	NM_016142	9.58E-04	-1.51E+00	1.36E-02
HSD17B8	NM_014234	2.03E-04	2.04E+00	5.69E-03
HSF4	NM_001538	1.66E-03	3.54E+00	1.92E-02
HSPA13	NM_006948	6.73E-04	-1.55E+00	1.10E-02
HTRA2	NM_001321727	3.13E-03	1.55E+00	2.77E-02
HYPK	NM_016400	1.48E-04	1.52E+00	4.69E-03
IAH1	NM_001039613	3.06E-03	1.51E+00	2.74E-02

IBTK	NM_015525	7.76E-03	-1.59E+00	4.86E-02
ICA1L	NM_138468	4.55E-03	-1.73E+00	3.48E-02
ICE1	NM_015325	1.04E-04	-1.57E+00	3.85E-03
ICE2	NM_024611	6.88E-03	-1.54E+00	4.50E-02
ID2-AS1	NR_110153	2.36E-03	1.99E+00	2.36E-02
IDE	NM_001322795	7.33E-05	-2.47E+00	3.07E-03
IDH3B	NM_001258384	7.48E-03	1.69E+00	4.76E-02
IDNK	NM_001001551	2.04E-04	2.64E+00	5.71E-03
IDUA	NM_001363576	6.51E-03	2.18E+00	4.36E-02
IFFO1	NM_001193457	4.00E-04	2.07E+00	8.27E-03
IFI27L2	NM_032036	5.19E-04	2.33E+00	9.51E-03
IFNAR2	NM_001289125	2.72E-04	-1.86E+00	6.69E-03
IFT43	NM_001102564	3.89E-05	1.87E+00	2.19E-03
IGFLR1	NR_144339	3.09E-03	1.88E+00	2.75E-02
IKBKE	NM_001193321	2.76E-03	1.60E+00	2.59E-02
IKZF1	NM_001220767	1.40E-05	-2.01E+00	1.24E-03
IKZF2	NM_001371275	1.97E-04	-2.49E+00	5.61E-03
IKZF3	NM_001284514	8.84E-05	-2.47E+00	3.48E-03
IL12RB2	NM_001258214	7.60E-03	-3.61E+00	4.80E-02
IL17RC	NR_037807	9.63E-04	2.71E+00	1.36E-02
IL1RAP	NM_002182	4.66E-05	-2.46E+00	2.37E-03
IL1RL1	NM_016232	1.75E-03	-5.87E+00	1.98E-02
IL23A	NM_016584	3.18E-03	2.01E+00	2.80E-02
IL32	NM_001308078	5.60E-03	2.02E+00	3.97E-02
IL4R	NM_001257997	6.54E-03	1.81E+00	4.37E-02
IL6ST	NM_002184	7.52E-05	-1.98E+00	3.13E-03
IL7R	NM_002185	4.60E-03	-1.85E+00	3.50E-02
IL9R	NM_002186	1.66E-03	2.48E+00	1.92E-02
INF2	NM_032714	1.39E-03	1.87E+00	1.72E-02
ING4	NM_016162	2.30E-05	1.63E+00	1.64E-03
INO80B-WBP1	NR_037849	8.51E-05	1.57E+00	3.40E-03
INO80D	NM_017759	7.84E-06	-1.56E+00	9.10E-04
INPP5F	NM_014937	6.24E-03	-1.79E+00	4.24E-02
INSC	NM_001278316	4.71E-04	2.10E+00	9.04E-03
INSIG2	NM_001321329	1.01E-04	-1.74E+00	3.80E-03
INTS2	NM_020748	7.09E-03	-1.50E+00	4.59E-02
INTS3	NM_001324475	3.08E-05	2.13E+00	1.96E-03
IPMK	NM_152230	7.82E-03	-1.64E+00	4.88E-02

IPO8	NM_006390	1.32E-05	-2.58E+00	1.20E-03
IPP	NM_005897	3.74E-03	-1.61E+00	3.08E-02
IQCC	NM_018134	1.14E-03	1.60E+00	1.51E-02
IQGAP1	NM_003870	1.38E-03	-1.51E+00	1.71E-02
IQGAP2	NM_001285460	3.53E-05	-2.97E+00	2.10E-03
IQSEC1	NM_014869	8.94E-04	2.37E+00	1.31E-02
IQSEC2	NM_001243197	7.33E-03	1.91E+00	4.70E-02
IRAK4	NM_001351344	1.21E-04	1.86E+00	4.18E-03
IREB2	NM_004136	2.46E-06	-2.02E+00	5.67E-04
IRF4	NM_002460	1.48E-04	-1.63E+00	4.69E-03
IRF6	NM_006147	2.27E-03	-3.06E+00	2.31E-02
IRS1	NM_005544	2.14E-03	-2.34E+00	2.22E-02
IST1	NM_001270979	4.18E-03	1.52E+00	3.30E-02
ISYNA1	NR_045573	3.69E-04	2.97E+00	7.93E-03
ITCH	NM_001324198	4.47E-06	-1.72E+00	7.15E-04
ITGA10	NM_001303041	7.12E-03	1.62E+00	4.61E-02
ITGA7	NM_001144997	5.49E-05	5.57E+00	2.61E-03
ITGAL	NM_001114380	2.24E-03	1.77E+00	2.28E-02
ITGAX	NM_000887	5.25E-04	1.99E+00	9.60E-03
ITGB1	NM_002211	7.02E-07	-1.70E+00	3.69E-04
ITGB2-AS1	NR_038312	2.88E-10	2.95E+00	3.21E-06
ITPA	NM_033453	2.00E-03	1.78E+00	2.13E-02
ITPR1	NM_001099952	6.48E-06	-2.04E+00	8.39E-04
ITPRID2	NR_109843	6.83E-03	-1.81E+00	4.48E-02
ITSN2	NM_001348184	2.86E-04	-1.99E+00	6.83E-03
IZUMO4	NM_001363588	1.75E-03	2.69E+00	1.98E-02
JADE1	NM_001287439	1.77E-03	-1.59E+00	2.00E-02
JADE3	NM_001077445	2.03E-04	-1.74E+00	5.69E-03
JAK2	NM_001322196	2.70E-04	-2.38E+00	6.68E-03
JAKMIP2	NM_014790	3.54E-04	-2.27E+00	7.75E-03
JAML	NM_153206	3.96E-05	2.45E+00	2.20E-03
JARID2	NM_004973	3.31E-04	-1.68E+00	7.49E-03
JMJD1C	NM_001282948	7.18E-08	-2.83E+00	1.42E-04
JMY	NM_152405	8.06E-03	-1.53E+00	4.99E-02
JRK	NM_001077527	9.05E-04	2.01E+00	1.32E-02
JRKL	NM_001261833	2.66E-03	-1.90E+00	2.54E-02
KANK1	NM_001256877	6.02E-04	2.58E+00	1.04E-02
KAT2A	NM_021078	5.58E-03	1.74E+00	3.97E-02

KAT6A	NM_006766	1.15E-05	-1.72E+00	1.11E-03
KAT6B	NM_001370139	9.37E-06	-2.81E+00	1.01E-03
KATNAL1	NM_001014380	3.16E-03	-1.55E+00	2.79E-02
KATNBL1P6	NR_003954	4.04E-03	-1.76E+00	3.23E-02
KAZN	NM_201628	2.15E-05	3.42E+00	1.59E-03
KBTBD8	NM_032505	2.90E-04	-1.74E+00	6.90E-03
KCNA3	NR_109846	4.12E-03	-1.80E+00	3.27E-02
KCNAB2	NM_001199860	3.04E-04	2.56E+00	7.14E-03
KCNAB3	NM_004732	3.27E-04	1.60E+00	7.45E-03
KCNMA1	NM_002247	3.65E-03	-1.32E+01	3.05E-02
KCNMB4	NM_014505	2.29E-03	1.53E+00	2.32E-02
KCNQ1	NM_181798	6.65E-03	1.74E+00	4.41E-02
KCNS1	NM_001322799	2.88E-03	1.92E+00	2.65E-02
KCTD17	NM_001282686	4.07E-03	2.56E+00	3.24E-02
KCTD20	NM_173562	5.72E-05	-1.71E+00	2.66E-03
KCTD7	NM_001167961	1.07E-03	2.08E+00	1.46E-02
KDM1B	NM_001364614	2.04E-04	-1.85E+00	5.70E-03
KDM4C	NM_001354001	1.37E-03	-2.49E+00	1.70E-02
KDM5C	NR_148672	3.45E-03	-2.85E+00	2.94E-02
KDM6A	NR_111960	5.54E-05	-2.39E+00	2.61E-03
KDM7A	NM_030647	7.67E-04	-1.56E+00	1.19E-02
KHDRBS1	NM_006559	6.15E-05	-1.51E+00	2.76E-03
KIAA0100	NM_001363827	2.98E-03	1.62E+00	2.70E-02
KIAA0319	NM_001350407	4.81E-03	1.61E+00	3.61E-02
KIAA0586	NM_001364701	1.73E-05	7.96E+00	1.39E-03
KIAA0754	NM_015038	2.74E-04	-1.86E+00	6.71E-03
KIAA0825	NM_001145678	6.96E-03	-1.84E+00	4.54E-02
KIAA1109	NM_015312	7.64E-05	-1.79E+00	3.15E-03
KIAA1191	NM_001287336	2.20E-04	1.82E+00	5.95E-03
KIAA1841	NM_001330436	2.63E-03	-8.64E+00	2.52E-02
KIAA2026	NM_001017969	9.73E-06	-1.81E+00	1.03E-03
KIDINS220	NM_020738	5.77E-06	-1.89E+00	8.21E-04
KIF18A	NM_031217	5.77E-03	-1.79E+00	4.04E-02
KIF20B	NM_016195	6.59E-03	-1.68E+00	4.39E-02
KIF21A	NM_001173463	5.35E-03	-2.63E+00	3.87E-02
KIF27	NM_017576	4.19E-03	-3.16E+00	3.30E-02
KIF2A	NM_004520	1.97E-04	-1.94E+00	5.61E-03
KIF5B	NM_004521	5.45E-04	-1.53E+00	9.81E-03

KIFAP3	NM_014970	2.33E-05	-1.79E+00	1.66E-03
KIFC2	NM_001369769	9.45E-04	2.34E+00	1.35E-02
KIR2DS4	NM_001281971	2.58E-04	-4.89E+00	6.54E-03
KLF12	NM_007249	3.10E-03	-1.97E+00	2.75E-02
KLF3	NM_016531	8.25E-05	-1.58E+00	3.32E-03
KLF5	NM_001286818	6.19E-04	-2.61E+00	1.05E-02
KLF8	NR_136704	2.63E-04	-3.15E+00	6.62E-03
KLF9	NM_001206	2.56E-04	-2.58E+00	6.52E-03
KLHDC3	NR_040101	1.15E-03	4.03E+00	1.52E-02
KLHDC8B	NM_173546	3.04E-03	1.66E+00	2.73E-02
KLHL14	NM_020805	6.93E-03	-1.98E+00	4.52E-02
KLHL24	NM_001349423	4.36E-04	-1.81E+00	8.63E-03
KLHL28	NM_017658	1.65E-03	-1.50E+00	1.91E-02
KLHL5	NM_001007075	4.47E-03	-1.59E+00	3.44E-02
KLHL8	NM_020803	5.33E-03	-3.44E+00	3.86E-02
KLRC3	NM_007333	8.80E-06	-3.53E+00	9.77E-04
KLRC4	NM_013431	4.18E-04	-2.43E+00	8.40E-03
KLRD1	NR_147038	3.61E-06	-2.38E+00	6.85E-04
KMT2A	NM_001197104	2.41E-03	-1.53E+00	2.39E-02
KMT2C	NM_170606	6.73E-07	-1.83E+00	3.69E-04
KMT2E-AS1	NR_024586	6.35E-03	1.86E+00	4.28E-02
KMT5A	NM_001324506	2.68E-03	2.28E+00	2.55E-02
KMT5B	NM_017635	3.54E-04	-1.52E+00	7.75E-03
KNL1	NM_144508	5.98E-03	-1.80E+00	4.13E-02
KNOP1	NM_001348534	7.77E-03	-2.78E+00	4.86E-02
KPNA5	NM_002269	4.59E-03	-2.69E+00	3.49E-02
KRIT1	NM_001350684	1.16E-05	-2.95E+00	1.11E-03
KRT17P1	NR_146392	2.16E-03	1.72E+00	2.23E-02
KRT72	NM_001146225	2.42E-04	4.26E+00	6.32E-03
KRTCAP2	NM_173852	3.61E-04	1.53E+00	7.83E-03
KXD1	NM_024069	6.68E-04	1.80E+00	1.10E-02
KYAT1	NR_148224	1.31E-03	1.67E+00	1.65E-02
L3MBTL3	NM_001007102	6.17E-03	-1.51E+00	4.20E-02
LACC1	NM_001350640	4.99E-03	-2.46E+00	3.71E-02
LAGE3	NM_006014	7.52E-03	2.28E+00	4.77E-02
LAIR1	NM_001289026	1.84E-06	1.68E+00	4.95E-04
LAMP2	NM_001122606	4.86E-03	3.99E+00	3.63E-02
LAMTOR1	NM_017907	6.00E-04	1.63E+00	1.03E-02

LAMTOR2	NM_014017	7.20E-03	1.94E+00	4.65E-02
LARP1B	NM_001278604	2.09E-03	-1.53E+00	2.19E-02
LARP4	NM_001330415	7.75E-07	-3.27E+00	3.69E-04
LARS	NM_020117	1.30E-03	-1.50E+00	1.64E-02
LATS1	NM_004690	2.31E-06	-2.44E+00	5.56E-04
LBHD1	NM_001367940	6.24E-06	1.87E+00	8.32E-04
LBR	NM_002296	6.15E-03	-1.52E+00	4.19E-02
LCAT	NM_000229	2.67E-03	2.49E+00	2.54E-02
LCLAT1	NM_182551	1.16E-03	-2.25E+00	1.53E-02
LCOR	NM_032440	8.26E-08	-3.45E+00	1.45E-04
LCORL	NM_001166139	3.22E-05	-2.98E+00	1.99E-03
LDLRAP1	NM_015627	7.40E-03	1.65E+00	4.73E-02
LEMD2	NM_001143944	8.57E-04	1.53E+00	1.28E-02
LEMD3	NM_014319	9.93E-07	-2.00E+00	3.95E-04
LETM2	NM_001199659	2.50E-03	2.18E+00	2.45E-02
LETMD1	NR_147117	4.83E-04	3.10E+00	9.16E-03
LGALS1	NM_002305	3.05E-03	2.69E+00	2.73E-02
LGALS2	NM_006498	6.70E-03	3.02E+00	4.43E-02
LGALS3	NR_003225	3.39E-03	1.50E+00	2.91E-02
LIG1	NR_135499	4.04E-04	2.43E+00	8.31E-03
LIG4	NM_002312	6.34E-04	-2.36E+00	1.07E-02
LILRA6	NR_104098	6.25E-03	1.75E+00	4.24E-02
LILRB1	NM_006669	5.75E-03	-1.64E+00	4.03E-02
LILRB2	NR_103521	1.40E-03	2.14E+00	1.72E-02
LILRB3	NR_135495	4.20E-03	1.92E+00	3.30E-02
LIMA1	NM_001113546	2.80E-03	-2.48E+00	2.61E-02
LIMS1	NM_001193484	1.66E-04	-2.00E+00	5.03E-03
LINC00528	NR_103718	1.64E-03	1.69E+00	1.90E-02
LINC00638	NR_024396	1.76E-03	1.69E+00	1.99E-02
LINC00663	NR_026956	2.28E-04	1.86E+00	6.08E-03
LINC00671	NR_027254	6.06E-03	1.72E+00	4.15E-02
LINC00989	NR_038826	4.66E-04	-2.54E+00	8.98E-03
LINC01004	NR_039981	5.80E-04	-1.51E+00	1.01E-02
LINC01137	NR_038842	2.80E-04	2.27E+00	6.79E-03
LINC01503	NR_120685	4.45E-04	2.87E+00	8.73E-03
LINC01550	NR_152748	5.13E-03	1.87E+00	3.76E-02
LINC01569	NR_039999	1.50E-03	1.76E+00	1.80E-02
LINC01578	NR_037600	3.67E-03	1.66E+00	3.05E-02

LINC01624	NR_104177	5.08E-03	1.51E+00	3.74E-02
LINC01730	NR_109859	3.08E-04	2.25E+00	7.18E-03
LINC01881	NR_130701	2.63E-03	-1.54E+00	2.52E-02
LINC02012	NR_145451	2.59E-03	1.88E+00	2.51E-02
LINC02218	NR_134270	2.02E-03	-2.10E+00	2.15E-02
LINC02470	NR_104634	7.33E-05	5.13E+00	3.07E-03
LMBR1	NR_146959	3.12E-05	-1.55E+00	1.97E-03
LMF1	NR_036442	9.47E-04	2.99E+00	1.35E-02
LNPEP	NM_005575	4.95E-06	-1.63E+00	7.70E-04
LNPK	NM_030650	4.05E-04	-1.83E+00	8.31E-03
LOC100190986	NR_024456.2	6.29E-03	-1.51E+00	4.25E-02
LOC100288069	NR_033908	1.85E-03	-1.59E+00	2.05E-02
LOC100294145	NR_037178	3.26E-04	-2.21E+00	7.43E-03
LOC100505622	NR_038332	1.19E-03	1.63E+00	1.55E-02
LOC100506100	NR_046240	2.48E-03	1.87E+00	2.43E-02
LOC100506476	NR_109995	8.09E-04	1.87E+00	1.23E-02
LOC100652768	NR_045215	1.17E-03	2.20E+00	1.54E-02
LOC101926963	NR_110054	5.72E-03	1.99E+00	4.03E-02
LOC101927596	NR_135584	1.39E-03	2.16E+00	1.72E-02
LOC101928034	NR_125947	2.96E-03	1.77E+00	2.69E-02
LOC101929243	NR_134626	9.39E-04	1.70E+00	1.35E-02
LOC101929599	NR_157804.2	2.58E-03	1.59E+01	2.50E-02
LOC101929698	NR_110619	1.61E-04	2.80E+00	4.92E-03
LOC102724814	NR_110555	7.02E-04	1.50E+00	1.13E-02
LOC102725121	NR_148357	2.60E-05	3.98E+00	1.76E-03
LOC105371795	NR_135646	3.23E-05	2.76E+00	1.99E-03
LOC110384692	NM_001352000	1.15E-03	3.33E+00	1.52E-02
LOC171391	NR_126343	9.31E-06	2.01E+00	1.01E-03
LOC284581	NR_046097	5.73E-03	4.34E+00	4.03E-02
LOC339192	NR_147507	2.35E-04	1.96E+00	6.18E-03
LOC646214	NR_027053	1.44E-05	-1.87E+00	1.25E-03
LOC728392	NM_001162371	4.92E-04	1.56E+00	9.23E-03
LONP2	NM_031490	8.37E-05	-1.62E+00	3.37E-03
LONRF1	NM_152271	1.99E-03	-1.71E+00	2.13E-02
LPAR2	NM_004720	1.46E-03	1.91E+00	1.77E-02
LPL	NM_000237	3.99E-04	-6.76E+00	8.27E-03
LRBA	NM_001199282	2.16E-05	-3.27E+00	1.59E-03
LRCH1	NM_001164213	5.56E-05	-1.52E+00	2.61E-03

LRCH3	NM_001365716	5.45E-04	-1.84E+00	9.81E-03
LRIF1	NM_018372	4.82E-06	-1.94E+00	7.54E-04
LRMDA	NM_032024	4.45E-04	1.64E+00	8.73E-03
LRRC23	NM_201650	6.16E-03	1.84E+00	4.20E-02
LRRC6	NR_135906	4.18E-05	4.64E+00	2.22E-03
LRRCC1	NM_001349636	1.78E-03	-3.00E+00	2.00E-02
LRRFIP1	NM_001137553	1.15E-05	-1.64E+00	1.11E-03
LRRK2	NM_198578	7.46E-04	-1.83E+00	1.18E-02
LRSAM1	NM_001005374	2.36E-03	2.10E+00	2.36E-02
LSM1	NM_014462	5.42E-03	1.55E+00	3.90E-02
LSM10	NM_032881	6.18E-04	2.06E+00	1.05E-02
LSM4	NM_012321	1.47E-04	1.79E+00	4.66E-03
LSS	NM_001145436	6.33E-03	1.69E+00	4.27E-02
LST1	NM_001166538	3.07E-04	4.30E+00	7.18E-03
LTA4H	NM_001256644	7.64E-03	1.57E+00	4.81E-02
LTB	NM_009588	2.28E-03	2.18E+00	2.32E-02
LTBP3	NM_001164266	5.37E-03	2.12E+00	3.87E-02
LTN1	NM_015565	2.09E-04	-1.85E+00	5.76E-03
LTV1	NM_001329953	5.14E-03	-1.59E+00	3.77E-02
LUC7L	NM_001320226	1.87E-03	2.21E+00	2.06E-02
LUZP6	NM_001128619	1.25E-04	-1.61E+00	4.23E-03
LY6G5C	NM_025262	2.92E-04	1.57E+00	6.93E-03
LY75	NM_002349	6.34E-04	-1.67E+00	1.07E-02
LY75-CD302	NM_001198759	1.72E-04	-8.97E+00	5.14E-03
LY86	NM_004271	8.95E-04	1.59E+00	1.31E-02
LYRM4	NM_001164840	1.13E-03	1.85E+00	1.50E-02
LYRM9	NM_001076680	1.94E-03	1.52E+00	2.11E-02
LYSMD3	NM_198273	6.74E-04	-2.04E+00	1.10E-02
LYST	NM_001301365	2.10E-04	-1.59E+00	5.77E-03
MACROD2	NM_001033087	2.85E-04	-2.12E+00	6.83E-03
MAD2L2	NM_006341	2.63E-03	2.16E+00	2.52E-02
MAEA	NM_005882	5.74E-03	2.92E+00	4.03E-02
MAF	NM_001031804	8.44E-04	-1.66E+00	1.26E-02
MAGED2	NM_177433	3.47E-03	1.56E+00	2.95E-02
MAGEE1	NM_020932	1.82E-03	-1.65E+00	2.03E-02
MAK	NR_134935	9.48E-05	-3.89E+00	3.66E-03
MAML2	NM_032427	2.66E-04	-1.58E+00	6.65E-03
MAN1B1	NR_045720	6.22E-05	1.79E+00	2.78E-03

MAN2C1	NM_001256496	2.77E-03	2.64E+00	2.60E-02
MANBAL	NM_001003897	1.41E-04	1.59E+00	4.54E-03
MAP3K1	NM_005921	2.76E-05	-1.73E+00	1.82E-03
MAP3K12	NM_006301	3.83E-04	1.53E+00	8.07E-03
MAP3K6	NM_004672	3.88E-03	2.13E+00	3.16E-02
MAP4K5	NM_198794	5.20E-06	-1.95E+00	7.87E-04
MAPK11	NM_002751	6.37E-03	2.21E+00	4.29E-02
MAPK8	NM_001323321	1.95E-03	-1.54E+00	2.11E-02
MAPKAPK5-AS1	NR_152606	8.65E-07	1.52E+00	3.76E-04
MARCHF6	NM_001270661	8.89E-05	2.11E+00	3.50E-03
MARCHF7	NM_001282805	6.42E-04	-1.81E+00	1.08E-02
MARCHF9	NM_138396	5.76E-03	1.67E+00	4.04E-02
MARCO	NM_006770	6.32E-03	2.12E+00	4.27E-02
MARF1	NM_014647	6.76E-06	-1.54E+00	8.49E-04
MAST4	NM_001297651	3.04E-03	-2.42E+00	2.73E-02
MAX	NM_145114	2.07E-03	2.24E+00	2.17E-02
MBNL1	NM_207297	7.41E-07	-2.63E+00	3.69E-04
MBNL2	NM_207304	1.10E-04	-2.04E+00	4.00E-03
MBOAT1	NR_073465	1.30E-05	2.11E+00	1.20E-03
MCC	NM_002387	3.58E-03	-1.72E+00	3.01E-02
MCFD2	NM_001171511	5.13E-03	-1.52E+00	3.77E-02
MCM7	NM_005916	9.19E-04	1.84E+00	1.33E-02
MCPH1	NM_001363979	1.79E-03	-2.66E+00	2.01E-02
MDH2	NR_104165	1.87E-03	1.94E+00	2.06E-02
MDM2	NM_002392	1.76E-06	-3.37E+00	4.95E-04
MDM4	NM_001278516	2.06E-04	-1.86E+00	5.72E-03
MDP1	NM_138476	4.04E-04	2.39E+00	8.31E-03
MEA1	NM_001318942	7.82E-05	1.61E+00	3.22E-03
MED11	NM_001001683	5.54E-04	1.88E+00	9.87E-03
MED12L	NM_053002	2.93E-05	-2.02E+00	1.90E-03
MED13	NM_005121	3.99E-06	-1.71E+00	7.03E-04
MED13L	NM_015335	1.02E-06	-2.05E+00	3.95E-04
MED19	NM_001317078	7.32E-04	1.61E+00	1.16E-02
MEF2A	NM_001130927	1.39E-04	2.46E+00	4.52E-03
MEF2C	NM_001364340	7.99E-04	-1.96E+00	1.22E-02
MEGF6	NM_001409	6.92E-04	1.90E+00	1.12E-02
MEPCE	NM_001363486	1.68E-03	1.64E+00	1.93E-02
METAP1D	NR_136276	3.60E-03	2.76E+00	3.01E-02

METTL1	NM_005371	1.11E-05	1.91E+00	1.10E-03
METTL17	NM_001029991	1.01E-05	1.59E+00	1.05E-03
METTL23	NM_001080510	5.42E-03	2.29E+00	3.90E-02
METTL26	NR_109976	8.41E-04	2.26E+00	1.26E-02
METTL7B	NM_152637	2.19E-03	6.89E+00	2.26E-02
MEX3C	NM_016626	5.48E-06	-2.04E+00	8.07E-04
MFAP3	NM_005927	2.46E-05	-2.14E+00	1.71E-03
MFF	NM_001277068	7.61E-03	-2.67E+00	4.80E-02
MFSD6	NM_017694	1.86E-05	-1.52E+00	1.44E-03
MGA	NM_001164273	2.65E-04	-2.17E+00	6.62E-03
MGAT1	NM_001114619	1.00E-03	1.80E+00	1.40E-02
MGAT4A	NM_012214	5.76E-04	-1.58E+00	1.01E-02
MGAT4B	NM_054013	2.77E-03	1.58E+00	2.60E-02
MGRN1	NM_001142291	3.70E-03	2.56E+00	3.07E-02
MGST3	NM_004528	1.33E-06	2.04E+00	4.43E-04
MHENCR	NR_132417	2.31E-04	2.08E+00	6.14E-03
MIA3	NM_198551	3.27E-04	-1.96E+00	7.44E-03
MIB1	NM_020774	9.70E-05	-1.75E+00	3.72E-03
MICAL1	NM_001159291	1.80E-04	2.07E+00	5.29E-03
MICOS13	NM_001365761	3.92E-04	2.32E+00	8.20E-03
MICU1	NM_001195519	6.94E-03	9.33E+00	4.53E-02
MID2	NM_012216	1.11E-03	1.91E+00	1.48E-02
MIEN1	NM_001330206	2.48E-06	2.00E+00	5.67E-04
MIER1	NM_001077701	3.28E-04	-1.72E+00	7.46E-03
MIGA1	NM_001270384	3.16E-04	-1.99E+00	7.29E-03
MIGA2	NR_138421	6.02E-03	1.73E+00	4.14E-02
MILR1	NM_001291316	2.02E-03	2.18E+00	2.15E-02
MIR4435-2HG	NR_024373	6.02E-03	2.07E+00	4.14E-02
MIR570HG	NR_122105	2.93E-03	1.57E+00	2.68E-02
MIRLET7BHG	NR_110479	4.52E-03	2.28E+00	3.46E-02
MKI67	NM_002417	1.93E-03	-2.17E+00	2.10E-02
MKLN1	NM_001321316	2.83E-03	-1.56E+00	2.63E-02
MLLT10	NM_001195626	4.80E-05	-1.52E+00	2.42E-03
MLST8	NM_001352060	1.58E-03	2.78E+00	1.86E-02
MMAB	NM_052845	2.61E-03	1.89E+00	2.52E-02
MMD	NM_012329	4.36E-05	-2.16E+00	2.28E-03
MME	NM_007289	3.85E-03	-1.70E+00	3.14E-02
MMGT1	NM_173470	3.24E-03	-1.68E+00	2.83E-02

MMS22L	NM_001350599	5.15E-05	-1.93E+00	2.54E-03
MOB1A	NM_001317112	4.04E-04	2.38E+00	8.31E-03
MOCS2	NM_004531	2.81E-03	-2.53E+00	2.61E-02
MON2	NM_001278469	3.71E-03	-1.84E+00	3.07E-02
MORC2	NM_001303257	2.95E-03	1.90E+00	2.69E-02
MORC3	NM_015358	4.03E-06	-1.88E+00	7.03E-04
MORN2	NM_001145450	3.41E-03	1.84E+00	2.92E-02
MOSPD2	NM_001177475	5.11E-04	-3.49E+00	9.44E-03
MPDU1	NM_004870	2.83E-05	1.65E+00	1.85E-03
MPHOSPH9	NM_022782	1.93E-03	-1.59E+00	2.10E-02
MPI	NM_002435	7.66E-03	1.52E+00	4.82E-02
MPP5	NM_022474	4.05E-03	-1.89E+00	3.23E-02
MPV17L2	NM_032683	7.84E-03	2.64E+00	4.89E-02
MPZ	NM_000530	4.05E-04	1.77E+00	8.31E-03
MPZL3	NR_104405	4.78E-05	-1.89E+00	2.41E-03
MR1	NM_001195035	8.12E-05	-2.64E+00	3.30E-03
MRM3	NM_001317947	5.33E-04	2.83E+00	9.69E-03
MRPL11	NM_170738	4.33E-05	2.56E+00	2.27E-03
MRPL14	NM_032111	3.26E-03	1.99E+00	2.84E-02
MRPL17	NM_022061	4.09E-04	1.51E+00	8.33E-03
MRPL20	NM_001318485	2.63E-03	1.67E+00	2.53E-02
MRPL21	NM_181514	2.09E-06	1.97E+00	5.21E-04
MRPL22	NM_014180	5.27E-04	1.77E+00	9.63E-03
MRPL23	NM_021134	6.81E-03	2.80E+00	4.48E-02
MRPL24	NM_024540	2.60E-04	1.62E+00	6.58E-03
MRPL27	NM_016504	1.34E-04	1.93E+00	4.45E-03
MRPL34	NM_023937	5.03E-03	1.55E+00	3.72E-02
MRPL36	NM_032479	3.08E-03	1.61E+00	2.75E-02
MRPL40	NM_001318151	3.84E-03	2.38E+00	3.14E-02
MRPL43	NM_032112	2.87E-03	1.53E+00	2.64E-02
MRPL46	NM_022163	1.86E-05	1.63E+00	1.44E-03
MRPL48	NM_016055	5.60E-03	1.56E+00	3.98E-02
MRPL52	NM_181306	6.54E-04	2.19E+00	1.09E-02
MRPL53	NM_053050	1.97E-03	2.75E+00	2.12E-02
MRPL54	NM_172251	7.51E-03	2.65E+00	4.77E-02
MRPL55	NM_181465	2.65E-04	3.31E+00	6.62E-03
MRPL58	NM_001545	4.54E-07	1.78E+00	3.14E-04
MRPS11	NM_022839	2.45E-03	1.70E+00	2.41E-02

MRPS15	NM_031280	2.71E-05	1.59E+00	1.81E-03
MRPS18A	NM_018135	2.25E-03	1.85E+00	2.29E-02
MRPS21	NM_031901	4.14E-05	2.22E+00	2.22E-03
MRPS22	NM_020191	2.98E-03	1.65E+00	2.70E-02
MRPS24	NM_032014	4.50E-05	1.60E+00	2.32E-03
MRPS26	NM_030811	2.68E-03	1.67E+00	2.55E-02
MRPS27	NM_001286748	1.66E-03	2.38E+00	1.92E-02
MRPS34	NM_001300900	1.91E-03	2.79E+00	2.08E-02
MRTO4	NM_016183	1.69E-04	1.54E+00	5.08E-03
MS4A1	NM_021950	4.03E-03	-1.61E+00	3.23E-02
MS4A14	NM_001079692	1.87E-03	1.99E+00	2.06E-02
MS4A6A	NM_152851	7.78E-03	1.56E+00	4.87E-02
MSANTD2	NM_001312921	1.52E-05	4.25E+00	1.29E-03
MSANTD4	NM_032424	1.78E-05	-2.39E+00	1.41E-03
MSH3	NM_002439	4.12E-04	-1.55E+00	8.35E-03
MSH5-SAPCD1	NR_037846	1.13E-03	1.63E+00	1.51E-02
MSH6	NM_000179	3.01E-03	-1.74E+00	2.72E-02
MSL2	NM_001145417	2.86E-03	-1.51E+00	2.64E-02
MST1P2	NR_027504	2.29E-03	2.41E+00	2.32E-02
MT1F	NM_005949	7.56E-03	2.06E+00	4.79E-02
MTA3	NM_001330444	2.67E-03	2.76E+00	2.54E-02
MTBP	NM_022045	5.98E-03	-1.67E+00	4.13E-02
MTCH1	NM_001271641	1.44E-03	1.54E+00	1.75E-02
MTDH	NM_178812	2.23E-03	-1.66E+00	2.28E-02
MTERF4	NR_028051	7.64E-03	2.13E+00	4.81E-02
MTF2	NM_007358	4.97E-05	-1.68E+00	2.47E-03
MTM1	NM_000252	1.23E-04	-1.50E+00	4.22E-03
MTMR1	NM_001353991	3.57E-03	-1.63E+00	3.00E-02
MTMR12	NM_001040446	8.93E-06	-1.86E+00	9.81E-04
MTMR2	NM_001243571	6.93E-05	-3.19E+00	2.97E-03
MTMR6	NM_004685	2.86E-03	-1.57E+00	2.64E-02
MTPN	NM_145808	1.25E-04	-1.61E+00	4.23E-03
MTR	NM_000254	3.09E-04	-2.07E+00	7.19E-03
MTRF1	NM_001354076	3.69E-03	-1.81E+00	3.06E-02
MTSS1	NM_001363300	1.58E-03	-1.61E+00	1.85E-02
MTX1	NM_002455	2.63E-03	1.97E+00	2.52E-02
MYBL1	NM_001080416	5.74E-03	-2.49E+00	4.03E-02
MYCBP2	NM_015057	8.45E-06	-1.79E+00	9.51E-04

MYDGF	NM_019107	5.49E-03	1.77E+00	3.93E-02
MYL12A	NM_006471	5.76E-03	1.53E+00	4.04E-02
MYL6	NM_079423	4.99E-04	2.22E+00	9.29E-03
MYL6B	NM_002475	1.67E-03	2.33E+00	1.92E-02
MYLK	NM_053026	7.97E-03	-1.70E+00	4.95E-02
MYO1B	NM_012223	2.89E-04	-3.45E+00	6.88E-03
MYO6	NM_001368137	4.89E-04	-2.24E+00	9.20E-03
MYSM1	NM_001085487	5.11E-04	-1.60E+00	9.44E-03
N4BP2	NM_001318359	2.94E-03	-1.65E+00	2.68E-02
NAA10	NM_003491	9.71E-04	1.99E+00	1.37E-02
NAA35	NM_001321882	1.30E-03	-2.03E+00	1.64E-02
NAA50	NM_025146	3.30E-05	-1.54E+00	2.01E-03
NAAA	NM_001042402	1.64E-04	1.72E+00	4.99E-03
NAGK	NM_017567	5.55E-03	1.61E+00	3.96E-02
NANS	NM_018946	3.60E-03	1.53E+00	3.01E-02
NAPEPLD	NM_001122838	3.03E-03	-1.84E+00	2.73E-02
NARF	NM_031968	4.89E-03	1.51E+00	3.65E-02
NAT9	NR_130953	1.35E-03	1.92E+00	1.69E-02
NAXD	NM_001242883	7.88E-03	2.92E+00	4.91E-02
NAXE	NM_144772	2.90E-04	1.55E+00	6.90E-03
NBAS	NR_052013	1.41E-03	-2.38E+00	1.73E-02
NBEA	NM_015678	7.25E-03	-1.92E+00	4.67E-02
NBEAL1	NM_001114132	1.89E-03	-1.84E+00	2.07E-02
NBL1	NM_001278166	1.04E-03	3.62E+00	1.43E-02
NBR2	NR_003108	1.07E-05	1.61E+00	1.07E-03
NCALD	NM_001040625	3.48E-03	-2.67E+00	2.96E-02
NCAM1	NM_000615	9.03E-04	-1.69E+00	1.31E-02
NCBP2AS2	NM_001355243	1.84E-05	1.78E+00	1.44E-03
NCF4	NM_013416	1.10E-03	2.60E+00	1.48E-02
NCK1	NM_001291999	3.70E-03	-1.57E+00	3.07E-02
NCKAP1	NM_013436	2.48E-04	-1.95E+00	6.39E-03
NCKAP1L	NM_001184976	8.41E-04	1.76E+00	1.26E-02
NCOA1	NM_001362952	5.02E-05	-1.69E+00	2.49E-03
NCOA2	NM_006540	6.37E-08	-2.28E+00	1.42E-04
NCOA3	NM_181659	6.60E-06	-1.55E+00	8.39E-04
NCOA4	NM_001145263	1.09E-03	-1.51E+00	1.47E-02
NCOA7	NM_001122842	1.52E-05	-2.87E+00	1.29E-03
NCR1	NM_004829	4.76E-03	-1.88E+00	3.58E-02

NCR3LG1	NM_001202439	2.28E-04	-2.14E+00	6.08E-03
NCSTN	NM_001290186	2.56E-03	1.68E+00	2.49E-02
NDN	NM_002487	4.82E-05	2.76E+00	2.42E-03
NDOR1	NM_001144027	2.94E-03	2.33E+00	2.68E-02
NDUFA2	NM_002488	2.02E-04	1.83E+00	5.68E-03
NDUFA4	NM_002489	2.23E-03	1.95E+00	2.28E-02
NDUFA7	NM_005001	3.30E-03	2.26E+00	2.86E-02
NDUFA8	NM_014222	3.97E-05	1.55E+00	2.20E-03
NDUFAF1	NR_045620	2.00E-04	1.69E+00	5.66E-03
NDUFAF2	NM_174889	9.55E-04	1.61E+00	1.36E-02
NDUFAF7	NM_144736	1.92E-03	2.58E+00	2.09E-02
NDUFAF8	NR_148426	5.95E-03	1.70E+00	4.12E-02
NDUFB1	NM_004545	4.17E-03	1.65E+00	3.29E-02
NDUFB10	NM_004548	1.84E-04	1.70E+00	5.36E-03
NDUFB11	NM_019056	7.24E-04	2.10E+00	1.16E-02
NDUFB2	NM_004546	1.12E-03	1.92E+00	1.49E-02
NDUFB8	NM_001284367	2.73E-06	1.69E+00	5.85E-04
NDUFB9	NM_005005	1.89E-05	1.59E+00	1.46E-03
NDUFS1	NM_001199984	4.61E-04	-1.53E+00	8.94E-03
NDUFS3	NM_004551	2.90E-06	1.82E+00	5.95E-04
NDUFV1	NM_001166102	5.16E-03	3.38E+00	3.77E-02
NEBL	NM_213569	2.30E-04	2.52E+01	6.11E-03
NECAB2	NM_001329749	9.97E-04	3.37E+00	1.40E-02
NECAB3	NM_031231	2.08E-03	2.57E+00	2.18E-02
NEDD1	NM_152905	5.78E-04	-2.72E+00	1.01E-02
NEDD9	NM_001142393	7.88E-03	-2.06E+00	4.91E-02
NEIL1	NM_001256552	3.11E-03	1.74E+00	2.76E-02
NEK1	NM_001199400	3.09E-03	-2.29E+00	2.75E-02
NEK4	NM_001348413	1.47E-03	-1.98E+00	1.77E-02
NEK7	NM_133494	2.46E-04	-1.77E+00	6.36E-03
NELFE	NM_002904	1.79E-03	1.58E+00	2.01E-02
NELL2	NM_001145110	2.58E-03	1.99E+00	2.50E-02
NENF	NM_013349	5.85E-06	2.12E+00	8.22E-04
NEXN	NM_001172309	4.37E-03	-2.24E+00	3.38E-02
NF1	NM_000267	5.76E-06	-1.79E+00	8.21E-04
NFAT5	NM_006599	2.39E-04	-1.52E+00	6.24E-03
NFE2	NM_001261461	5.94E-03	1.67E+00	4.11E-02
NFKB2	NM_001322934	4.94E-03	1.67E+00	3.68E-02

NFKBID	NM_032721	2.51E-05	2.48E+00	1.73E-03
NFKBIE	NM_004556	1.79E-03	1.58E+00	2.01E-02
NFKBIL1	NM_001144961	6.65E-03	2.22E+00	4.41E-02
NFYA	NM_021705	6.87E-05	-1.52E+00	2.96E-03
NHLRC2	NM_198514	7.99E-05	-1.60E+00	3.25E-03
NHS	NM_001136024	6.75E-06	-2.25E+00	8.49E-04
NIF3L1	NM_001142355	5.59E-03	2.08E+00	3.97E-02
NIPAL2	NR_135746	8.96E-04	-3.25E+00	1.31E-02
NIPBL	NM_015384	5.31E-08	-1.75E+00	1.37E-04
NLGN2	NM_020795	4.41E-04	2.21E+00	8.67E-03
NLGN3	NM_001321276	1.08E-03	1.86E+00	1.46E-02
NLK	NM_016231	5.36E-06	-1.51E+00	7.98E-04
NLRP1	NM_033004	2.06E-03	1.54E+00	2.17E-02
NLRP3	NM_001243133	9.18E-04	-2.05E+00	1.33E-02
NME1	NM_000269	1.92E-04	1.70E+00	5.51E-03
NME4	NM_001286440	4.88E-03	2.27E+00	3.65E-02
NME6	NM_001308433	6.22E-03	-1.81E+00	4.23E-02
NMRK1	NM_001127603	2.86E-06	5.36E+00	5.95E-04
NNT-AS1	NR_073113	3.04E-03	-1.53E+00	2.73E-02
NOL12	NM_024313	2.07E-03	1.84E+00	2.17E-02
NOP10	NM_018648	5.33E-04	1.77E+00	9.69E-03
NOP16	NM_016391	2.51E-03	1.55E+00	2.45E-02
NOP2	NM_006170	7.00E-03	1.57E+00	4.55E-02
NOP53	NM_015710	6.99E-03	2.17E+00	4.55E-02
NOP56	NR_027700	3.10E-03	1.92E+00	2.75E-02
NORAD	NR_027451	3.84E-05	-1.54E+00	2.18E-03
NOTCH4	NR_134950	2.14E-03	1.67E+00	2.22E-02
NPAT	NM_001321307	5.10E-03	-1.66E+00	3.75E-02
NPFF	NM_001320296	7.36E-05	2.15E+00	3.08E-03
NPIPA5	NM_001277325	7.62E-03	-1.65E+00	4.81E-02
NPIPA7	NM_001282507.1	1.25E-05	2.12E+00	1.16E-03
NPIPB12	NM_001355401	6.86E-03	1.75E+00	4.50E-02
NPM3	NM_006993	2.67E-03	2.39E+00	2.54E-02
NPRL2	NM_006545	6.88E-04	1.56E+00	1.12E-02
NPTN-IT1	NR_103844	1.28E-03	-1.81E+00	1.63E-02
NR1D2	NM_005126	5.65E-03	-1.73E+00	4.00E-02
NR1H3	NM_001130102	4.65E-03	2.49E+00	3.53E-02
NR2C2	NM_001291694	3.90E-05	-1.54E+00	2.19E-03

NR2C2AP	NM_176880	3.26E-03	1.75E+00	2.84E-02
NR2F6	NM_005234	1.35E-03	2.64E+00	1.69E-02
NR3C1	NM_001204258	4.17E-06	-3.49E+00	7.03E-04
NR3C2	NR_148974	1.21E-03	2.44E+00	1.57E-02
NRADDP	NR_024046	2.29E-05	2.85E+00	1.64E-03
NRIP1	NM_003489	3.13E-04	-1.58E+00	7.24E-03
NRM	NM_001270710	3.02E-03	1.51E+00	2.72E-02
NRN1	NM_016588	7.90E-04	-3.01E+00	1.21E-02
NSD1	NM_022455	1.82E-04	-1.88E+00	5.33E-03
NSD3	NM_023034	1.74E-05	-1.64E+00	1.39E-03
NSFL1C	NR_038164	1.77E-03	2.42E+00	2.00E-02
NSG1	NM_014392	1.53E-03	2.04E+00	1.81E-02
NSMCE2	NM_173685	3.83E-03	1.65E+00	3.13E-02
NSUN5P1	NR_104013	3.36E-03	2.04E+00	2.89E-02
NT5C	NR_045513	4.77E-03	2.20E+00	3.59E-02
NT5C2	NM_001351182	4.90E-04	-2.26E+00	9.22E-03
NUAK1	NM_014840	2.06E-03	-3.32E+00	2.17E-02
NUDT1	NM_198953	6.03E-03	2.09E+00	4.14E-02
NUDT14	NM_177533	5.16E-03	2.71E+00	3.77E-02
NUDT16	NM_001171905	8.92E-04	-5.09E+00	1.31E-02
NUFIP2	NM_020772	1.84E-03	-1.51E+00	2.04E-02
NUMA1	NR_104476	5.86E-04	3.29E+00	1.02E-02
NUMB	NM_001320114	7.98E-03	-1.59E+00	4.95E-02
NUP153	NM_005124	4.43E-05	-2.05E+00	2.30E-03
NUP155	NM_153485	2.68E-03	-1.52E+00	2.54E-02
NUP160	NM_015231	1.21E-04	-1.97E+00	4.20E-03
NUP205	NM_015135	5.66E-04	-1.56E+00	1.00E-02
NUP214	NM_001318324	6.66E-04	-1.59E+00	1.10E-02
NUP43	NR_104456	7.38E-03	-4.12E+00	4.72E-02
NUP58	NM_014089	1.85E-03	-1.56E+00	2.04E-02
NUP98	NR_157589	8.54E-05	-1.66E+00	3.41E-03
NUTM2A-AS1	NR_024397	5.51E-03	1.58E+00	3.94E-02
NXT1	NM_013248	3.56E-05	1.65E+00	2.10E-03
OBSCN	NM_001098623	6.34E-03	2.30E+00	4.28E-02
OCM	NM_001097622	3.08E-03	1.56E+00	2.75E-02
ODF2L	NM_001366783	2.48E-04	-1.61E+00	6.39E-03
OGA	NM_012215	5.73E-04	-1.59E+00	1.01E-02
OGFOD2	NM_001304834	3.86E-03	2.62E+00	3.15E-02

OPA1	NM_015560	5.77E-04	-1.77E+00	1.01E-02
OPRL1	NM_001318855	4.98E-03	1.80E+00	3.70E-02
OR7D2	NM_175883	1.23E-05	2.34E+01	1.15E-03
OS9	NM_001261423	5.36E-03	2.64E+00	3.87E-02
OSBPL10	NM_017784	1.05E-03	-2.62E+00	1.44E-02
OSBPL1A	NM_080597	1.53E-03	-1.70E+00	1.82E-02
OSBPL6	NM_001201482	2.62E-04	-2.92E+00	6.60E-03
OSBPL8	NM_001319652	3.25E-04	-1.80E+00	7.41E-03
OSCAR	NM_206818	1.77E-04	3.10E+00	5.25E-03
OSGEP	NM_017807	2.11E-06	1.54E+00	5.23E-04
OSGIN2	NM_004337	5.19E-03	-1.58E+00	3.79E-02
OTUB1	NR_003089	2.11E-03	2.07E+00	2.20E-02
OTUD4	NM_001102653	2.67E-04	-1.53E+00	6.65E-03
OVCA2	NM_080822	2.85E-05	1.92E+00	1.85E-03
OVGP1	NM_002557	4.39E-04	1.89E+00	8.67E-03
OXER1	NM_148962	7.72E-03	2.42E+00	4.85E-02
OXLD1	NM_001304999	2.18E-03	2.32E+00	2.25E-02
OXR1	NM_181354	7.43E-05	-1.97E+00	3.10E-03
P2RX1	NM_002558	1.13E-04	2.03E+00	4.04E-03
P2RX4	NR_046373	9.66E-04	3.99E+00	1.37E-02
P2RY1	NM_002563	3.62E-04	-2.10E+00	7.84E-03
P2RY2	NM_176071	4.88E-03	1.50E+00	3.65E-02
P3H1	NM_001243246	2.63E-03	1.87E+00	2.52E-02
P4HTM	NM_177938	5.80E-03	1.91E+00	4.05E-02
PABPC1L	NR_134987	6.65E-03	1.76E+00	4.41E-02
PAFAH1B2	NM_002572	5.24E-04	-1.54E+00	9.58E-03
PAFAH1B3	NM_001145940	1.89E-03	2.10E+00	2.07E-02
PALLD	NM_001367569	4.29E-04	-2.01E+00	8.56E-03
PALM2-AKAP2	NM_001136562	1.39E-06	-2.08E+00	4.52E-04
PAM16	NM_016069	1.52E-03	2.09E+00	1.81E-02
PAN3	NM_175854	4.89E-05	-1.62E+00	2.44E-03
PANK3	NM_024594	4.97E-04	-1.55E+00	9.26E-03
PAPOLG	NM_022894	3.05E-06	-1.78E+00	6.11E-04
PAQR3	NR_146462	6.88E-04	-2.80E+00	1.12E-02
PAQR6	NM_198406	2.72E-04	2.68E+00	6.69E-03
PARN	NM_001242992	5.33E-05	2.53E+00	2.58E-03
PARP15	NM_001113523	1.11E-03	-2.54E+00	1.49E-02
PARP6	NR_136611	2.05E-05	1.80E+00	1.54E-03

PARP8	NM_024615	7.98E-04	-1.55E+00	1.22E-02
PAWR	NM_002583	6.18E-03	-1.56E+00	4.21E-02
PAX5	NM_001280551	2.13E-04	-3.08E+00	5.84E-03
PAX8-AS1	NR_047570	3.51E-03	1.93E+00	2.97E-02
PBXIP1	NM_001317735	7.18E-03	1.72E+00	4.64E-02
PCDH9	NM_001318374	5.76E-03	-2.50E+00	4.04E-02
PCF11	NM_001346413	8.63E-05	-1.94E+00	3.42E-03
PCGF5	NM_001257101	3.98E-03	-1.76E+00	3.21E-02
PCM1	NM_001352658	5.49E-07	-2.74E+00	3.42E-04
PCMTD1	NM_052937	7.18E-06	-2.28E+00	8.72E-04
PCNP	NR_135227	3.83E-03	-1.54E+00	3.13E-02
PCNX1	NM_014982	7.07E-03	-1.51E+00	4.58E-02
PCNX4	NM_022495	1.07E-04	-1.69E+00	3.93E-03
PCOLCE	NM_002593	6.11E-03	1.86E+00	4.18E-02
PCYT2	NM_001256435	8.02E-03	2.04E+00	4.97E-02
PDCD2L	NM_032346	3.97E-03	1.53E+00	3.20E-02
PDCD4	NM_145341	3.84E-03	-1.69E+00	3.14E-02
PDCD6IP	NM_001256192	1.39E-04	-2.30E+00	4.52E-03
PDE12	NM_001322177	1.79E-03	-1.63E+00	2.01E-02
PDE1B	NM_001288769	2.18E-04	2.68E+00	5.92E-03
PDE3B	NM_000922	1.34E-03	-1.64E+00	1.68E-02
PDE4B	NM_001037339	3.07E-03	-1.65E+00	2.74E-02
PDE4D	NM_001197221	4.19E-03	-1.93E+00	3.30E-02
PDE6B	NM_001145292	6.41E-03	1.54E+00	4.31E-02
PDGFD	NM_025208	3.72E-05	-3.24E+00	2.13E-03
PDIA4	NR_163906	3.59E-03	6.28E+00	3.01E-02
PDP1	NM_018444	1.05E-03	-1.86E+00	1.44E-02
PDPK1	NM_002613	4.17E-05	-1.83E+00	2.22E-03
PDPR	NM_001322118	1.04E-04	1.54E+00	3.86E-03
PDRG1	NM_030815	8.38E-04	1.57E+00	1.26E-02
PDS5B	NM_015032	2.21E-05	-1.50E+00	1.61E-03
PDXDC1	NM_001285449	1.79E-14	2.73E+01	5.97E-10
PEAK1	NM_024776	2.72E-05	-1.53E+00	1.81E-03
PELI1	NM_020651	2.37E-05	-2.07E+00	1.67E-03
PELI2	NM_021255	8.49E-04	-1.54E+00	1.27E-02
PEX10	NM_002617	3.89E-03	1.58E+00	3.16E-02
PEX6	NM_001316313	3.82E-04	5.73E+00	8.07E-03
PFDN5	NM_002624	5.68E-04	2.48E+00	1.00E-02

PFDN6	NM_001265596	5.80E-03	1.77E+00	4.05E-02
PFKFB4	NM_001317136	3.03E-03	1.61E+00	2.73E-02
PFN1	NM_005022	3.62E-03	1.56E+00	3.03E-02
PGAP1	NM_001321100	9.16E-04	-1.91E+00	1.33E-02
PGAP2	NR_144429	3.71E-05	1.89E+00	2.13E-03
PGGHG	NM_025092	7.44E-03	2.51E+00	4.74E-02
PGP	NM_001042371	2.30E-04	1.79E+00	6.11E-03
PHACTR2	NM_001100166	2.34E-06	-1.77E+00	5.56E-04
PHB	NM_002634	4.07E-03	1.81E+00	3.24E-02
PHB2	NM_001144831	3.21E-05	1.79E+00	1.99E-03
PHC3	NM_024947	3.34E-04	-1.56E+00	7.52E-03
PHETA1	NM_001177997	4.86E-04	2.47E+00	9.19E-03
PHF21A	NM_001352032	6.91E-03	-1.99E+00	4.51E-02
PHF23	NM_001284518	4.01E-03	1.51E+00	3.22E-02
PHF3	NM_001290259	2.65E-06	-3.02E+00	5.85E-04
PHF6	NM_032458	5.17E-04	-2.35E+00	9.50E-03
PHKG2	NM_000294	2.04E-04	2.09E+00	5.70E-03
PHLDB2	NM_145753	1.56E-04	-3.06E+00	4.82E-03
PHLPP2	NM_015020	5.26E-05	-2.10E+00	2.57E-03
PHTF2	NM_020432	1.38E-03	-2.23E+00	1.71E-02
PHYKPL	NM_153373	4.80E-03	1.92E+00	3.60E-02
PI3	NM_002638	4.75E-03	1.91E+00	3.58E-02
PI4K2B	NM_018323	2.56E-03	-1.58E+00	2.48E-02
PI4KAP1	NR_003563	3.68E-04	1.91E+00	7.93E-03
PI4KB	NM_001198775	2.72E-05	3.93E+00	1.81E-03
PIAS2	NM_001324060	1.28E-03	-4.26E+00	1.63E-02
PICALM	NM_001206946	4.74E-03	-1.66E+00	3.58E-02
PIGBOS1	NM_001308423	1.28E-03	2.49E+00	1.63E-02
PIGG	NM_001289051	7.83E-04	-2.67E+00	1.20E-02
PIH1D1	NM_017916	7.34E-03	1.62E+00	4.70E-02
PIK3AP1	NM_152309	1.16E-03	-1.59E+00	1.53E-02
PIK3C2A	NM_002645	1.83E-06	-3.16E+00	4.95E-04
PIK3C3	NM_001308020	2.15E-03	1.75E+00	2.23E-02
PIK3CB	NM_001256045	2.62E-04	2.12E+00	6.59E-03
PIK3R1	NM_181523	4.34E-05	-2.51E+00	2.27E-03
PIK3R6	NM_001010855	6.23E-03	2.01E+00	4.23E-02
PIKFYVE	NM_015040	2.88E-06	-1.72E+00	5.95E-04
PILRB	NM_178238	7.03E-06	1.87E+00	8.64E-04

PIM3	NM_001001852	5.86E-03	1.62E+00	4.07E-02
PIN4	NM_006223	6.86E-04	1.69E+00	1.11E-02
PJA2	NM_014819	2.36E-04	-1.72E+00	6.18E-03
PKN2	NM_006256	4.29E-06	-2.49E+00	7.14E-04
PKP2	NM_001005242	1.57E-04	-2.21E+00	4.84E-03
PLA2G15	NM_001363551	3.26E-03	1.84E+00	2.84E-02
PLA2G4B	NM_001114633	5.50E-03	2.15E+00	3.94E-02
PLAC8	NM_001130715	1.30E-03	1.68E+00	1.64E-02
PLB1	NR_138141	1.56E-05	2.98E+00	1.31E-03
PLCB1	NM_015192	2.58E-03	-2.04E+00	2.50E-02
PLCB2	NM_001284297	3.44E-04	1.61E+00	7.66E-03
PLCL1	NM_006226	3.09E-03	-1.75E+00	2.75E-02
PLD2	NM_002663	5.95E-05	2.03E+00	2.71E-03
PLEKHA2	NM_021623	9.48E-09	-1.55E+00	4.53E-05
PLEKHA5	NM_019012	7.22E-03	-1.91E+00	4.66E-02
PLEKHA8	NM_001197026	5.55E-04	-1.53E+00	9.88E-03
PLEKHF2	NM_024613	9.29E-05	-1.79E+00	3.61E-03
PLEKHG1	NM_001329799	8.33E-04	-2.55E+00	1.25E-02
PLEKHJ1	NM_018049	6.59E-03	2.11E+00	4.39E-02
PLEKHO1	NM_001304723	4.83E-03	1.52E+00	3.62E-02
PLP2	NM_002668	5.47E-04	1.65E+00	9.82E-03
PLXNC1	NM_005761	2.62E-03	-1.94E+00	2.52E-02
PM20D2	NM_001010853	1.17E-04	-1.58E+00	4.10E-03
PMF1	NM_007221	2.39E-03	1.70E+00	2.38E-02
PMM1	NM_002676	3.08E-03	2.00E+00	2.75E-02
PMM2	NM_000303	5.77E-05	1.65E+00	2.67E-03
PMS1	NM_001321047	2.47E-03	-2.20E+00	2.43E-02
PMVK	NM_006556	4.10E-03	2.01E+00	3.26E-02
PNISR	NM_001322419	6.35E-04	-1.60E+00	1.07E-02
PNKD	NM_015488	6.53E-04	1.81E+00	1.09E-02
PNMA3	NM_013364	5.83E-03	2.23E+00	4.06E-02
PNRC1	NM_006813	6.79E-06	-1.66E+00	8.51E-04
POC1B	NM_172240	7.33E-03	-1.54E+00	4.70E-02
POGLUT3	NM_153705	5.58E-04	-2.50E+00	9.91E-03
POGZ	NM_001194937	5.64E-03	-1.62E+00	3.99E-02
POLA1	NM_001330360	1.91E-03	-1.55E+00	2.08E-02
POLDIP3	NM_178136	1.16E-03	1.85E+00	1.53E-02
POLE4	NM_019896	6.77E-04	1.79E+00	1.11E-02

POLI	NM_001351617	1.70E-04	2.65E+00	5.10E-03
POLK	NM_016218	4.13E-03	-1.69E+00	3.27E-02
POLL	NR_033406	3.21E-03	2.40E+00	2.81E-02
POLM	NM_001284330	1.01E-04	1.91E+00	3.80E-03
POLR1B	NM_001282772	4.39E-03	-1.75E+00	3.39E-02
POLR1C	NM_203290	6.28E-06	1.53E+00	8.32E-04
POLR2F	NM_021974	1.50E-03	1.91E+00	1.80E-02
POLR2H	NM_001278698	1.31E-03	1.53E+00	1.65E-02
POLR2J	NM_006234	1.11E-03	1.64E+00	1.49E-02
POLR2J3	NM_001097615	7.79E-04	1.75E+00	1.20E-02
POMT1	NR_148400	4.09E-03	4.21E+00	3.25E-02
POP7	NM_005837	4.07E-04	1.76E+00	8.31E-03
POU6F1	NR_147048	5.43E-04	1.94E+00	9.79E-03
PPARA	NM_005036	7.88E-05	-1.50E+00	3.22E-03
PPARD	NM_001171819	9.52E-04	1.92E+00	1.36E-02
PPARG	NM_005037	7.54E-04	7.25E+00	1.18E-02
PPARGC1B	NM_133263	7.47E-03	-1.50E+00	4.76E-02
PPAT	NM_002703	5.62E-04	-2.12E+00	9.95E-03
PPCDC	NM_021823	3.65E-04	2.06E+00	7.89E-03
PPFIA4	NM_001304331	6.30E-04	1.95E+00	1.06E-02
PPIE	NR_036544	3.92E-04	1.71E+00	8.20E-03
PPIEL	NR_144356	2.31E-03	2.48E+00	2.33E-02
PPM1A	NM_177951	1.33E-04	-1.72E+00	4.43E-03
PPM1B	NM_177968	2.14E-04	-1.65E+00	5.85E-03
PPM1M	NM_001122870	3.01E-03	1.74E+00	2.72E-02
PPM1N	NM_001080401	7.64E-05	1.78E+00	3.15E-03
PPP1CA	NM_001008709	6.89E-04	1.57E+00	1.12E-02
PPP1R12A	NM_001143885	1.82E-03	-1.95E+00	2.03E-02
PPP1R15B	NM_032833	1.23E-04	-1.57E+00	4.22E-03
PPP1R21	NM_152994	1.02E-03	-2.49E+00	1.42E-02
PPP2R1B	NM_181699	4.54E-03	-2.16E+00	3.47E-02
PPP2R5C	NM_001161726	1.36E-04	-1.62E+00	4.48E-03
PPP2R5E	NM_006246	7.06E-06	-2.44E+00	8.64E-04
PPP3CA	NM_001130691	7.60E-04	-1.69E+00	1.19E-02
PPP3CB	NM_001289969	3.39E-04	-2.54E+00	7.58E-03
PPP3R1	NM_000945	2.33E-04	-1.59E+00	6.15E-03
PPP4R2	NM_174907	3.06E-03	-1.71E+00	2.73E-02
PPP4R3A	NM_001284281	5.91E-05	2.21E+00	2.69E-03

PPP4R3B	NM_001122964	7.58E-04	-1.66E+00	1.19E-02
PPP6R3	NM_001352368	3.93E-05	-2.90E+00	2.20E-03
PPT2-EGFL8	NR_037861	2.14E-03	2.68E+00	2.22E-02
PPTC7	NM_139283	7.32E-04	-2.07E+00	1.16E-02
PQBP1	NM_005710	4.06E-04	1.61E+00	8.31E-03
PRADC1	NM_032319	9.57E-04	2.30E+00	1.36E-02
PRAF2	NM_007213	9.97E-04	1.67E+00	1.40E-02
PRDM1	NM_001198	2.19E-05	-2.53E+00	1.61E-03
PRDM10	NM_199437	3.89E-03	-4.04E+00	3.16E-02
PRDM2	NM_001007257	1.81E-06	-1.56E+00	4.95E-04
PRDX1	NM_181696	6.86E-03	1.61E+00	4.50E-02
PRDX4	NM_006406	1.05E-03	1.61E+00	1.44E-02
PREB	NM_001330484	3.61E-05	2.38E+00	2.11E-03
PRELID1	NM_013237	1.97E-04	1.90E+00	5.61E-03
PREPL	NM_006036	1.28E-04	-3.31E+00	4.30E-03
PRH1-PRR4	NR_037918	8.30E-04	2.28E+00	1.25E-02
PRICKLE1	NM_001144882	6.39E-03	-2.05E+00	4.30E-02
PRKAA1	NM_006251	6.35E-06	-1.95E+00	8.32E-04
PRKAR1B	NM_001164759	7.15E-03	2.37E+00	4.62E-02
PRKAR2A	NM_004157	4.03E-04	-1.52E+00	8.31E-03
PRKCE	NM_005400	6.24E-04	-2.03E+00	1.06E-02
PRKDC	NM_001081640	2.09E-03	-1.87E+00	2.19E-02
PRKRA	NM_001316362	7.71E-03	-1.97E+00	4.84E-02
PRKX	NM_005044	8.68E-06	-1.68E+00	9.71E-04
PRMT7	NR_147056	6.23E-03	1.83E+00	4.23E-02
PRMT9	NM_138364	3.46E-04	-1.63E+00	7.68E-03
PRNP	NM_000311	6.85E-04	-1.71E+00	1.11E-02
PRORP	NM_001256681	5.54E-05	1.51E+00	2.61E-03
PROSER1	NM_025138	3.43E-05	-1.74E+00	2.07E-03
PRPF38B	NM_018061	9.07E-05	-1.57E+00	3.56E-03
PRPF40B	NM_001031698	3.54E-03	2.59E+00	2.99E-02
PRPF4B	NR_146784	1.31E-03	-8.72E+00	1.65E-02
PRRC2C	NM_015172	1.28E-06	-1.60E+00	4.37E-04
PRRG4	NM_024081	2.22E-05	-2.18E+00	1.61E-03
PRSS23	NR_120593	1.63E-03	-2.35E+00	1.90E-02
PRTN3	NM_002777	6.61E-03	4.21E+00	4.39E-02
PRXL2B	NM_001195738	4.04E-03	1.98E+00	3.23E-02
PSD	NR_073110	3.35E-03	2.84E+00	2.88E-02

PSMA7	NM_002792	1.95E-03	1.52E+00	2.11E-02
PSMB4	NM_002796	9.08E-05	1.60E+00	3.56E-03
PSMB5	NM_002797	1.51E-03	1.76E+00	1.81E-02
PSMB6	NM_002798	1.24E-03	1.76E+00	1.59E-02
PSMB7	NM_002799	1.37E-05	1.59E+00	1.22E-03
PSMD1	NM_001191037	2.49E-03	-2.00E+00	2.44E-02
PSMD4	NM_002810	6.64E-03	1.50E+00	4.41E-02
PSMD5	NM_001270427	3.73E-03	1.69E+00	3.08E-02
PSMD8	NM_002812	9.29E-04	1.65E+00	1.34E-02
PSMG3	NM_001134340	1.21E-04	2.06E+00	4.19E-03
PSTPIP1	NR_135552	3.31E-03	1.98E+00	2.87E-02
PTAR1	NM_001366936	7.64E-03	-1.77E+00	4.81E-02
PTBP3	NM_001163788	2.09E-06	-2.43E+00	5.21E-04
PTCH1	NM_001083607	7.24E-04	-2.48E+00	1.16E-02
PTDSS1	NM_001290225	4.16E-03	1.63E+00	3.29E-02
PTDSS2	NM_001329544	6.98E-03	1.94E+00	4.55E-02
PTGDR	NM_000953	2.32E-04	-1.90E+00	6.15E-03
PTGER4	NM_000958	2.02E-04	-1.59E+00	5.69E-03
PTK2	NM_001352718	6.53E-04	-2.93E+00	1.09E-02
PTPA	NM_001193397	7.50E-04	1.84E+00	1.18E-02
PTPMT1	NM_001143984	1.63E-04	1.78E+00	4.97E-03
PTPN11	NM_002834	3.87E-07	-1.78E+00	3.14E-04
PTPN12	NM_002835	2.18E-05	-2.32E+00	1.60E-03
PTPN13	NM_006264	1.21E-03	-3.11E+00	1.57E-02
PTPN4	NM_002830	4.55E-03	-1.55E+00	3.48E-02
PTPN6	NM_080548	2.66E-03	1.68E+00	2.54E-02
PTPRC	NR_052021	1.22E-05	-2.40E+00	1.15E-03
PTPRE	NM_001323357	2.43E-03	1.56E+00	2.41E-02
PTPRK	NM_002844	1.74E-04	-3.12E+00	5.18E-03
PTRH2	NM_016077	3.79E-03	1.66E+00	3.11E-02
PTRHD1	NM_001013663	7.38E-04	1.74E+00	1.17E-02
PUF60	NM_001271097	1.60E-03	1.66E+00	1.87E-02
PUM2	NM_001352919	3.91E-05	-1.75E+00	2.19E-03
PWWP2A	NM_001349732	8.33E-04	-2.05E+00	1.25E-02
PXK	NM_001349491	9.00E-04	2.82E+00	1.31E-02
PXMP2	NM_018663	4.32E-04	1.66E+00	8.59E-03
PYCARD	NM_145182	2.57E-03	1.87E+00	2.49E-02
PYGM	NM_005609	5.16E-04	1.96E+00	9.49E-03

PYHIN1	NM_198929	2.06E-03	-2.04E+00	2.17E-02
QKI	NM_206855	3.12E-05	-2.85E+00	1.97E-03
QTRT2	NM_001256835	7.40E-03	-1.69E+00	4.73E-02
R3HCC1L	NM_001256621	6.04E-05	2.98E+00	2.73E-03
R3HDM1	NM_001354200	3.46E-03	-2.42E+00	2.95E-02
R3HDM2	NM_001351204	2.46E-03	-2.68E+00	2.42E-02
RAB11FIP1	NM_001002814	3.33E-03	-1.82E+00	2.87E-02
RAB11FIP2	NM_014904	1.36E-03	-1.62E+00	1.69E-02
RAB27B	NM_004163	1.15E-04	-2.03E+00	4.05E-03
RAB30	NM_014488	1.50E-04	-2.10E+00	4.72E-03
RAB33A	NM_004794	6.55E-04	1.65E+00	1.09E-02
RAB33B	NM_031296	3.56E-03	-1.58E+00	3.00E-02
RAB39A	NM_017516	5.26E-05	-2.18E+00	2.57E-03
RAB3GAP1	NM_001172435	1.24E-04	-3.52E+00	4.23E-03
RAB40C	NM_001172666	1.46E-03	2.25E+00	1.77E-02
RAB5IF	NR_026562	1.52E-03	1.56E+00	1.81E-02
RAB6A	NM_198896	8.93E-04	-1.50E+00	1.31E-02
RAB6C	NM_032144	3.52E-04	-1.84E+00	7.74E-03
RAB6D	NM_001077637	5.49E-04	-1.61E+00	9.83E-03
RABEP1	NM_004703	1.29E-04	-1.60E+00	4.32E-03
RABGAP1L	NM_001243764	6.00E-05	-2.26E+00	2.72E-03
RACK1	NM_006098	5.09E-04	1.71E+00	9.43E-03
RAD17	NM_133338	4.06E-03	-2.10E+00	3.23E-02
RAD21	NM_006265	2.78E-05	-1.64E+00	1.83E-03
RAD9A	NM_001243224	2.11E-03	2.06E+00	2.20E-02
RAF1	NM_001354695	7.59E-04	2.95E+00	1.19E-02
RALGAPA1P1	NR_104269	2.09E-03	-1.82E+00	2.19E-02
RALGAPB	NM_001282918	6.93E-05	-1.56E+00	2.97E-03
RALGPS2	NM_001286247	1.23E-04	-1.88E+00	4.22E-03
RANBP2	NM_006267	3.24E-05	-1.62E+00	1.99E-03
RANBP6	NM_012416	4.61E-03	-2.09E+00	3.51E-02
RANGRF	NM_001177801	1.66E-03	1.60E+00	1.92E-02
RAP1B	NM_001010942	2.89E-03	-1.64E+00	2.66E-02
RAP1GDS1	NM_001100426	2.81E-04	-1.51E+00	6.80E-03
RAP2A	NM_021033	8.83E-04	-1.74E+00	1.30E-02
RAPGEF2	NM_001351724	3.30E-05	-2.33E+00	2.01E-03
RAPGEF6	NM_016340	3.32E-04	-1.73E+00	7.49E-03
RAPH1	NM_213589	1.08E-04	-1.54E+00	3.98E-03

RASA2	NM_006506	4.32E-04	-1.78E+00	8.59E-03
RASAL3	NM_001348028	7.02E-03	1.66E+00	4.56E-02
RASGRP3	NM_001139488	2.06E-04	-2.44E+00	5.72E-03
RB1	NM_000321	8.15E-05	-1.67E+00	3.30E-03
RB1CC1	NM_001083617	2.85E-04	-1.74E+00	6.82E-03
RBBP6	NM_006910	2.67E-04	-1.60E+00	6.65E-03
RBL1	NM_002895	5.87E-03	-1.52E+00	4.07E-02
RBL2	NM_005611	2.04E-03	-1.50E+00	2.16E-02
RBM12B	NM_203390	1.83E-05	-1.68E+00	1.43E-03
RBM26	NM_022118	5.06E-04	-1.54E+00	9.38E-03
RBM27	NM_018989	1.72E-06	-1.58E+00	4.95E-04
RBM34	NR_144490	4.52E-03	-1.79E+00	3.46E-02
RBM41	NM_001324242	5.68E-05	-2.33E+00	2.65E-03
RBM43	NM_198557	6.06E-03	-1.73E+00	4.15E-02
RBM47	NM_001098634	9.65E-04	-2.21E+00	1.37E-02
RBM6	NM_001167582	6.01E-05	1.90E+00	2.72E-03
RBX1	NM_014248	3.34E-03	1.84E+00	2.88E-02
RC3H1	NM_172071	1.40E-03	-1.56E+00	1.72E-02
RC3H2	NM_001354478	1.20E-07	-1.76E+00	1.63E-04
RCAN2	NM_005822	6.55E-03	-2.91E+00	4.38E-02
RCN3	NM_020650	1.03E-03	3.60E+00	1.42E-02
RDX	NM_002906	3.97E-04	-2.12E+00	8.24E-03
RECK	NM_021111	9.88E-05	-1.73E+00	3.76E-03
REELD1	NM_001354631	7.73E-03	2.53E+00	4.85E-02
REL	NM_001291746	1.13E-05	-1.88E+00	1.11E-03
RELCH	NR_144408	2.78E-03	-1.85E+00	2.60E-02
RELT	NM_032871	8.06E-03	1.61E+00	4.99E-02
REPIN1	NM_001362747	1.83E-03	1.61E+00	2.04E-02
RERE	NM_001042681	1.50E-05	-2.18E+00	1.29E-03
RESF1	NM_018169	5.54E-05	-2.13E+00	2.61E-03
REST	NM_005612	9.11E-05	-1.66E+00	3.56E-03
REV3L	NM_001286431	4.45E-04	-1.73E+00	8.73E-03
RFC1	NM_002913	9.97E-05	-1.70E+00	3.78E-03
RFWD3	NM_018124	8.95E-04	-1.92E+00	1.31E-02
RFX3	NM_001282116	3.27E-03	-1.83E+00	2.85E-02
RFX7	NM_001370561	5.06E-03	-1.63E+00	3.74E-02
RGL2	NM_004761	2.60E-04	1.75E+00	6.57E-03
RGL4	NM_001329425	1.09E-03	2.09E+00	1.47E-02

RGPD1	NM_001024457	3.97E-03	1.67E+00	3.20E-02
RGPD5	NM_005054.2	2.43E-03	-1.53E+00	2.40E-02
RGPD8	NM_001164463.1	2.43E-03	-1.53E+00	2.40E-02
RGS3	NM_144489	2.79E-03	2.74E+00	2.61E-02
RHOH	NM_001278360	6.74E-03	2.03E+00	4.45E-02
RHOT2	NM_001352278	3.65E-05	3.15E+00	2.13E-03
RIC1	NM_020829	6.84E-07	-1.89E+00	3.69E-04
RIC3	NR_144485	2.87E-03	1.87E+00	2.64E-02
RICTOR	NM_001285439	1.03E-04	-2.21E+00	3.82E-03
RILPL1	NM_178314	3.74E-03	1.69E+00	3.08E-02
RING1	NM_002931	1.41E-03	1.65E+00	1.73E-02
RIOK3	NM_001348193	3.58E-03	2.23E+00	3.01E-02
RIPK3	NM_006871	1.83E-03	1.57E+00	2.04E-02
RIPOR2	NM_001286445	2.46E-03	-1.65E+00	2.43E-02
RLF	NM_012421	2.08E-04	-1.62E+00	5.75E-03
RMI1	NM_001358291	4.06E-03	-1.72E+00	3.23E-02
RNA28SN1	NR_145822	8.09E-03	1.63E+00	4.99E-02
RNA28SN2	NR_146148.1	8.09E-03	1.63E+00	4.99E-02
RNA28SN5	NR_003287.1	8.09E-03	1.63E+00	4.99E-02
RNASE2	NM_002934	3.38E-03	1.66E+00	2.90E-02
RNASEH2C	NM_032193	2.18E-04	1.88E+00	5.92E-03
RNF111	NM_017610	5.00E-06	-1.81E+00	7.73E-04
RNF112	NM_007148	1.68E-03	1.58E+00	1.93E-02
RNF113A	NM_006978	3.20E-04	1.51E+00	7.32E-03
RNF14	NM_001201365	5.17E-03	-1.51E+00	3.78E-02
RNF145	NM_001199381	1.59E-03	-1.52E+00	1.87E-02
RNF157	NM_052916	9.38E-04	1.92E+00	1.35E-02
RNF167	NM_001370313	1.38E-04	2.18E+00	4.51E-03
RNF181	NM_016494	1.43E-04	1.87E+00	4.59E-03
RNF19A	NM_015435	1.43E-06	-2.78E+00	4.56E-04
RNF215	NM_001017981	2.71E-03	1.78E+00	2.57E-02
RNF25	NM_022453	2.38E-03	1.71E+00	2.37E-02
RNF38	NM_194328	6.81E-05	-1.87E+00	2.94E-03
RNF6	NM_183043	2.12E-03	-1.59E+00	2.21E-02
RNGTT	NM_003800	2.12E-04	-1.93E+00	5.83E-03
RNPC3	NM_017619	1.97E-03	-1.54E+00	2.12E-02
RO60	NM_001173524	1.71E-04	-1.74E+00	5.12E-03
ROCK1	NM_005406	1.20E-04	-1.82E+00	4.18E-03

ROCK1P1	NR_160778	7.80E-03	3.15E+00	4.87E-02
ROCK2	NM_001321643	1.58E-05	-1.59E+00	1.31E-03
ROM1	NM_000327	5.49E-04	2.16E+00	9.83E-03
RP2	NM_006915	4.26E-03	-1.65E+00	3.33E-02
RP9P	NR_003500	6.55E-03	1.61E+00	4.38E-02
RPAIN	NM_001033002	6.62E-03	1.50E+00	4.40E-02
RPE	NM_001318929	4.76E-03	-3.60E+00	3.58E-02
RPGR	NM_000328	5.33E-03	-1.80E+00	3.86E-02
RPL10	NM_001303625	5.02E-06	3.38E+00	7.73E-04
RPL10A	NM_007104	3.61E-04	1.69E+00	7.83E-03
RPL11	NM_000975	1.01E-04	2.42E+00	3.80E-03
RPL12	NM_000976	4.56E-04	1.59E+00	8.87E-03
RPL13	NM_000977	2.74E-04	2.71E+00	6.70E-03
RPL13A	NM_012423	2.27E-04	1.97E+00	6.05E-03
RPL13AP5	NR_026712	7.66E-04	2.40E+00	1.19E-02
RPL14	NM_001034996	3.98E-03	1.91E+00	3.20E-02
RPL15	NM_001253382	1.18E-03	1.58E+00	1.55E-02
RPL17	NM_001369558	1.41E-03	6.24E+00	1.73E-02
RPL18	NM_000979	3.42E-03	1.98E+00	2.92E-02
RPL18A	NM_000980	3.95E-03	2.32E+00	3.19E-02
RPL19	NM_000981	3.99E-03	1.82E+00	3.21E-02
RPL19P12	NR_026660	3.51E-03	1.80E+00	2.97E-02
RPL23	NM_000978	1.29E-03	2.61E+00	1.64E-02
RPL23AP82	NR_026982	3.98E-03	1.53E+00	3.20E-02
RPL23P8	NR_026673	6.67E-03	2.38E+00	4.42E-02
RPL24	NM_000986	1.39E-03	1.63E+00	1.72E-02
RPL27	NM_000988	2.18E-04	2.49E+00	5.92E-03
RPL27A	NM_000990	1.78E-03	1.63E+00	2.00E-02
RPL28	NM_001136137	4.21E-04	1.56E+00	8.45E-03
RPL3	NM_000967	2.62E-03	2.18E+00	2.52E-02
RPL30	NM_000989	7.71E-04	1.67E+00	1.20E-02
RPL31	NM_001099693	6.62E-04	3.40E+00	1.09E-02
RPL32	NM_000994	1.28E-05	2.18E+00	1.18E-03
RPL34	NM_001319236	5.35E-03	2.89E+00	3.86E-02
RPL35	NM_007209	4.63E-03	4.32E+00	3.52E-02
RPL35A	NM_000996	3.95E-03	1.66E+00	3.19E-02
RPL37	NR_159993	4.72E-05	1.90E+00	2.39E-03
RPL37A	NM_000998	1.12E-04	1.81E+00	4.02E-03

RPL4	NM_000968	1.09E-04	1.78E+00	3.98E-03
RPL41	NM_021104	9.14E-04	2.26E+00	1.33E-02
RPL6	NM_000970	5.81E-03	1.60E+00	4.05E-02
RPL7A	NM_000972	1.69E-03	1.85E+00	1.93E-02
RPL8	NM_000973	2.38E-03	2.65E+00	2.37E-02
RPL9	NM_001024921	4.93E-03	2.67E+00	3.67E-02
RPLP0	NM_001002	2.38E-05	2.48E+00	1.67E-03
RPP21	NM_024839	1.36E-03	2.07E+00	1.69E-02
RPP25	NM_017793	1.16E-05	1.78E+00	1.11E-03
RPRD2	NM_015203	1.01E-04	-1.61E+00	3.80E-03
RPS10	NM_001014	3.50E-04	2.43E+00	7.73E-03
RPS10-NUDT3	NM_001202470	1.73E-03	1.99E+00	1.97E-02
RPS11	NM_001015	2.59E-04	2.04E+00	6.55E-03
RPS12	NM_001016	6.35E-03	1.62E+00	4.28E-02
RPS14	NM_005617	1.22E-04	2.14E+00	4.21E-03
RPS14P3	NR_077246	4.25E-04	1.79E+00	8.52E-03
RPS15A	NM_001030009	1.21E-03	2.15E+00	1.57E-02
RPS16	NM_001363860	6.10E-04	2.61E+00	1.04E-02
RPS17	NM_001021	1.87E-03	2.49E+00	2.06E-02
RPS18	NM_022551	3.45E-04	2.22E+00	7.66E-03
RPS18P9	NR_077247	1.05E-03	1.98E+00	1.44E-02
RPS19	NM_001022	5.16E-03	2.48E+00	3.77E-02
RPS2	NM_002952	7.45E-03	2.04E+00	4.75E-02
RPS20	NM_001023	1.01E-03	1.80E+00	1.40E-02
RPS21	NM_001024	1.12E-03	2.77E+00	1.50E-02
RPS25	NM_001028	1.41E-04	1.78E+00	4.54E-03
RPS26P11	NR_002309	5.51E-03	2.96E+00	3.94E-02
RPS27	NM_001349947	9.45E-04	2.43E+00	1.35E-02
RPS27A	NM_002954	2.97E-05	2.02E+00	1.92E-03
RPS29	NM_001032	4.81E-04	2.87E+00	9.14E-03
RPS3	NM_001256802	5.78E-05	1.82E+00	2.67E-03
RPS5	NM_001009	3.00E-03	2.68E+00	2.71E-02
RPS6	NM_001010	7.16E-05	1.99E+00	3.02E-03
RPS6KA3	NM_004586	3.60E-04	-1.68E+00	7.82E-03
RPS6KA4	NM_001006944	4.12E-04	1.63E+00	8.35E-03
RPS6KA5	NM_001322230	1.04E-04	-2.79E+00	3.86E-03
RPS6KB2	NM_003952	5.60E-03	2.09E+00	3.98E-02
RPS6KC1	NM_001349646	5.27E-04	-3.23E+00	9.63E-03

RPS7	NM_001011	4.33E-03	2.60E+00	3.36E-02
RPS8	NM_001012	1.56E-04	2.54E+00	4.82E-03
RPS9	NM_001321706	3.23E-03	4.14E+00	2.82E-02
RPSA	NM_002295	4.49E-05	2.15E+00	2.31E-03
RPSAP58	NM_001355283	3.81E-05	2.26E+00	2.17E-03
RPSAP9	NR_026890	6.61E-03	1.55E+00	4.39E-02
RRM2B	NM_015713	1.69E-03	-1.85E+00	1.93E-02
RRN3	NM_018427.1	2.19E-04	-1.83E+00	5.93E-03
RRP12	NM_001284337	5.71E-04	2.49E+00	1.00E-02
RRP36	NR_138081	1.24E-03	1.56E+00	1.59E-02
RSBN1	NM_018364	1.99E-03	-1.53E+00	2.13E-02
RSBN1L	NM_198467	1.39E-04	-1.51E+00	4.52E-03
RSC1A1	NM_006511	1.51E-03	-2.48E+00	1.81E-02
RSF1	NM_016578	1.23E-05	-1.71E+00	1.15E-03
RSPH3	NM_031924	1.76E-05	-2.35E+00	1.41E-03
RSRP1	NR_135791	2.48E-05	-2.75E+00	1.72E-03
RTEL1	NM_001283010	1.51E-03	1.08E+01	1.80E-02
RTTN	NM_173630	2.12E-03	-1.72E+00	2.21E-02
RUFY3	NM_001037442	2.18E-03	-1.86E+00	2.25E-02
RUNDC3A	NM_006695	1.05E-03	-6.63E+00	1.44E-02
RUNX2	NM_001369405	1.18E-03	-1.64E+00	1.54E-02
RUSC2	NM_001330740	2.17E-04	4.93E+00	5.91E-03
RXRB	NM_001291989	1.52E-04	1.63E+00	4.76E-03
RYBP	NM_012234	1.82E-03	-1.52E+00	2.03E-02
S100A12	NM_005621	2.64E-03	2.49E+00	2.53E-02
S100A4	NM_002961	4.62E-05	1.89E+00	2.35E-03
S100A6	NM_014624	3.53E-05	2.17E+00	2.10E-03
S100A8	NM_001319197	6.20E-03	2.97E+00	4.22E-02
S100A9	NM_002965	1.73E-03	2.39E+00	1.97E-02
S1PR5	NM_001166215	9.94E-05	-2.19E+00	3.77E-03
SACM1L	NM_001319073	7.73E-03	-1.73E+00	4.85E-02
SACS	NM_001278055	6.43E-03	-1.69E+00	4.32E-02
SAMD1	NM_138352	3.89E-03	1.71E+00	3.16E-02
SAMD3	NM_001017373	3.94E-03	-1.97E+00	3.19E-02
SAMD4A	NM_001161577	7.81E-03	-2.14E+00	4.88E-02
SAMD9	NM_017654	2.85E-04	-2.40E+00	6.83E-03
SAMD9L	NM_152703	4.01E-05	-3.79E+00	2.20E-03
SAMHD1	NM_015474	4.36E-04	-2.10E+00	8.63E-03

SAP25	NM_001168682	1.90E-03	1.93E+00	2.08E-02
SAP30L	NM_001131063	1.23E-04	-2.46E+00	4.22E-03
SASS6	NM_194292	2.24E-06	-2.71E+00	5.50E-04
SAT2	NM_133491	6.72E-04	1.63E+00	1.10E-02
SATB1	NM_001195470	7.77E-05	-1.71E+00	3.20E-03
SAV1	NM_021818	1.15E-05	-1.74E+00	1.11E-03
SAYSD1	NM_018322	4.21E-04	1.52E+00	8.45E-03
SCAF11	NM_004719	1.20E-06	-1.65E+00	4.21E-04
SCAF8	NM_001286199	2.99E-05	-1.52E+00	1.92E-03
SCAMP1	NM_004866	1.34E-03	-1.92E+00	1.68E-02
SCARB1	NM_005505	8.21E-04	2.34E+00	1.24E-02
SCARB2	NM_001204255	2.00E-03	3.21E+00	2.13E-02
SCD5	NM_001037582	6.33E-03	-1.85E+00	4.27E-02
SCMH1	NM_001172222	2.66E-03	4.08E+00	2.54E-02
SCN3A	NM_001081676	6.85E-03	-1.59E+00	4.50E-02
SCNM1	NM_001204856	5.55E-03	1.70E+00	3.96E-02
SCO2	NM_001169110	2.89E-03	4.20E+00	2.66E-02
SCRN1	NM_001145513	1.40E-04	-1.79E+00	4.53E-03
SCYL2	NM_001330253	2.29E-04	-1.82E+00	6.10E-03
SDAD1	NM_001288983	2.95E-03	1.75E+00	2.68E-02
SDCBP	NM_001348341	5.58E-03	-2.03E+00	3.97E-02
SDHAF1	NM_001042631	6.74E-04	1.78E+00	1.10E-02
SDHAF4	NM_145267	2.39E-04	1.59E+00	6.24E-03
SEC14L1	NM_001143998	3.74E-04	-1.80E+00	7.96E-03
SEC14L1P1	NR_026952	7.28E-04	-1.74E+00	1.16E-02
SEC23A	NM_006364	1.49E-04	-1.56E+00	4.70E-03
SEC24B	NM_001318085	3.53E-04	-1.65E+00	7.75E-03
SEC31A	NM_001077208	1.33E-03	-1.55E+00	1.67E-02
SECISBP2	NM_001282690	1.54E-04	-1.99E+00	4.80E-03
SECISBP2L	NM_001193489	2.89E-06	-2.71E+00	5.95E-04
SEL1L	NM_005065	1.33E-04	-1.54E+00	4.41E-03
SELENOH	NM_170746	4.89E-03	1.67E+00	3.65E-02
SELENOI	NM_033505	3.95E-03	-1.57E+00	3.19E-02
SELENOW	NM_003009	9.91E-05	2.14E+00	3.76E-03
SEMA5A	NM_003966	3.91E-03	-1.72E+00	3.17E-02
SENP1	NR_051992	4.99E-03	-1.73E+00	3.71E-02
SENP6	NM_001304792	1.37E-06	-1.79E+00	4.50E-04
SENP7	NM_001282802	1.21E-04	-2.19E+00	4.20E-03

SEPT5-GP1BB	NR_037611	4.44E-03	2.99E+00	3.42E-02
SEPTIN11	NM_018243	6.99E-04	-1.63E+00	1.13E-02
SEPTIN2	NM_001349314	6.90E-03	1.70E+00	4.51E-02
SEPTIN5	NM_002688	2.98E-03	1.85E+00	2.70E-02
SEPTIN7	NM_001011553	1.47E-05	-1.57E+00	1.27E-03
SERF2	NM_001199878	4.53E-04	2.00E+00	8.82E-03
SERPINB6	NM_001271824	1.96E-03	1.96E+00	2.11E-02
SERPINF1	NM_001329905	1.12E-03	1.76E+00	1.50E-02
SESN1	NM_014454	6.52E-06	-2.05E+00	8.39E-04
SESN3	NM_144665	3.43E-04	-1.60E+00	7.65E-03
SETBP1	NM_015559	5.18E-05	-2.20E+00	2.54E-03
SETDB1	NM_012432	6.25E-03	-1.65E+00	4.24E-02
SETDB2-PHF11	NM_001320727	2.81E-04	-4.48E+00	6.80E-03
SETMAR	NM_001320678	7.52E-03	2.18E+00	4.77E-02
SETX	NM_001351528	1.10E-07	-1.91E+00	1.62E-04
SF3B1	NM_001308824	1.92E-04	-2.69E+00	5.51E-03
SFMBT2	NM_001029880	7.31E-03	-1.55E+00	4.69E-02
SFPQ	NM_005066	7.98E-07	-1.51E+00	3.70E-04
SFRP5	NM_003015	2.71E-03	5.99E+00	2.57E-02
SFXN5	NM_001330408	3.02E-05	2.51E+00	1.93E-03
SGK3	NM_013257	5.79E-04	-1.53E+00	1.01E-02
SGMS1-AS1	NR_126411	7.39E-06	-1.93E+00	8.73E-04
SGPP1	NM_030791	1.29E-04	-1.90E+00	4.32E-03
SGSH	NR_148201	3.16E-04	1.87E+00	7.28E-03
SGSM2	NM_001098509	5.58E-03	1.90E+00	3.97E-02
SH2D1B	NM_053282	4.88E-04	-2.03E+00	9.19E-03
SH3D21	NM_024676	7.74E-04	1.77E+00	1.20E-02
SH3PXD2A	NM_001365079	1.98E-03	-1.53E+00	2.12E-02
SH3RF3	NM_001099289	1.80E-03	1.75E+00	2.02E-02
SH3TC1	NR_134639	1.14E-03	1.97E+00	1.51E-02
SH3TC2	NM_024577	5.57E-03	-1.60E+00	3.96E-02
SHLD1	NM_152504	8.57E-07	1.73E+00	3.76E-04
SHOC2	NM_001324337	4.73E-04	-2.50E+00	9.05E-03
SHPRH	NM_001370327	2.52E-05	-2.72E+00	1.73E-03
SHTN1	NM_001258299	9.86E-04	-3.21E+00	1.39E-02
SIAH1	NM_003031	1.10E-04	-1.54E+00	4.00E-03
SIDT2	NM_001040455	5.07E-03	1.50E+00	3.74E-02
SIGLEC10	NM_001171158	1.18E-03	1.94E+00	1.54E-02

SIGLEC12	NM_053003	1.78E-03	2.37E+00	2.00E-02
SIGLEC7	NM_016543	7.85E-03	2.33E+00	4.89E-02
SIGMAR1	NM_147157	1.08E-03	3.13E+00	1.46E-02
SIN3B	NM_001297597	6.21E-03	2.03E+00	4.23E-02
SIPA1L1	NM_001354289	3.19E-04	4.03E+00	7.31E-03
SIRPB2	NM_001134836	6.47E-04	1.92E+00	1.08E-02
SIRPG	NM_080816	6.60E-03	1.72E+00	4.39E-02
SIRT1	NM_012238	3.72E-05	-2.50E+00	2.13E-03
SIRT5	NM_001193267	5.47E-03	-1.99E+00	3.92E-02
SKIL	NM_001248008	9.65E-06	-2.30E+00	1.02E-03
SKIV2L	NM_006929	3.71E-03	1.62E+00	3.07E-02
SLAMF6	NM_052931	1.00E-03	-1.71E+00	1.40E-02
SLAMF7	NM_021181	4.83E-03	-1.84E+00	3.62E-02
SLC12A2	NM_001046	5.92E-04	-2.08E+00	1.03E-02
SLC12A6	NM_001042497	7.67E-08	-2.92E+00	1.42E-04
SLC16A5	NM_001271765	2.82E-03	2.48E+00	2.62E-02
SLC18A2	NM_003054	5.14E-03	-1.63E+00	3.77E-02
SLC19A1	NM_001205207	9.87E-04	1.81E+00	1.39E-02
SLC25A19	NM_001126122	1.32E-03	2.62E+00	1.66E-02
SLC25A28	NM_031212	1.14E-04	1.60E+00	4.05E-03
SLC25A29	NM_152333	1.79E-04	5.95E+00	5.26E-03
SLC25A39	NR_159401	5.72E-03	2.33E+00	4.02E-02
SLC25A45	NM_001300820	1.71E-03	5.43E+00	1.95E-02
SLC25A53	NM_001012755	4.93E-05	-1.88E+00	2.46E-03
SLC26A11	NM_001166349	3.90E-03	2.14E+00	3.16E-02
SLC26A2	NM_000112	2.14E-04	-1.70E+00	5.85E-03
SLC26A6	NM_001281733	9.19E-04	3.09E+00	1.33E-02
SLC27A3	NR_145826	2.05E-04	2.92E+00	5.71E-03
SLC29A2	NR_125343	1.30E-03	2.46E+00	1.64E-02
SLC2A13	NM_052885	1.61E-03	-1.62E+00	1.88E-02
SLC2A6	NM_001145099	1.42E-03	1.79E+00	1.74E-02
SLC30A6	NM_001193513	1.60E-04	-2.14E+00	4.91E-03
SLC33A1	NM_001190992	7.31E-03	-1.57E+00	4.69E-02
SLC35A3	NM_012243	2.44E-03	-1.50E+00	2.41E-02
SLC35A5	NM_017945	8.43E-05	-2.24E+00	3.38E-03
SLC35C1	NM_001145265	7.64E-04	1.67E+00	1.19E-02
SLC35E3	NM_001354998	2.67E-03	-1.58E+00	2.54E-02
SLC37A4	NM_001467	4.14E-04	1.59E+00	8.37E-03

SLC38A1	NM_030674	1.62E-04	-2.08E+00	4.95E-03
SLC38A2	NM_018976	7.97E-05	-2.02E+00	3.25E-03
SLC38A9	NM_001349382	6.23E-03	-2.73E+00	4.23E-02
SLC39A1	NM_001271959	2.85E-03	2.76E+00	2.63E-02
SLC39A13	NM_152264	5.68E-03	2.96E+00	4.01E-02
SLC39A9	NM_001252150	6.91E-04	1.74E+00	1.12E-02
SLC3A2	NR_037193	2.78E-04	2.04E+00	6.77E-03
SLC41A3	NM_001164475	4.18E-03	1.81E+00	3.30E-02
SLC45A4	NM_001080431	1.13E-03	4.47E+00	1.51E-02
SLC48A1	NM_017842	3.31E-03	1.55E+00	2.87E-02
SLC4A10	NM_001354446	2.48E-04	-4.73E+00	6.39E-03
SLC4A2	NM_001199693	7.71E-03	1.73E+00	4.84E-02
SLC4A4	NM_001098484	3.56E-03	-2.55E+00	3.00E-02
SLC4A7	NM_001321108	4.58E-05	-2.72E+00	2.34E-03
SLC50A1	NM_001287588	4.60E-04	1.57E+00	8.92E-03
SLC5A3	NM_006933	1.55E-03	-1.62E+00	1.83E-02
SLC5A6	NR_028323	2.28E-03	1.68E+00	2.32E-02
SLC8A1	NM_001351494	4.29E-03	-2.27E+00	3.34E-02
SLC9A1	NR_046474	3.35E-04	1.85E+00	7.52E-03
SLC9A3-AS1	NR_125375	6.41E-04	4.37E+00	1.08E-02
SLC9A6	NM_001042537	3.81E-04	-1.72E+00	8.05E-03
SLC9A7	NM_001257291	1.74E-05	-1.77E+00	1.39E-03
SLFN11	NM_001104587	7.32E-06	-2.37E+00	8.73E-04
SLFN5	NM_144975	1.39E-04	-2.14E+00	4.52E-03
SLIRP	NM_001267864	3.13E-04	2.54E+00	7.24E-03
SLIT1	NM_003061	2.95E-03	1.87E+00	2.68E-02
SLK	NM_001304743	4.14E-05	-1.59E+00	2.22E-03
SLMAP	NM_001304421	1.60E-04	-2.67E+00	4.92E-03
SLPI	NM_003064	4.26E-03	2.62E+00	3.33E-02
SLTM	NR_135042	7.82E-04	2.18E+00	1.20E-02
SLX1A-SULT1A3	NR_037608	5.51E-03	1.80E+00	3.94E-02
SLX1B-SULT1A4	NR_037609	5.51E-03	1.80E+00	3.94E-02
SMAD5	NM_001001419	5.51E-03	-1.50E+00	3.94E-02
SMARCA2	NM_003070	2.22E-04	-1.55E+00	5.95E-03
SMARCAD1	NM_020159	1.03E-03	-2.55E+00	1.43E-02
SMARCD3	NM_001003802	1.10E-03	1.86E+00	1.48E-02
SMC2	NM_001042551	4.70E-03	-1.71E+00	3.55E-02

SMC3	NM_005445	1.14E-04	-1.55E+00	4.05E-03
SMC4	NM_001002800	3.57E-05	-1.95E+00	2.10E-03
SMC6	NM_024624	1.22E-03	-1.63E+00	1.58E-02
SMCHD1	NM_015295	6.93E-04	-1.70E+00	1.12E-02
SMCO4	NM_020179	2.16E-03	1.52E+00	2.24E-02
SMDT1	NM_033318	2.00E-03	1.61E+00	2.13E-02
SMG1	NM_015092	4.19E-05	-1.55E+00	2.22E-03
SMG1P3	NR_027155	2.38E-03	-1.66E+00	2.37E-02
SMG1P5	NR_002453	4.64E-03	-1.71E+00	3.53E-02
SMIM29	NM_001008704	5.21E-03	2.85E+00	3.80E-02
SMIM4	NM_001124767	2.08E-04	1.55E+00	5.75E-03
SMN1	NM_022874.1	3.66E-04	-3.30E+00	7.90E-03
SMN2	NM_022876.1	3.84E-04	-3.63E+00	8.08E-03
SMPD1	NR_134502	7.60E-03	2.61E+00	4.80E-02
SMPD2	NM_003080	3.47E-03	1.63E+00	2.95E-02
SMUG1	NM_001243787	1.91E-03	2.36E+00	2.09E-02
SMYD5	NM_006062	1.40E-03	1.66E+00	1.72E-02
SNAI3-AS1	NR_024402	2.66E-04	1.82E+00	6.63E-03
SNAPC3	NR_161433	4.03E-03	1.86E+00	3.23E-02
SNF8	NM_007241	5.94E-04	1.62E+00	1.03E-02
SNHG11	NR_003239	9.35E-05	2.24E+00	3.62E-03
SNHG12	NR_146381	5.76E-03	1.96E+00	4.04E-02
SNHG15	NR_003697	6.46E-04	2.22E+00	1.08E-02
SNHG29	NR_027166	2.81E-03	2.08E+00	2.62E-02
SNHG3	NR_036473	4.04E-03	2.41E+00	3.23E-02
SNHG30	NR_135479	1.66E-03	1.53E+00	1.92E-02
SNHG32	NR_160946	1.38E-04	2.88E+00	4.51E-03
SNHG7	NR_003672	6.09E-04	1.63E+00	1.04E-02
SNHG8	NR_003584	7.86E-03	1.70E+00	4.90E-02
SNORD3A	NR_006880	1.75E-04	3.53E+00	5.21E-03
SNRK	NM_017719	2.66E-06	-2.83E+00	5.85E-04
SNRNP25	NM_024571	3.49E-05	1.93E+00	2.09E-03
SNRPA	NM_004596	4.75E-03	1.54E+00	3.58E-02
SNRPD2	NM_004597	9.00E-05	2.68E+00	3.54E-03
SNRPD3	NM_001278656	2.53E-03	1.79E+00	2.47E-02
SNU13	NM_005008	5.22E-03	1.61E+00	3.80E-02
SNX10	NM_001362754	7.09E-04	-2.74E+00	1.14E-02
SNX13	NM_001350862	7.84E-04	-1.71E+00	1.20E-02

SNX25	NM_001317781	1.61E-04	-1.78E+00	4.92E-03
SNX27	NM_030918	1.86E-03	-1.97E+00	2.06E-02
SNX29	NM_032167	2.30E-05	-1.60E+00	1.64E-03
SNX29P1	NR_045011	2.38E-03	-2.62E+00	2.37E-02
SOCS4	NM_199421	2.86E-03	-1.59E+00	2.64E-02
SOCS5	NM_014011	6.57E-04	-1.82E+00	1.09E-02
SOD2	NM_001024466	3.09E-03	-2.23E+00	2.75E-02
SON	NM_138927	2.94E-04	-4.72E+00	6.96E-03
SORL1	NM_003105	3.58E-03	-1.58E+00	3.01E-02
SOS1	NM_005633	7.42E-07	-1.67E+00	3.69E-04
SOS2	NM_006939	5.54E-03	-1.58E+00	3.95E-02
SP110	NM_004509	1.33E-03	-1.51E+00	1.67E-02
SP140L	NM_001308163	4.10E-04	2.14E+00	8.33E-03
SP3	NM_001172712	3.73E-03	-1.52E+00	3.08E-02
SP4	NM_003112	6.03E-05	-2.40E+00	2.73E-03
SPACA6	NR_024330	7.83E-04	2.22E+00	1.20E-02
SPAG1	NM_172218	2.56E-05	-1.59E+00	1.74E-03
SPAG7	NM_004890	1.03E-03	1.81E+00	1.43E-02
SPAG9	NM_001130528	6.40E-05	-1.67E+00	2.81E-03
SPART	NM_015087	3.25E-03	-1.55E+00	2.83E-02
SPAST	NM_014946	8.66E-04	-1.56E+00	1.28E-02
SPATA13	NM_001286795	1.74E-04	-1.94E+00	5.18E-03
SPATC1L	NM_032261	1.16E-03	3.60E+00	1.53E-02
SPECC1L	NM_001145468	9.13E-04	-1.70E+00	1.32E-02
SPECC1L-ADORA2A	NR_103546	4.92E-03	-4.04E+00	3.67E-02
SPG11	NM_001160227	4.33E-04	-4.85E+00	8.60E-03
SPHK1	NM_001142602	2.83E-03	2.22E+00	2.62E-02
SPIN1	NM_006717	1.88E-05	-1.64E+00	1.46E-03
SPIN4	NM_001012968	1.73E-03	-1.68E+00	1.97E-02
SPINT1	NM_003710	1.73E-03	1.83E+00	1.97E-02
SPINT2	NM_021102	8.13E-04	1.55E+00	1.23E-02
SPIRE1	NM_001128627	1.02E-03	-1.65E+00	1.41E-02
SPOPL	NM_001001664	4.05E-03	-1.55E+00	3.23E-02
SPRTN	NM_001010984	5.69E-04	-1.70E+00	1.00E-02
SPTBN1	NM_178313	1.69E-04	-2.81E+00	5.08E-03
SPTY2D1	NM_194285	2.06E-08	-1.62E+00	6.90E-05
SRA1	NM_001035235	3.16E-04	1.95E+00	7.28E-03
SREBF1	NM_001321096	2.90E-03	1.91E+00	2.66E-02

SREK1	NM_001077199	1.25E-04	-1.67E+00	4.23E-03
SRFBP1	NM_152546	2.72E-04	-1.52E+00	6.69E-03
SRGAP2D	NR_120535	2.22E-03	-2.05E+00	2.27E-02
SRI	NM_001256891	3.34E-03	1.57E+00	2.88E-02
SRPK2	NM_001350741	1.64E-04	-1.66E+00	4.99E-03
SRRM1	NM_001366573	2.01E-03	-1.86E+00	2.14E-02
SRRT	NM_001128853	5.33E-03	1.57E+00	3.86E-02
SRSF10	NR_034035	4.42E-03	-1.78E+00	3.41E-02
SRSF3	NM_003017	1.16E-06	1.64E+00	4.17E-04
SSH2	NM_001282131	4.63E-03	-1.70E+00	3.52E-02
SSPN	NM_001135823	7.08E-04	-1.75E+00	1.14E-02
SSR2	NM_003145	9.73E-06	1.85E+00	1.03E-03
SSR4	NR_037927	5.13E-03	2.21E+00	3.76E-02
SSTR3	NM_001278687	6.35E-03	1.79E+00	4.28E-02
SSX2IP	NM_001166293	1.91E-03	-2.17E+00	2.09E-02
ST3GAL4	NM_001254759	7.56E-03	1.83E+00	4.79E-02
ST6GALNAC6	NR_104629	1.11E-03	2.60E+00	1.48E-02
ST8SIA4	NM_005668	2.86E-04	-1.79E+00	6.84E-03
ST8SIA6	NM_001004470	6.36E-06	-3.53E+00	8.32E-04
STAB1	NM_015136	3.85E-03	2.74E+00	3.14E-02
STAG2	NM_006603	3.21E-05	-1.77E+00	1.99E-03
STAG3L5P	NR_103720	2.88E-04	1.67E+00	6.87E-03
STAM	NM_001324287	1.16E-03	2.26E+00	1.53E-02
STARD4	NM_139164	1.62E-04	-2.93E+00	4.95E-03
STAT1	NM_007315	7.41E-03	-2.07E+00	4.74E-02
STAT3	NM_213662	1.28E-03	-1.58E+00	1.63E-02
STAT6	NM_001178079	4.37E-04	1.52E+00	8.65E-03
STAU1	NM_001322933	9.33E-05	-2.22E+00	3.62E-03
STEAP4	NM_001205316	2.57E-03	2.03E+00	2.49E-02
STK16	NM_001008910	6.55E-03	1.66E+00	4.38E-02
STK26	NM_016542	4.41E-05	-2.29E+00	2.29E-03
STK32C	NM_001318881	2.06E-05	1.65E+00	1.55E-03
STK39	NM_013233	1.74E-05	-1.66E+00	1.39E-03
STK4	NM_006282	1.20E-06	-1.53E+00	4.21E-04
STOM	NM_198194	1.52E-03	2.25E+00	1.81E-02
STON2	NM_001366850	1.34E-03	-1.70E+00	1.67E-02
STRADA	NR_156741	8.11E-04	2.05E+00	1.23E-02
STRBP	NM_018387	4.53E-03	-1.66E+00	3.47E-02

STRN3	NM_001083893	3.73E-03	-2.31E+00	3.08E-02
STT3A	NM_001278504	2.98E-03	1.65E+00	2.70E-02
STX10	NM_003765	3.24E-03	2.19E+00	2.83E-02
STX2	NM_001351052	2.96E-04	3.18E+00	6.98E-03
STX4	NM_001272095	5.96E-03	1.79E+00	4.12E-02
STYXL1	NM_001317787	3.81E-04	2.85E+00	8.05E-03
SUCO	NM_014283	4.55E-04	-2.10E+00	8.85E-03
SULT1A1	NM_001055	7.64E-05	2.16E+00	3.15E-03
SULT1A3	NM_177552	5.99E-04	1.65E+00	1.03E-02
SULT1A4	NM_001017390	5.99E-04	1.65E+00	1.03E-02
SUMF2	NM_001366648	2.32E-03	1.83E+00	2.33E-02
SUPT20H	NM_001278481	6.79E-04	-1.65E+00	1.11E-02
SUPT5H	NM_001319991	4.23E-04	1.96E+00	8.48E-03
SUPV3L1	NM_001323586	1.53E-03	1.75E+00	1.81E-02
SURF1	NM_001280787	5.75E-04	1.52E+00	1.01E-02
SURF2	NM_017503	2.42E-03	1.88E+00	2.40E-02
SUZ12	NM_015355	4.09E-04	-1.74E+00	8.33E-03
SWAP70	NM_015055	4.31E-05	-1.92E+00	2.27E-03
SYNCRIP	NM_001159677	9.49E-04	-1.59E+00	1.36E-02
SYNE1	NM_033071	2.51E-06	-2.85E+00	5.67E-04
SYNE2	NM_015180	7.06E-06	-2.30E+00	8.64E-04
SYNJ1	NM_001160306	3.23E-05	-1.70E+00	1.99E-03
SYNRG	NM_007247	5.10E-04	-1.65E+00	9.43E-03
SYP	NM_003179	6.53E-04	1.98E+00	1.09E-02
SYTL2	NM_206927	3.39E-03	-2.05E+00	2.91E-02
SYTL3	NM_001009991	7.36E-03	1.84E+00	4.71E-02
TAB2	NM_001292034	2.41E-05	-1.92E+00	1.68E-03
TAB3	NM_152787	2.00E-04	-1.71E+00	5.66E-03
TACC1	NM_001352793	2.66E-05	-1.81E+00	1.79E-03
TAF1	NM_001286074	1.05E-04	-2.07E+00	3.89E-03
TAF10	NM_006284	5.91E-06	1.80E+00	8.24E-04
TAF1A	NM_001201536	7.73E-03	-1.72E+00	4.85E-02
TAF1C	NM_139353	5.74E-03	2.46E+00	4.03E-02
TAF1L	NM_153809	2.96E-06	-2.33E+00	6.00E-04
TAF3	NM_031923	1.41E-04	-1.57E+00	4.55E-03
TAFA2	NM_178539	7.95E-03	-2.37E+00	4.94E-02
TAGAP	NM_054114	3.66E-06	-1.59E+00	6.90E-04
TAGLN	NM_001001522	5.43E-04	2.45E+00	9.79E-03

TANGO2	NM_001322166	7.85E-04	2.24E+00	1.21E-02
TANK	NM_133484	2.21E-03	-1.95E+00	2.27E-02
TAOK1	NM_020791	1.09E-04	-1.82E+00	3.98E-03
TAOK3	NM_001346487	1.47E-04	-1.92E+00	4.66E-03
TAPBPL	NR_147129	7.66E-03	1.88E+00	4.82E-02
TARBP2	NM_134324	4.88E-04	2.06E+00	9.19E-03
TARS	NR_047678	4.65E-03	2.57E+00	3.53E-02
TASOR	NM_001112736	2.33E-04	-1.57E+00	6.17E-03
TASOR2	NM_001321783	6.56E-04	-1.92E+00	1.09E-02
TAZ	NM_181313	6.12E-04	2.84E+00	1.05E-02
TBC1D1	NM_001253914	6.94E-07	4.78E+00	3.69E-04
TBC1D10C	NM_001369492	6.21E-06	3.05E+00	8.32E-04
TBC1D14	NM_001113361	1.62E-03	-2.00E+00	1.89E-02
TBC1D2	NM_001267572	9.70E-05	2.53E+00	3.72E-03
TBC1D31	NM_145647	5.65E-06	-3.54E+00	8.21E-04
TBC1D5	NM_001349074	7.19E-06	-1.74E+00	8.72E-04
TBC1D7	NM_001143965	3.75E-03	2.79E+00	3.09E-02
TBCB	NM_001281	1.72E-03	1.97E+00	1.96E-02
TBCEL	NM_001363644	5.20E-04	-1.99E+00	9.53E-03
TBCK	NM_001290768	7.38E-04	-2.30E+00	1.17E-02
TBL1XR1	NM_001321194	1.43E-03	-1.61E+00	1.74E-02
TBXAS1	NM_001366537	8.88E-04	2.88E+00	1.31E-02
TCAF2	NM_001363538.1	6.58E-03	-1.64E+00	4.38E-02
TCEA1	NM_201437	1.18E-03	-1.61E+00	1.54E-02
TCEA3	NM_003196	1.91E-03	1.87E+00	2.08E-02
TCF12	NM_001322151	2.41E-03	-2.84E+00	2.39E-02
TCF4	NM_001348214	1.08E-03	-2.49E+00	1.47E-02
TCIRG1	NM_006053	7.16E-03	2.45E+00	4.63E-02
TCP11L2	NM_152772	1.22E-03	-1.84E+00	1.58E-02
TDG	NM_003211	8.38E-04	-1.71E+00	1.26E-02
TEK	NM_000459	1.26E-03	-8.86E+00	1.61E-02
TEKT4P2	NR_038327	1.18E-03	3.18E+00	1.55E-02
TENT4B	NM_001365324	1.26E-04	-2.00E+00	4.26E-03
TESK2	NM_001320800	1.79E-04	3.04E+00	5.26E-03
TET2	NM_017628	2.46E-04	-2.40E+00	6.36E-03
TET3	NM_001366022	5.88E-04	-1.55E+00	1.02E-02
TFCP2L1	NM_014553	9.32E-04	-2.42E+00	1.34E-02
TFDP2	NM_001178140	2.52E-03	-1.70E+00	2.46E-02

TFRC	NM_001128148	1.37E-05	-2.33E+00	1.22E-03
TGFBR1	NM_004612	2.13E-03	-1.57E+00	2.22E-02
TGFBR3	NM_001195683	4.13E-04	-2.03E+00	8.35E-03
TGM1	NM_000359	4.21E-03	1.79E+00	3.31E-02
TGM3	NM_003245	1.36E-03	2.42E+00	1.70E-02
THAP5	NM_001130475	6.31E-05	-1.93E+00	2.80E-03
THAP6	NR_133920	6.58E-04	1.54E+00	1.09E-02
THBS3	NM_001252608	3.84E-04	2.23E+00	8.08E-03
THOC2	NM_001081550	1.03E-05	-1.63E+00	1.05E-03
THOC5	NM_001002879	7.08E-03	1.73E+00	4.59E-02
THUMPD1	NM_017736	3.93E-04	-2.12E+00	8.20E-03
THYN1	NM_001037305	7.72E-04	1.68E+00	1.20E-02
TIA1	NM_001351516	6.48E-05	-3.44E+00	2.83E-03
TIAF1	NM_004740	4.03E-06	2.05E+00	7.03E-04
TIAM1	NM_001353688	9.36E-05	-3.36E+00	3.63E-03
TIMM17B	NM_001167947	7.50E-04	1.81E+00	1.18E-02
TIMM50	NM_001001563	4.40E-03	1.54E+00	3.40E-02
TIMM8B	NM_012459	9.44E-04	2.88E+00	1.35E-02
TIMP1	NM_003254	7.23E-03	1.80E+00	4.66E-02
TLE1	NM_001303103	6.86E-05	-3.49E+00	2.96E-03
TLE4	NM_001351542	2.02E-03	-2.21E+00	2.15E-02
TLK2	NM_001330418	6.27E-03	-2.02E+00	4.25E-02
TLR4	NM_003266	1.04E-03	-2.06E+00	1.43E-02
TM2D2	NM_078473	1.79E-05	2.16E+00	1.41E-03
TMC8	NM_152468	4.89E-03	1.92E+00	3.65E-02
TMCC1	NM_001349263	2.84E-04	-2.25E+00	6.82E-03
TMED3	NM_007364	4.17E-04	1.86E+00	8.40E-03
TMED9	NM_017510	1.66E-03	1.56E+00	1.92E-02
TMEM101	NM_032376	3.70E-04	1.81E+00	7.93E-03
TMEM104	NM_001321264	1.50E-03	3.07E+00	1.80E-02
TMEM106C	NM_001143841	4.20E-03	1.53E+00	3.30E-02
TMEM120A	NM_031925	2.50E-03	1.85E+00	2.45E-02
TMEM129	NM_138385	2.09E-06	2.83E+00	5.21E-04
TMEM141	NM_032928	2.04E-04	2.78E+00	5.71E-03
TMEM143	NR_130317	3.85E-03	2.42E+00	3.14E-02
TMEM147	NM_032635	1.01E-03	1.68E+00	1.41E-02
TMEM147-AS1	NR_038396	3.70E-04	1.99E+00	7.93E-03
TMEM14B	NM_030969	4.67E-03	1.89E+00	3.54E-02

TMEM168	NM_022484	1.91E-04	-1.79E+00	5.49E-03
TMEM175	NM_001297428	2.88E-03	2.98E+00	2.65E-02
TMEM179B	NM_199337	1.13E-03	1.55E+00	1.51E-02
TMEM183A	NR_136530	4.01E-04	2.08E+00	8.28E-03
TMEM185A	NR_104121	2.83E-04	5.05E+00	6.80E-03
TMEM187	NM_003492	2.82E-03	1.70E+00	2.62E-02
TMEM205	NM_198536	4.78E-05	1.81E+00	2.41E-03
TMEM208	NR_134524	3.16E-03	2.58E+00	2.79E-02
TMEM214	NM_001083590	1.44E-03	2.06E+00	1.75E-02
TMEM219	NM_194280	6.57E-03	1.78E+00	4.38E-02
TMEM223	NM_001080501	8.35E-06	1.79E+00	9.49E-04
TMEM234	NM_019118	1.20E-03	1.66E+00	1.57E-02
TMEM241	NR_148382	1.43E-03	2.35E+00	1.74E-02
TMEM255A	NM_001104544	8.58E-06	-4.86E+00	9.62E-04
TMEM256	NM_152766	2.46E-05	2.92E+00	1.71E-03
TMEM258	NM_014206	1.01E-04	1.57E+00	3.80E-03
TMEM30A	NM_018247	9.05E-05	-1.75E+00	3.56E-03
TMEM38A	NM_024074	4.22E-03	1.54E+00	3.31E-02
TMEM42	NM_144638	6.10E-04	1.62E+00	1.04E-02
TMEM64	NM_001008495	1.01E-06	-2.12E+00	3.95E-04
TMEM79	NR_026678	1.50E-03	1.61E+00	1.80E-02
TMEM80	NM_001042463	2.51E-03	1.72E+00	2.45E-02
TMEM94	NM_001351203	5.53E-03	1.80E+00	3.95E-02
TMEM99	NR_160888	8.92E-04	1.57E+00	1.31E-02
TMF1	NM_001363879	1.78E-04	-1.63E+00	5.26E-03
TMSB10	NM_021103	3.71E-04	1.75E+00	7.93E-03
TMTC3	NR_159381	1.78E-05	-3.71E+00	1.41E-03
TMUB2	NM_001353178	1.28E-03	2.74E+00	1.63E-02
TMX3	NM_019022	2.43E-03	-1.95E+00	2.40E-02
TNFAIP3	NM_001270507	2.15E-05	-1.55E+00	1.59E-03
TNFAIP8L2-SCNM1	NM_001204848	2.23E-03	2.37E+00	2.28E-02
TNFRSF10D	NM_003840	1.01E-05	1.57E+00	1.05E-03
TNFRSF12A	NM_016639	3.31E-03	2.49E+00	2.86E-02
TNFRSF25	NM_148967	2.66E-03	3.13E+00	2.54E-02
TNFSF4	NM_001297562	7.08E-05	-2.15E+00	3.00E-03
TNIK	NM_001161562	5.53E-04	-1.82E+00	9.87E-03
TNIP2	NM_001161527	7.76E-03	2.01E+00	4.86E-02
TNK1	NM_003985	2.79E-03	2.03E+00	2.61E-02

TNKS2	NM_025235	3.31E-06	-1.63E+00	6.35E-04
TNNI2	NM_001145841	7.67E-03	2.40E+00	4.82E-02
TNNT1	NM_001126133	1.49E-04	4.53E+00	4.70E-03
TNNT3	NM_001367852	4.13E-04	2.21E+00	8.35E-03
TNPO2	NM_013433	7.45E-04	1.55E+00	1.18E-02
TNRC6B	NM_001162501	1.85E-04	-1.55E+00	5.38E-03
TOB1	NM_005749	3.18E-06	-2.22E+00	6.24E-04
TOGARAM1	NM_001308120	5.71E-03	-1.82E+00	4.02E-02
TOMM6	NM_001134493	7.31E-05	1.69E+00	3.07E-03
TOMM7	NM_019059	6.84E-04	2.28E+00	1.11E-02
TOP3A	NM_001320759	5.71E-03	1.63E+00	4.02E-02
TOPBP1	NM_007027	4.42E-05	-1.58E+00	2.30E-03
TOPORS	NM_005802	4.73E-04	-1.79E+00	9.05E-03
TOR1AIP1	NM_001267578	3.96E-04	-1.56E+00	8.24E-03
TOR2A	NM_001134431	1.62E-05	1.92E+00	1.33E-03
TOX	NM_014729	7.56E-07	-2.43E+00	3.69E-04
TP53BP1	NM_001141979	9.94E-04	-1.87E+00	1.40E-02
TP53BP2	NM_005426	7.85E-04	-1.69E+00	1.20E-02
TP53I11	NM_001318387	1.37E-03	2.38E+00	1.70E-02
TP53INP1	NM_001135733	2.82E-04	-1.81E+00	6.80E-03
TP53TG1	NR_015381	5.52E-04	1.75E+00	9.86E-03
TPCN1	NM_001301214	1.68E-05	2.40E+00	1.36E-03
TPD52L2	NM_001243892	1.46E-03	1.99E+00	1.77E-02
TPGS2	NM_001271951	1.04E-03	1.55E+00	1.43E-02
TPI1	NM_001258026	2.45E-06	2.18E+00	5.67E-04
TPM2	NM_001301226	3.48E-04	2.32E+00	7.70E-03
TPM3	NM_001364683	1.77E-04	-1.50E+00	5.23E-03
TPMT	NM_001346818	3.03E-04	2.73E+00	7.11E-03
TPPP	NM_007030	4.70E-03	-1.69E+00	3.56E-02
TPRA1	NR_073377	4.60E-03	1.99E+00	3.50E-02
TPT1-AS1	NR_024458	2.35E-07	2.06E+00	2.38E-04
TRA2A	NM_001362761	1.27E-03	-1.67E+00	1.62E-02
TRABD2A	NM_001277053	2.78E-03	2.05E+00	2.60E-02
TRAF3IP3	NR_109871	2.64E-03	-7.61E+00	2.53E-02
TRAIP	NM_005879	1.89E-03	1.65E+00	2.07E-02
TRAK1	NM_001265608	4.27E-03	-1.64E+00	3.33E-02
TRANK1	NM_001329998	1.04E-03	-1.63E+00	1.43E-02
TRAPPC1	NM_001166621	5.16E-03	1.58E+00	3.77E-02

TRAPPC10	NM_003274	5.79E-06	-1.74E+00	8.21E-04
TRAPPC2L	NM_001318525	4.41E-04	1.99E+00	8.67E-03
TRAPPC3	NM_001270894	1.17E-03	2.10E+00	1.54E-02
TRAPPC6A	NM_001270893	3.73E-03	3.47E+00	3.08E-02
TRAPPC8	NM_014939	4.85E-04	-1.59E+00	9.18E-03
TREML3P	NR_027256	1.45E-03	2.52E+00	1.76E-02
TRIM23	NM_001656	1.82E-03	-1.64E+00	2.03E-02
TRIM52-AS1	NR_102761	5.24E-03	2.02E+00	3.81E-02
TRIM59	NM_173084	6.23E-03	-1.52E+00	4.23E-02
TRIM59-IFT80	NR_148402	3.19E-03	-3.07E+00	2.81E-02
TRIP12	NM_001348323	1.53E-06	-2.42E+00	4.73E-04
TRIR	NM_024038	4.56E-05	1.96E+00	2.34E-03
TRIT1	NM_001312691	6.82E-03	-4.94E+00	4.48E-02
TRMT1	NM_001351761	3.21E-03	3.37E+00	2.82E-02
TRMT112	NM_016404	6.77E-04	1.64E+00	1.11E-02
TRNT1	NR_159940	7.87E-03	-1.69E+00	4.91E-02
TRPM7	NM_001301212	1.10E-03	-1.91E+00	1.48E-02
TRPS1	NM_014112	3.74E-04	-2.66E+00	7.97E-03
TSC2	NM_001318831	6.76E-03	3.90E+00	4.45E-02
TSC22D1	NM_183422	3.07E-04	-1.71E+00	7.18E-03
TSC22D2	NM_001303264	2.50E-06	-1.81E+00	5.67E-04
TSHZ1	NM_005786	7.93E-04	-1.82E+00	1.21E-02
TSNAX-DISC1	NR_028393	7.40E-03	-1.87E+00	4.73E-02
TSPAN13	NM_014399	5.04E-04	-1.81E+00	9.36E-03
TSPEAR	NM_144991	3.69E-04	2.64E+00	7.93E-03
TSPYL5	NM_033512	3.16E-04	-1.77E+00	7.28E-03
TSTA3	NM_003313	3.68E-03	2.40E+00	3.05E-02
TSTD1	NM_001113205	5.09E-04	1.83E+00	9.43E-03
TTC3	NM_001330683	8.74E-06	-3.72E+00	9.74E-04
TTF1	NR_134525	2.65E-04	-1.70E+00	6.62E-03
TTLL3	NR_037162	1.71E-03	1.66E+00	1.95E-02
TTN	NM_133437	1.43E-03	-1.90E+00	1.74E-02
TTPAL	NM_001039199	3.59E-04	-1.57E+00	7.81E-03
TUBB	NM_001293216	6.36E-04	2.03E+00	1.07E-02
TUBBP5	NR_027156	1.47E-04	2.35E+00	4.67E-03
TUG1	NR_152869	2.52E-05	-2.34E+00	1.73E-03
TUT4	NM_015269	7.06E-05	-1.66E+00	3.00E-03
TUT7	NM_024617	1.26E-05	-2.13E+00	1.17E-03

TWNK	NM_001163814	5.46E-03	1.98E+00	3.92E-02
TXK	NM_003328	3.96E-04	-2.04E+00	8.24E-03
TXN2	NM_012473	1.01E-04	1.93E+00	3.80E-03
TXNDC11	NM_001324022	3.97E-04	1.91E+00	8.24E-03
TXNIP	NM_006472	2.62E-04	-1.86E+00	6.60E-03
U2AF1L4	NR_163171	1.43E-03	2.33E+00	1.74E-02
U2SURP	NM_001320219	1.94E-04	-1.55E+00	5.56E-03
UACA	NM_018003	4.32E-06	-2.63E+00	7.15E-04
UBA7	NM_003335	5.81E-03	1.76E+00	4.05E-02
UBAC1	NM_016172	2.12E-03	1.56E+00	2.21E-02
UBASH3A	NM_018961	7.47E-03	1.62E+00	4.76E-02
UBE2D3	NM_003340	1.99E-03	-1.53E+00	2.13E-02
UBL5	NM_001048241	9.68E-04	1.64E+00	1.37E-02
UBR1	NM_174916	3.05E-07	-1.87E+00	2.75E-04
UBR3	NM_172070	4.93E-05	-1.50E+00	2.46E-03
UBR5	NM_015902	8.53E-07	-1.62E+00	3.76E-04
UBR5-AS1	NR_125415	2.90E-03	-2.04E+00	2.66E-02
UBXN1	NM_015853	2.49E-03	1.82E+00	2.44E-02
UBXN11	NM_001077262	8.66E-04	7.77E+00	1.28E-02
UBXN6	NM_001171091	6.58E-03	2.30E+00	4.38E-02
UBXN7	NM_015562	4.04E-05	-1.56E+00	2.21E-03
UCHL5	NM_001199262	2.81E-04	-2.90E+00	6.80E-03
UCK1	NM_001261451	4.39E-04	1.78E+00	8.67E-03
UEVLD	NM_001261386	1.09E-04	7.45E+00	3.98E-03
UGDH	NM_003359	1.70E-03	-1.53E+00	1.94E-02
UGT8	NM_001128174	3.47E-03	-2.35E+00	2.95E-02
UHMK1	NM_175866	2.35E-04	-1.70E+00	6.18E-03
UHRF1BP1L	NM_015054	1.33E-03	-1.65E+00	1.67E-02
ULK3	NM_001284365	2.04E-03	3.21E+00	2.16E-02
UMAD1	NM_001302348	2.62E-03	-1.67E+00	2.52E-02
UNC119	NM_054035	2.36E-03	2.11E+00	2.36E-02
UNKL	NR_160279	8.63E-04	2.15E+00	1.28E-02
UPB1	NM_016327	2.36E-04	2.00E+00	6.19E-03
UPF3A	NR_148487	3.55E-03	-1.84E+00	2.99E-02
UPK3BL1	NM_001114403	7.15E-04	2.67E+00	1.15E-02
UQCC2	NM_032340	3.78E-05	1.84E+00	2.16E-03
UQCR10	NM_013387	4.18E-04	1.91E+00	8.41E-03
UQCR11	NM_006830	6.31E-04	2.81E+00	1.06E-02

UQCRHL	NM_001089591	2.07E-03	1.55E+00	2.17E-02
UQCRQ	NM_014402	1.11E-03	2.12E+00	1.48E-02
URM1	NM_001135947	1.40E-04	2.20E+00	4.53E-03
UROD	NR_158185	1.01E-03	2.10E+00	1.41E-02
UROS	NR_136676	6.78E-03	1.85E+00	4.47E-02
USE1	NM_018467	1.40E-03	1.99E+00	1.73E-02
USF1	NM_007122	9.62E-04	1.61E+00	1.36E-02
USF3	NM_001009899	9.61E-07	-2.31E+00	3.95E-04
USO1	NM_003715	1.53E-05	-1.61E+00	1.30E-03
USP13	NM_003940	4.23E-03	-1.51E+00	3.32E-02
USP15	NM_001351159	1.80E-03	-2.18E+00	2.01E-02
USP25	NM_013396	3.10E-05	-1.74E+00	1.96E-03
USP28	NM_001346259	1.17E-04	-2.42E+00	4.10E-03
USP3	NM_001256702	1.39E-03	1.94E+00	1.72E-02
USP33	NM_201626	4.06E-04	-2.29E+00	8.31E-03
USP35	NM_020798	7.21E-03	1.52E+00	4.65E-02
USP38	NM_001290325	3.38E-04	-3.30E+00	7.58E-03
USP47	NM_001282659	2.28E-07	-1.99E+00	2.38E-04
USP48	NM_001032730	6.69E-04	-1.67E+00	1.10E-02
USP53	NM_001371395	5.47E-05	-3.94E+00	2.61E-03
USP6NL	NM_001080491	4.08E-05	-2.06E+00	2.21E-03
USP7	NM_001286458	1.33E-05	-1.55E+00	1.20E-03
USP8	NM_001128610	3.08E-04	-1.57E+00	7.18E-03
USP9X	NM_001039590	2.42E-06	-1.93E+00	5.67E-04
USPL1	NM_001321532	1.83E-03	-1.72E+00	2.04E-02
UTP23	NM_032334	5.51E-05	-1.54E+00	2.61E-03
UTRN	NM_007124	1.92E-08	-1.91E+00	6.90E-05
VAMP1	NM_199245	1.28E-06	1.58E+00	4.37E-04
VAMP4	NM_003762	1.53E-03	-1.60E+00	1.81E-02
VAMP8	NM_003761	6.22E-05	1.83E+00	2.78E-03
VANGL1	NM_001172412	3.57E-03	-1.74E+00	3.00E-02
VASH1-AS1	NR_104184	6.70E-04	1.92E+00	1.10E-02
VAV3	NM_006113	2.82E-04	-2.11E+00	6.80E-03
VCAN	NM_001126336	3.87E-03	3.03E+00	3.15E-02
VCPIP1	NM_025054	3.89E-04	-1.59E+00	8.16E-03
VENTX	NM_014468	1.52E-04	1.87E+00	4.76E-03
VEZT	NR_038241	2.04E-03	-1.89E+00	2.16E-02
VGLL4	NM_001128219	3.16E-03	-1.69E+00	2.79E-02

VILL	NM_001370265	5.18E-04	2.38E+00	9.50E-03
VIPR1	NM_001251883	5.81E-04	1.74E+00	1.01E-02
VIRMA	NM_183009	1.24E-06	-2.97E+00	4.32E-04
VKORC1	NM_024006	6.24E-04	1.64E+00	1.06E-02
VMA21	NM_001363810	4.09E-03	-1.67E+00	3.25E-02
VMP1	NM_001329402	2.76E-03	2.02E+00	2.59E-02
VOPP1	NM_001321246	7.14E-04	1.57E+00	1.15E-02
VPS13A	NM_001018038	1.11E-03	-3.01E+00	1.49E-02
VPS13C	NM_020821	5.97E-06	-1.74E+00	8.28E-04
VPS25	NM_032353	1.94E-05	1.78E+00	1.48E-03
VPS29	NM_016226	4.71E-04	2.21E+00	9.04E-03
VPS41	NM_080631	2.28E-05	-2.85E+00	1.64E-03
VPS4A	NM_013245	3.72E-03	1.60E+00	3.08E-02
VPS52	NM_001289176	5.13E-03	2.22E+00	3.76E-02
VPS54	NM_016516	5.51E-03	-1.53E+00	3.94E-02
VPS8	NM_001349295	4.33E-03	1.53E+00	3.36E-02
VSTM1	NM_198481	1.67E-03	1.86E+00	1.92E-02
VWA8	NM_015058	1.55E-03	-2.15E+00	1.83E-02
WAC	NM_100264	4.77E-04	-1.52E+00	9.09E-03
WAPL	NM_015045	6.62E-06	-1.57E+00	8.39E-04
WASH5P	NR_033266	7.58E-08	1.81E+00	1.42E-04
WASH7P	NR_024540	3.13E-05	3.06E+00	1.97E-03
WASHC1	NM_182905	6.02E-03	2.02E+00	4.14E-02
WASHC2C	NM_001367416	3.96E-03	-2.35E+00	3.19E-02
WASHC3	NM_001301107	2.44E-03	1.59E+00	2.41E-02
WASHC5	NM_014846	1.60E-03	-1.68E+00	1.87E-02
WBP1	NM_012477	2.42E-03	2.13E+00	2.40E-02
WDFY1	NM_020830	1.13E-04	-1.68E+00	4.03E-03
WDHD1	NM_007086	5.12E-03	-1.66E+00	3.76E-02
WDR3	NM_006784	7.79E-04	-1.52E+00	1.20E-02
WDR35	NM_020779	5.33E-05	-1.69E+00	2.58E-03
WDR44	NM_019045	5.35E-06	-1.66E+00	7.98E-04
WDR45	NM_001029896	5.82E-03	1.64E+00	4.06E-02
WDR47	NM_001142551	3.71E-03	-1.62E+00	3.07E-02
WDR48	NM_001346227	1.24E-04	2.14E+00	4.23E-03
WDR59	NM_001324172	4.60E-03	-1.66E+00	3.50E-02
WDR6	NM_001320547	2.64E-04	2.07E+00	6.62E-03
WDR7	NM_052834	1.84E-06	-1.63E+00	4.95E-04

WDR81	NM_001163673	4.81E-04	3.49E+00	9.14E-03
WDR83OS	NM_016145	4.72E-03	1.77E+00	3.56E-02
WDR92	NM_001256476	5.54E-03	-2.31E+00	3.95E-02
WEE1	NM_001143976	7.59E-06	-2.20E+00	8.87E-04
WHAMMP1	NR_036650	3.08E-03	-1.79E+00	2.75E-02
WNK1	NM_014823	1.72E-06	-1.62E+00	4.95E-04
WRN	NM_000553	2.89E-03	-1.57E+00	2.66E-02
WSB1	NM_134265	1.83E-03	2.92E+00	2.04E-02
WWP1	NM_007013	1.36E-03	-1.51E+00	1.69E-02
XIAP	NM_001167	9.78E-07	-2.89E+00	3.95E-04
XPO7	NM_015024	2.97E-04	-1.52E+00	7.01E-03
XPR1	NM_004736	1.42E-03	-1.64E+00	1.74E-02
XRCC3	NM_005432	5.27E-03	3.63E+00	3.83E-02
XRCC6	NM_001288977	3.13E-03	4.38E+00	2.77E-02
XRN1	NM_001282857	1.01E-04	-1.76E+00	3.80E-03
YAF2	NR_135140	1.27E-03	-2.08E+00	1.62E-02
YBEY	NM_001314025	4.61E-07	2.57E+00	3.14E-04
YDJC	NM_001371350	8.07E-03	1.92E+00	4.99E-02
YES1	NM_005433	2.51E-03	-1.93E+00	2.45E-02
YIF1B	NM_001145462	5.71E-03	2.15E+00	4.02E-02
YPEL5	NM_001127401	2.68E-03	-2.03E+00	2.54E-02
YTHDC2	NM_022828	1.14E-03	-2.75E+00	1.51E-02
YTHDF3	NM_152758	3.48E-04	-2.38E+00	7.70E-03
ZBED6	NM_001174108	3.05E-05	-3.58E+00	1.94E-03
ZBTB1	NM_001123329	1.81E-04	-1.71E+00	5.31E-03
ZBTB16	NM_001354752	8.39E-04	-4.97E+00	1.26E-02
ZBTB17	NM_001287603	7.50E-03	1.90E+00	4.77E-02
ZBTB20	NR_121662	3.51E-04	-2.01E+00	7.74E-03
ZBTB21	NM_001320731	1.04E-06	-2.38E+00	4.01E-04
ZBTB3	NM_001363108	6.34E-05	2.03E+00	2.80E-03
ZBTB33	NM_006777	1.42E-03	-1.53E+00	1.74E-02
ZBTB37	NM_001369846	1.09E-03	-1.70E+00	1.48E-02
ZBTB38	NM_001350100	4.19E-06	-1.99E+00	7.03E-04
ZBTB40	NM_001330398	5.22E-03	1.81E+00	3.80E-02
ZBTB44	NM_001370219	2.11E-05	-1.64E+00	1.57E-03
ZBTB47	NM_145166	1.52E-03	1.79E+00	1.81E-02
ZBTB48	NM_001278647	5.11E-03	2.02E+00	3.76E-02
ZBTB6	NM_006626	8.49E-04	-1.55E+00	1.27E-02

ZC3H10	NM_001303125	7.52E-03	2.03E+00	4.77E-02
ZC3H11A	NM_001319239	9.72E-05	-2.22E+00	3.72E-03
ZC3H11B	NM_001355457	4.09E-04	-1.53E+00	8.33E-03
ZC3H12C	NM_033390	2.75E-03	-1.52E+00	2.59E-02
ZC3H13	NM_001330566	1.55E-04	-2.25E+00	4.82E-03
ZCCHC2	NM_017742	3.93E-04	-2.57E+00	8.20E-03
ZCCHC7	NM_001289120	6.84E-03	-1.73E+00	4.49E-02
ZCCHC8	NM_001350938	2.56E-05	2.86E+00	1.74E-03
ZDBF2	NM_001285549	1.12E-03	-2.09E+00	1.49E-02
ZDHHC16	NM_001287804	1.81E-03	2.13E+00	2.03E-02
ZDHHC17	NM_015336	3.41E-03	-1.65E+00	2.92E-02
ZDHHC21	NM_001354121	1.97E-04	-2.84E+00	5.61E-03
ZDHHC23	NM_001320466	6.82E-04	-2.53E+00	1.11E-02
ZDHHC3	NM_001349377	7.39E-04	1.75E+00	1.17E-02
ZDHHC4	NM_018106	2.68E-03	1.65E+00	2.54E-02
ZEB1	NM_001323676	1.66E-03	-1.78E+00	1.92E-02
ZEB2	NM_014795	1.20E-04	-1.81E+00	4.18E-03
ZFAND2A	NM_182491	1.68E-04	1.88E+00	5.07E-03
ZFAND2B	NM_001270998	8.73E-04	2.05E+00	1.29E-02
ZFAS1	NR_003606	1.98E-03	1.80E+00	2.12E-02
ZFP1	NM_153688	2.70E-03	-1.99E+00	2.56E-02
ZFP14	NM_020917	4.04E-03	-1.63E+00	3.23E-02
ZFP36L2	NM_006887	6.07E-07	-1.70E+00	3.62E-04
ZFR	NR_144318	6.30E-04	-1.82E+00	1.06E-02
ZFX	NM_003410	5.49E-05	-3.28E+00	2.61E-03
ZFYVE16	NM_001284237	9.58E-06	-4.93E+00	1.02E-03
ZFYVE19	NM_032850	5.35E-05	2.46E+00	2.59E-03
ZFYVE27	NM_001174122	1.00E-03	1.98E+00	1.40E-02
ZG16B	NM_145252	4.47E-05	2.70E+00	2.31E-03
ZGLP1	NM_001103167	1.43E-03	2.24E+00	1.74E-02
ZHX1	NM_007222	2.58E-04	-1.66E+00	6.54E-03
ZKSCAN1	NR_144477	2.59E-03	-1.91E+00	2.51E-02
ZKSCAN8	NM_001278119	1.08E-04	-1.82E+00	3.98E-03
ZMIZ1	NM_020338	6.80E-04	-1.59E+00	1.11E-02
ZMYM1	NM_024772	2.10E-03	-2.02E+00	2.20E-02
ZMYM2	NM_001353165	4.33E-03	-2.37E+00	3.36E-02
ZMYM4	NM_001350138	3.04E-04	-2.93E+00	7.13E-03
ZMYND11	NM_001370100	1.37E-03	-2.92E+00	1.70E-02

ZMYND15	NM_001136046	1.34E-03	2.86E+00	1.68E-02
ZNF106	NM_001366846	6.26E-06	-2.21E+00	8.32E-04
ZNF107	NM_001013746	9.26E-04	-2.17E+00	1.34E-02
ZNF12	NM_016265	9.24E-06	-2.17E+00	1.00E-03
ZNF124	NM_001297568	1.11E-03	-2.04E+00	1.49E-02
ZNF133	NM_001352451	1.89E-04	2.86E+00	5.46E-03
ZNF136	NM_003437	2.73E-03	-1.51E+00	2.57E-02
ZNF141	NM_001348278	3.31E-04	3.35E+00	7.49E-03
ZNF146	NM_001099639	1.60E-03	-1.72E+00	1.88E-02
ZNF148	NM_021964	5.53E-07	-1.88E+00	3.42E-04
ZNF160	NM_033288	5.10E-04	-1.88E+00	9.43E-03
ZNF174	NM_001347870	6.13E-03	1.53E+00	4.19E-02
ZNF189	NM_197977	4.83E-03	-1.82E+00	3.62E-02
ZNF197	NM_001323296	1.67E-03	-2.09E+00	1.92E-02
ZNF207	NM_003457	2.92E-03	-1.62E+00	2.67E-02
ZNF217	NM_006526	8.97E-07	-1.72E+00	3.80E-04
ZNF236	NM_001306089	6.37E-06	-1.86E+00	8.32E-04
ZNF24	NM_001308123	8.16E-04	-1.76E+00	1.24E-02
ZNF25	NM_001329657	1.94E-03	-1.96E+00	2.11E-02
ZNF254	NM_203282	8.10E-03	-1.92E+00	5.00E-02
ZNF257	NM_033468	4.74E-04	-3.12E+00	9.06E-03
ZNF266	NM_001370392	6.76E-03	-1.96E+00	4.45E-02
ZNF28	NM_006969	6.12E-04	-2.29E+00	1.05E-02
ZNF280C	NM_017666	4.30E-03	-1.51E+00	3.34E-02
ZNF280D	NM_001002843	2.00E-03	-2.05E+00	2.13E-02
ZNF281	NM_001281293	3.40E-04	-1.63E+00	7.60E-03
ZNF292	NM_015021	2.16E-04	-1.75E+00	5.91E-03
ZNF316	NM_001278559	8.09E-03	1.87E+00	4.99E-02
ZNF320	NM_001351775	2.24E-03	-2.04E+00	2.28E-02
ZNF333	NM_032433	2.80E-05	-1.86E+00	1.83E-03
ZNF33A	NM_001278176	1.47E-03	-2.13E+00	1.78E-02
ZNF33B	NM_001305037	3.19E-03	-1.80E+00	2.81E-02
ZNF347	NM_032584	6.49E-04	-1.76E+00	1.08E-02
ZNF37A	NM_001007094	1.44E-03	-1.56E+00	1.75E-02
ZNF429	NM_001346915	9.79E-04	-2.86E+00	1.38E-02
ZNF43	NM_003423	6.94E-03	-1.65E+00	4.53E-02
ZNF430	NM_025189	1.24E-04	-1.70E+00	4.23E-03
ZNF431	NR_138053	3.88E-05	-2.09E+00	2.19E-03

ZNF433-AS1	NR_134930	6.68E-04	1.96E+00	1.10E-02
ZNF442	NM_001363774	5.63E-03	-1.84E+00	3.99E-02
ZNF486	NM_052852	4.26E-04	-1.83E+00	8.52E-03
ZNF493	NM_001076678	3.70E-03	-1.90E+00	3.07E-02
ZNF506	NM_001099269	3.67E-05	-1.75E+00	2.13E-03
ZNF507	NM_014910	7.92E-04	-1.56E+00	1.21E-02
ZNF511	NM_145806	1.72E-03	2.04E+00	1.96E-02
ZNF513	NM_144631	3.08E-03	2.35E+00	2.75E-02
ZNF517	NR_134284	1.40E-04	1.88E+00	4.52E-03
ZNF518A	NM_001278525	5.89E-05	-2.91E+00	2.69E-03
ZNF525	NR_145445	5.94E-04	-2.64E+00	1.03E-02
ZNF529	NM_020951	2.35E-03	-1.70E+00	2.36E-02
ZNF570	NM_001321992	6.28E-04	-2.35E+00	1.06E-02
ZNF580	NM_016202	3.39E-03	1.70E+00	2.91E-02
ZNF581	NM_016535	4.34E-03	1.74E+00	3.36E-02
ZNF585A	NM_152655	1.93E-06	-3.18E+00	5.03E-04
ZNF595	NM_182524	3.90E-05	-2.28E+00	2.19E-03
ZNF611	NM_001161500	3.85E-03	-1.63E+00	3.14E-02
ZNF619	NM_001145093	1.68E-03	-1.74E+00	1.93E-02
ZNF621	NM_198484	2.98E-04	-1.83E+00	7.03E-03
ZNF623	NM_014789	3.26E-03	-1.53E+00	2.84E-02
ZNF624	NM_020787	4.42E-03	-1.59E+00	3.41E-02
ZNF638	NM_001014972	6.28E-06	-1.72E+00	8.32E-04
ZNF644	NM_201269	4.32E-04	-1.90E+00	8.59E-03
ZNF652	NM_014897	9.19E-06	-1.72E+00	1.00E-03
ZNF654	NM_001350134	5.95E-04	-2.75E+00	1.03E-02
ZNF655	NM_001085368	1.16E-03	-1.73E+00	1.53E-02
ZNF664	NM_152437	3.96E-05	-1.74E+00	2.20E-03
ZNF669	NM_001142572	3.38E-04	-1.52E+00	7.58E-03
ZNF674	NM_001190417	5.69E-03	-1.70E+00	4.01E-02
ZNF692	NM_017865	2.45E-04	1.83E+00	6.36E-03
ZNF699	NM_198535	3.48E-04	-1.61E+00	7.70E-03
ZNF721	NM_133474	1.82E-03	-1.66E+00	2.03E-02
ZNF737	NM_001159293	3.82E-06	-2.04E+00	7.03E-04
ZNF749	NM_001023561	4.92E-03	-2.29E+00	3.67E-02
ZNF75D	NM_007131	2.27E-03	-1.51E+00	2.31E-02
ZNF76	NM_001292032	6.91E-03	1.74E+00	4.51E-02
ZNF765	NM_001040185	1.42E-03	-1.63E+00	1.74E-02

ZNF770	NM_014106	3.55E-05	-1.70E+00	2.10E-03
ZNF772	NM_001144068	5.52E-03	-2.21E+00	3.94E-02
ZNF778	NM_001201407	6.09E-04	-2.65E+00	1.04E-02
ZNF780A	NM_001142578	1.51E-03	-2.83E+00	1.80E-02
ZNF783	NM_001195220	8.80E-04	1.61E+00	1.30E-02
ZNF784	NM_203374	1.22E-03	1.86E+00	1.58E-02
ZNF790	NM_001242800	7.35E-03	-3.18E+00	4.71E-02
ZNF800	NM_176814	2.46E-04	-1.60E+00	6.36E-03
ZNF808	NM_001039886	4.08E-04	-1.79E+00	8.33E-03
ZNF81	NM_007137	6.60E-06	-1.72E+00	8.39E-04
ZNF831	NM_178457	2.29E-03	-1.68E+00	2.32E-02
ZNF844	NR_134326	1.48E-03	-2.65E+00	1.78E-02
ZNF845	NM_001321523	7.10E-05	-3.40E+00	3.00E-03
ZNF85	NM_003429	1.10E-03	-1.92E+00	1.48E-02
ZNF852	NM_001287349	2.87E-03	-1.63E+00	2.64E-02
ZNF860	NM_001137674	2.78E-04	-2.73E+00	6.77E-03
ZNF862	NM_001099220	3.56E-04	1.52E+00	7.77E-03
ZNF888	NM_001310127	1.97E-04	-1.69E+00	5.61E-03
ZNF90	NM_007138	8.04E-04	-2.39E+00	1.22E-02
ZNF91	NM_001300951	3.80E-05	-2.46E+00	2.16E-03
ZNF92	NM_152626	1.23E-04	-2.47E+00	4.22E-03
ZNHIT1	NM_006349	1.77E-03	2.07E+00	2.00E-02
ZNHIT3	NR_104010	1.27E-04	2.94E+00	4.27E-03
ZNRD1	NM_170783	9.74E-05	1.64E+00	3.73E-03
ZSCAN12	NM_001368124	7.37E-04	-1.90E+00	1.17E-02
ZSCAN16-AS1	NR_103456	5.41E-06	1.70E+00	8.01E-04
ZSCAN29	NM_152455	5.67E-05	-1.61E+00	2.65E-03
ZSWIM7	NM_001042698	1.78E-04	1.54E+00	5.26E-03
ZSWIM8-AS1	NR_038357	1.09E-04	1.63E+00	3.98E-03
ZXDA	NM_007156	4.92E-03	-1.54E+00	3.67E-02
ZZZ3	NM_015534	3.67E-03	-1.60E+00	3.05E-02
