# Supplementary material for: Profiling Immunological Phenotypes in Individuals During the First Year After Traumatic Spinal Cord Injury: A Longitudinal Analysis
Source: J Neurotrauma. 2023 Nov 30;40(23-24):2621–37. doi: 10.1089/neu.2022.0500 (PMC10722895; doi:10.1089/neu.2022.0500)

**SUPPLEMENTARY FIG. S2**. PCA analysis. **(A1)** Principal component analysis (PCA) illustrates that the first two components explain approximately 70% of variation in gene expression (Y-axis) among participants. Time after injury is indicated on the X axis. **(A2–A4)** Box and whisker plots show relative distribution of samples over time in each PC. Boxes indicate range of Q1 to Q3, line indicates median, and whiskers indicate minimum to maximum. For the top 175 genes loading PC1 (eigenvalue ³0.85), top categories (by smallest *p*-value) identified by **(B)** Gene Ontology biological process (GOBP) are shown, and **(C)** Human Gene Atlas database identified CD8+ and CD4+ T-cell genes as significantly enriched. The corresponding gene list is in Supplementary Table S2.


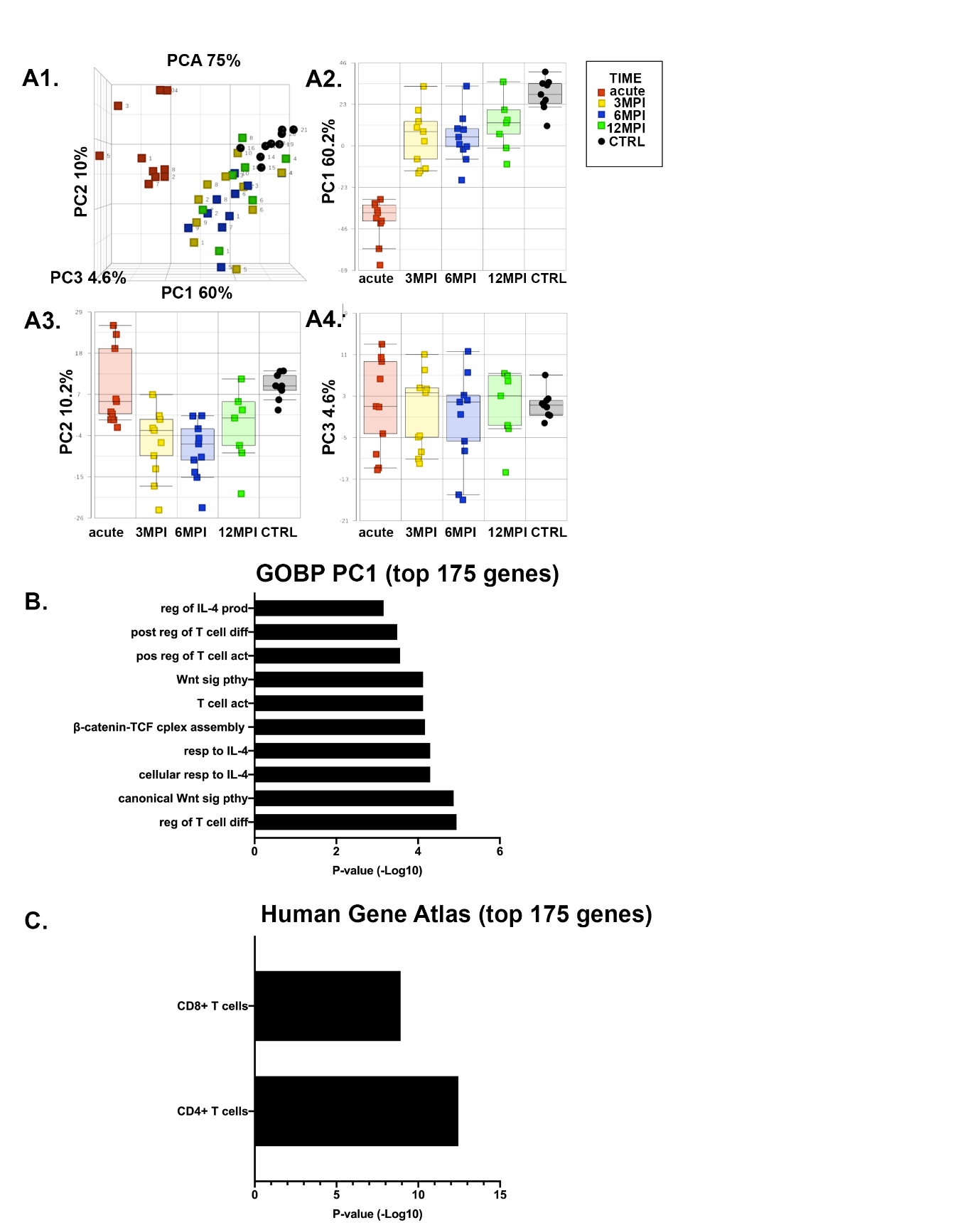

Supplement: Supplemental data [file Suppl_FigureS2.docx]
