# Supplementary material for: Profiling Immunological Phenotypes in Individuals During the First Year After Traumatic Spinal Cord Injury: A Longitudinal Analysis
Source: J Neurotrauma. 2023 Nov 30;40(23-24):2621–37. doi: 10.1089/neu.2022.0500 (PMC10722895; doi:10.1089/neu.2022.0500)

**SUPPLEMENTARY FIG S3.** Gene Ontology biological process (GOBP) analysis of differentially expressed (DE) genes that were unique to acute, 3 months post-injury (MPI), or 6 MPI. DE genes that were upregulated at the respective study visits are shown **(A–C)**. DE genes that were downregulated at the respective study visits are also shown (**D–F)**.


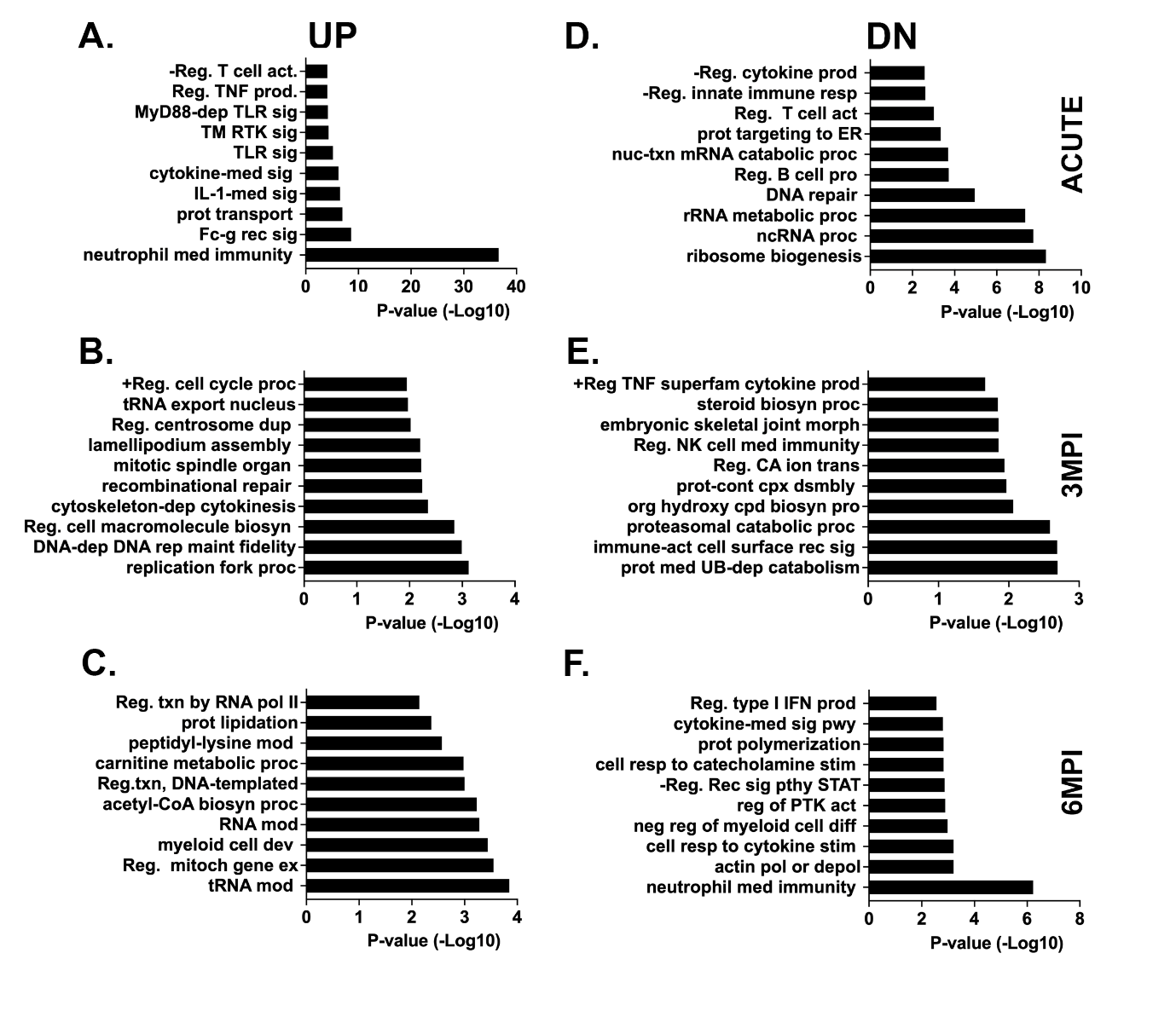

Supplement: Supplemental data [file Suppl_FigureS3.docx]
